# Revealing microbial consortia that interfere with grapevine downy mildew through microbiome epidemiology

**DOI:** 10.1186/s40793-025-00691-9

**Published:** 2025-03-27

**Authors:** Paola Fournier, Lucile Pellan, Aarti Jaswa, Marine C. Cambon, Alexandre Chataigner, Olivier Bonnard, Marc Raynal, Christian Debord, Charlotte Poeydebat, Simon Labarthe, François Delmotte, Patrice This, Corinne Vacher

**Affiliations:** 1 INRAE, Bordeaux Sciences Agro, ISVV, SAVE, Villenave-d’Ornon, France; 2 School of Biosciences, Birmingham Institute of Forest Research, Institute of Microbiology and Infection, University of Birmingham, Birmingham, UK; 3 INRAE, BIOGECO, Univ. Bordeaux, Cestas, France; 4 IFV, Blanquefort, France; 5 Bordeaux Sciences Agro, INRAE, ISVV, SAVE, Villenave-d’Ornon, France; 6 UMR AGAP Institut, Univ. Montpellier, CIRAD, INRAE, Institut Agro, Montpellier, France

**Keywords:** Microbial ecology, Disease biocontrol, Oomycete, *Vitis vinifera*, *Plasmopara viticola*, Soil, Phyllosphere, Endosphere, Metabarcoding, Differential abundance analysis

## Abstract

**Background:**

Plant and soil microbiomes can interfere with pathogen life cycles, but their influence on disease epidemiology remains understudied. Here, we analyzed the relationships between plant and soil microbiomes and long-term epidemiological records of grapevine downy mildew, a major disease caused by the oomycete *Plasmopara viticola*.

**Results:**

We found that certain microbial taxa were consistently more abundant in plots with lower disease incidence and severity and that the microbial community composition could predict disease incidence and severity. Microbial diversity was not strongly linked to epidemiological records, suggesting that disease incidence and severity is more related to the abundance of specific microbial taxa. These key taxa were identified in the topsoil, where the pathogen’s oospores overwinter, and in the phyllosphere, where zoospores infect leaves. By contrast, the leaf endosphere, where the pathogen’s mycelium develops, contained few taxa of interest. Surprisingly, the soil microbiota was a better predictor of disease incidence and severity than the leaf microbiota, suggesting that the soil microbiome could be a key indicator of the dynamics of this primarily aerial disease.

**Conclusion:**

Our study integrates long-term epidemiological data with microbiome profiles of healthy plants to reveal fungi and bacteria relevant for the biocontrol of grapevine downy mildew. The resulting database provides a valuable resource for designing microbial consortia with potential biocontrol activity. The framework can be applied to other crop systems to guide the development of biocontrol strategies and reduce pesticide use in agriculture.

**Supplementary Information:**

The online version contains supplementary material available at 10.1186/s40793-025-00691-9.

## Background

Chemical pesticides are the most commonly used method for protecting crops against pathogens [1]. However, legislation is increasingly restricting their use because of their significant negative impacts on the environment and human health [2–4]. In this context, microbial biocontrol strategies are promising alternatives for enhancing plant protection against pests and diseases while reducing the use of chemical products [5, 6]. These strategies involve the use of microorganisms or their byproducts to control pests and diseases through direct interactions such as antibiosis, predation, parasitism, and competition, or through indirect interactions such as the modulation of plant immunity [6, 7]. Harnessing the diversity of plant microbiota could also be an option, as several studies have established a link between ecosystem functioning and microbial diversity [8–10], validating the 'Biodiversity-Ecosystem Functioning' (BEF) theory [11]. The challenge lies in identifying the combination of microbial taxa involved in plant protection, among the multitude of taxa that constitute the plant microbiota.

Identifying protective microbial taxa is crucial for diseases that require frequent fungicide applications, as it can lead to significant reductions in pesticide use. Grapevine downy mildew, caused by the oomycete *Plasmopara viticola* [12, 13], is one such disease [13, 14]. *Plasmopara viticola* (Berk. & M.A. Curtis) Berl. & De Toni is a biotrophic and obligate parasite of grapevine [15] originating from North America [12, 16]. It was introduced to Europe in the mid-nineteenth century, where it destroyed a large proportion of French vineyards due to the high susceptibility of *Vitis vinifera* [17, 18]. This disease is now reported in most wine-producing regions of the world [16, 19] and has a significant economic impact [19–21]. In temperate climates, *P. viticola* survives the winter through sexual reproduction, producing oospores in grapevine leaves during the fall. The oospores then overwinter in the leaf litter and soil. In the following spring, the oospores germinate to produce macrosporangia containing zoospores, which are dispersed by wind and rain to infect grapevine leaves and other receptive green tissues. Primary infections occur when zoospores, originating from oospores (i.e., primary inoculum), enter leaf tissues through stomata. Secondary infection cycles are initiated by the production of sporangia on the lower surface of infected leaves by asexual reproduction. The sporangia release zoospores (i.e., secondary inoculum), which initiates a new cycle of infection [12, 13, 22]. Without effective disease control measures, favorable weather conditions can trigger multiple cycles of primary and secondary infections within the growing season, potentially leading to complete crop loss [12].

Grapevines naturally host a diverse microbiota, including leaf epiphytes, which develop on the leaf surface in the microbial habitat known as the phyllosphere [23, 24], and leaf endophytes, which inhabit the internal leaf tissues in the microbial habitat known as the endosphere [25]. Numerous microorganisms isolated from these two habitats have been tested, as single strains [12, 13, 21, 26–28] or in combination [23], for their antagonistic activity against downy mildew, and some have emerged as promising candidates for biocontrol of the asexual stage of *P. viticola*. These microorganisms serve as a primary line of defense by inhibiting zoospore motility, adhesion, and penetration through antibiosis [29–33]. They can also reduce sporangia production and sexual reproduction by interacting directly with the pathogen’s mycelium through hyperparasitism and antibiosis [34–36]. Finally, they can inhibit sporangiophore formation and germination through hyperparasitism and antibiosis [36–41]. Despite these promising results, only one bacterial strain, *Bacillus amyloliquefaciens* FZB24, has been formally registered in France for downy mildew biocontrol [42].

To date, limited research has explored the relationship between the soil microbiome and downy mildew oospores. A laboratory study demonstrated the antagonistic activity of *Acremonium byssoides* against *P. viticola* oospores through hyperparasitism and antibiosis [43]. Similarly, bacteria, fungi, and yeasts collected from abandoned vineyards effectively inhibited the germination of oospores overwintering under natural conditions [44]. Additional research on other oomycete species has shown that various bacterial and fungal taxa from natural soils can parasitize oospores of several oomycete species, including *Pythium* sp., *Phytophthora* sp., and *Aphanomyces euteiches* [45–47].

In this study, our goal was to uncover microbial consortia with the potential to protect vineyards from *P. viticola* and ultimately reduce reliance on chemical fungicides. We integrated microbial community data with long-term epidemiological data, an emerging field known as microbiome epidemiology [48]. We also evaluated the predictive power of microbial data in epidemiology, which remains an underexplored aspect in the context of plant disease management [49]. We tested four hypotheses: (1) the diversity of leaf and soil microbial communities is higher in plots with low disease severity and incidence; (2) plots with low disease severity and incidence harbor a greater abundance of specific microbial taxa that are hypothesised to play a role in pathogen regulation; (3) downy mildew severity and incidence can be predicted from microbiota data; and (4) the leaf microbiota is a better predictor of disease severity and incidence compared to the soil microbiota, as the symptoms appear on the aerial plant parts.

## Materials and methods

To test these hypotheses, a two-year leaf and soil sampling campaign was conducted in 14 vineyard plots in the Bordeaux region (Nouvelle-Aquitaine, France). These plots, selected based on long-term epidemiological records, comprised seven pairs with contrasting downy mildew incidence and severity. Sampling targeted young emerging leaves (at the phenological stage of 2–3 spread leaves) and topsoil (upper 5 cm). This study was conducted in the spring, at the beginning of the growing season, to identify the protective microbiota likely to be present on leaves during colonization by early *P. viticola* zoospores, as well as the microbiota likely to be in contact with germinating oospores in the soil.

### Selection of study sites based on epidemiological records

The study sites were selected based on epidemiological records provided by the *Institut Français de la Vigne et du Vin* (IFV) using a network of 1200 Untreated Controls (hereafter referred to as UCs, Fig. S1) monitored since 2002. Untreated Controls are generally composed of 2*5 vines in staggered rows that do not receive any fungicide treatment. The UC can either remain in the same location over the years or be moved each year, i.e., different vines can be selected as UCs. The UC can be located on the edge or in the center of the plot, depending on the region and the grower's preference. The incidence and severity of downy mildew attacks on vine leaves and bunches are observed weekly during the growing season. Leaf attack incidence is defined as the number of symptomatic leaves divided by the total number of leaves observed [50]. The severity of leaf attack is defined as the sum of the percentage of symptomatic leaf area divided by the total number of leaves observed [50]. Similar definitions are used for bunches. They are carried out by vine growers or IFV technicians, and are visual estimates expressed as percentages (0–100%)[50].

Based on these epidemiological records (downloaded from[51]), we selected pairs of vineyard plots that contrasted in incidence and severity of downy mildew infection, but were as similar as possible in terms of genetic material, geographic location, and management type. We used 5 criteria to select pairs of plots. To belong to the same pair, plots must be planted with the same grape variety (Criterion 1—Same Variety), and their geographical distance must not exceed 10 km (Criterion 2—Proximity). The type of management (organic, conventional or biodynamic) must be the same (Criteria 3—Same Management). In addition, the UCs within the two plots must have been monitored by IFV for at least 4 common years (Criteria 4—Common Records), including at least one recent year (i.e., 2020 or 2021 for the 2022 sampling campaign and 2021 or 2022 for the 2023 sampling campaign). Finally, the epidemiological records must be contrasted (Criterion 5—Contrasted Epidemiology). This means that the area under the disease progress curve (AUDPC) of one of the two plots must be higher for the 4 epidemiological variables measured by IFV (severity of symptoms on leaves, incidence of symptoms on leaves, severity of symptoms on bunches, incidence of symptoms on bunches) in at least 80% of the years of common epidemiological surveillance.

Within each pair of plots, the plot with the highest disease incidence and severity was considered a plot with high downy mildew incidence and severity, while the plot with the lowest disease incidence and severity was considered a plot with low downy mildew incidence and severity. The plot selection procedure was applied twice. To select plots to be sampled in 2022, we used epidemiological data collected between 2002 and 2021. Then, epidemiological data collected between 2002 and 2022 were used to select plots to be sampled in 2023.

### Sampling design

Two sampling campaigns were conducted. The first campaign took place from April 18 to May 2, 2022 and the second campaign took place from April 11 to April 26, 2023. Both campaigns took place at the phenological stage of 2–3 spread leaves, prior to the application of any fungicide treatments.

In the 2022 campaign, samples of young leaves and topsoil were collected from four areas within each plot: the center of the plot (CEN), the edge of the plot (EDG), and the untreated controls (UCs) used by the IFV to monitor disease dynamics in 2021 and 2022 (UC1 and UC2). In the 2023 campaign, samples of young leaves and topsoil were collected only from the center of the plot (CEN). Both the CEN and EDG areas received fungicide treatments the year before sampling. By contrast, UC1 did not receive fungicide treatments the year before sampling. It may or may not be located at the edge of the plot, depending on the grower’s choice. The within-plot location and fungicide treatments the year before sampling were variable for UC2. The edge of the plot (EDG) was selected to be as far away from UC1 and UC2 as possible to capture within-plot variability.

Within each sampling area, four adjacent vines from the same row were selected. These vines were representative of the age and general condition of the plot. From each vine, six leaves distributed across the entire plant were collected and pooled into a single composite sample (Supplementary Fig. S2). For soil sampling, three 20 × 20 cm quadrats were defined at a distance of 20 cm from the trunk of each vine (Supplementary Fig. S2). Two quadrats were directly under the row of vines, and one was in the interrow space. The top 5 cm of soil from each quadrat was collected, mixed, and placed into a bag to form a composite sample. Litter and grasses, when present, were collected along with the topsoil and later removed during sieving if they did not pass through, while materials that passed through were retained.

All the samples were collected using surgical gloves and disinfected tools (scissors, shovels, trowels, picks). Before sampling a new plot or a new area within the plot, gloves were changed and tools were disinfected with bleach (3.6% chlorine content) and 70% ethanol. The samples were placed in sterile plastic bags (NASCO™, Whirl–Pak®, USA) and kept on ice in a cooler. The samples were brought back to the laboratory on the same day. In the laboratory, the samples were stored at 4 °C until processing. Leaves were processed the following day, and the soils were processed the following week.

### Sample processing

The day after sample collection, the leaves were processed to separate the epiphytic microbiota (inhabiting the phyllosphere) from the endophytic microbiota (inhabiting the endosphere) under a Class II Biosafety Cabinet. To isolate epiphytes, each collection plastic bag was supplemented with 50 mL of sterile leaf washing buffer (0.9% NaCl, 0.01% Tween 80) (MilliporeSigma™, Calbiochem™, PBS-TWEEN™ Tablets, USA) and incubated on an orbital shaker at 150 rpm for 1 h. The buffer was then centrifuged (5525 × *g*, 4 °C) for 20 min into 50 mL tubes (Corning Inc., Falcon® Tubes, USA). After the supernatant was removed, the remaining 2 mL, including the pellet, was transferred to a 2 mL tube and centrifuged again (14,000 rpm, 4 °C) for 20 min. The supernatant was discarded, and the pellet was stored at -80 °C.

For endophyte collection, washed leaves were surface sterilized by immersion in 10% calcium hypochlorite solution for 10 min, rinsed in two sterile water baths for 1 min each, dried by placement on sterile filter paper for a few minutes, and transferred to 2 mL tubes with two sterilized 5 mm diameter chromium steel balls. The surface-sterilized leaves were then freeze-dried overnight, ground with TissueLyser II (QIAGEN®, Germany) with 30-s grinding intervals at 30 Hz, separated by 1-min pauses, and stored at room temperature. In addition, to validate the surface sterilization process, water washes from the final rinse were collected, and microbial DNA was detected by extraction, PCR amplification, and sequencing, following the same procedures as those used for real samples (detailed below).

The soil samples were sieved to 2 mm no later than 10 days after harvest. To avoid cross-contamination, the gloves were changed, and sieves were disinfected between samples from different plots and different areas. The sieves were cleaned by removing the soil with tap water, disinfected with 3.6% bleach, washed with water, sprayed with 70% ethanol, and finally air dried. After sieving, each soil sample was homogenized by hand. Two 50 mL tubes were subsampled from the homogenized soil. One tube was kept at room temperature for physicochemical analysis, whereas the other, intended for metabarcoding analysis, was freeze-dried overnight prior to DNA extraction.

### Soil physicochemical analysis

The four soil samples collected from the same sampling area of the same plot were pooled before being sent to the *Laboratoire d'Analyses des Sols d'Arras*, France [52]. The analysis included measurements of the three-fragment particle size, C/N ratio, pH, organic matter content, and content of total limestone, total carbon, total nitrogen and total organic carbon.

### DNA extraction

DNA extraction, amplification, library preparation and sequencing were performed at the Genome Transcriptome Facility of Bordeaux (France). Extractions were performed in a confined laboratory dedicated to environmental DNA studies. We used the PowerSoil® Pro Kit (QIAGEN®, Germany) for DNA extraction from the soil and phyllosphere samples and the DNeasy® Mini Plant Kit (QIAGEN®, Germany) for the endosphere samples. These kits were used in individual tubes to limit cross-contamination between samples. For soil DNA extraction, 200 mg of homogenized soil was used, whereas for endosphere DNA extraction, 10 mg of leaf powder was used. Phyllosphere DNA was extracted directly from the frozen pellet. The extractions were performed according to the manufacturer's instructions, with slight modifications. To reduce handling, PowerBeads® from the PowerSoil® Pro Kit were transferred directly into 2 mL collection tubes containing soil and phyllosphere material instead of using PowerBead® Pro Tubes. In addition, to facilitate cell lysis, the tubes were incubated at 65 °C for 10 min after the addition of CD1 lysis buffer and prior to the homogenization step. The DNA was eluted in 100 µL using the DNeasy® Mini Plant Kit for endosphere samples.

This DNA was then used for bacterial and fungal community profiling by short- and long-read sequencing and for *P. viticola* quantification by digital droplet PCR.

### Short-read sequencing of bacterial and fungal communities

To characterize fungal communities, the internal transcribed spacer 1 (ITS1) region of the fungal nuclear ribosomal DNA (nrDNA) ITS gene was amplified using the ITS1F forward primer [53] and the ITS2 reverse primer [54]. We also tested the primer set ITS1catta—ITS2ngs [55, 56], which targets the nrDNA ITS gene of both fungi and oomycetes and has been successfully used in grapevine [57]. However, we ultimately did not use this primer set because of its lack of specificity in our samples (Supplementary Methods S1 and Fig. S3 and S4). For bacterial communities, the V5-V6 region of the bacterial 16S ribosomal RNA (rRNA) gene was amplified using the chloroplast-excluding forward primer 799f [58] and the reverse primer 1115r [59]. PCRs were performed with 5 μL of template DNA (corresponding approximately to a mean of 16 ± 8.5 ng (standard error) for topsoil samples, 71 ± 31 ng for endosphere samples, and < 1 ng for phyllosphere samples), 2 μL of each primer (3 µM concentration), 4 μL of HOT FIREPol® MultiPlex Mix (5X concentration) (Solis BioDyne, Lithuania) and ultrapure water to a total volume of 20 μL. The thermocycling conditions for nrDNA ITS gene amplification were as follows: 95 °C/15 min; 95 °C/30 s, 56 °C/30 s, 72 °C/30 s for 35 cycles; and 72 °C/5 min. The thermocycling conditions for 16S rRNA gene amplification were as follows: initial denaturation at 95 °C for 15 min, followed by 30 cycles at 95 °C for 30 s, 49 °C for 30 s, and 72 °C for 30 s, with a final extension at 72 °C for 5 min. Amplification was confirmed by electrophoresis on 2% agarose gels.

We included four types of controls in the PCR plates: negative extraction controls, negative PCR controls, positive PCR controls, and negative sequencing controls. Negative extraction controls were obtained by performing DNA extractions in empty collection tubes (without samples) to detect and analyze potential contamination during the DNA extraction process. For negative PCR controls, ultrapure water was used instead of a DNA template to detect and analyze potential contamination during the PCR process. Positive PCR controls were used to verify that the PCRs worked well for each plate and to detect cross-contamination. They consisted of pure DNA from two marine fungi (*Candida ocean*i and *Wallemia sebi*) and bacteria (*Sulfitobacter pontiacus* and *Vibrio splendidus*) for PCRs performed on the nrDNA ITS and 16S rRNA genes, respectively. These strains were chosen because they are unlikely to be present in our samples, which helps to detect and evaluate the rate of cross-contamination between wells of the same plate. Negative sequencing controls, consisting of empty wells, were filled with ultrapure water to serve as controls for the second PCR (indexing step). The PCR plate maps were designed according to the guidelines of [60]. Each plate had 2–3 extraction negative controls, 2–3 PCR negative controls, and 2–3 PCR positive controls randomly distributed on the plate. Negative sequencing controls were placed in a diagonal line to estimate tag switch rates for as many index combinations as possible.

The second PCR was used to add Illumina® adapters and tags and was performed by the Genome Transcriptome Facility of Bordeaux (France) following the 16S rRNA gene Metagenomic Sequencing Library Preparation protocol (Illumina®). After this indexing step, the libraries were quantified by fluorescence using the Quant-iT™ dsDNA High Sensitivity Assay Kit (Thermo Fisher Scientific™, USA) and pooled to an equimolar concentration. The final pool was purified using a 300–700 bp size selection on a Pippin Prep® (Sage Science™, USA) to remove large nonspecific fragments and the remaining adapters. Pool size was assessed on a TapeStation 4200 system (Agilent Technologies®, USA), and the concentration was estimated by qPCR on an LC480 II system (Roche®, Switzerland) using the QIAseq® Library Quant Kit (QIAGEN®, Germany). Sequencing was performed on a NextSeq® 2000 system (Illumina®, USA) using P1 reagents and 301:10:10:301 cycles. The samples were split into three sequencing runs. The first run included all 2022 phyllosphere samples and half of the 2022 soil samples. The second run included all 2022 endosphere samples and the remaining soil samples. The third run included all 2023 samples. In each run, nrDNA ITS and 16S rRNA amplicons were mixed a 1:1 ratio. Adapter removal and demultiplexing were performed at the sequencing facility using the Cutadapt tool.

### Long-read sequencing of bacterial communities

To improve the taxonomic characterization of the bacterial communities, we also sequenced the entire 16S rRNA gene (V1-V9) using PacBio® HiFi technology. Sequencing was performed at the GeT-IT Genotoul facility (Toulouse, France). We prepared one DNA pool per microbial habitat (soil, leaf endosphere and phyllosphere). Amplification of the full-length 16S rRNA gene was performed using the universal primer set 27f (AGRGTTYGATYMTGGCTCAG) and 1492r (RGYTACCTTGTTACGACTT) [61]. Amplification was performed at 20 cycles with an annealing temperature of 57 °C. SMRTbell® libraries were prepared at the GeT-PlaGe Core Facility (Toulouse, France) from the amplified DNA by blunt-ligation according to the PacBio® protocol "Preparing SMRTbell® Libraries using PacBio® Barcoded Overhang Adapters for Multiplexing Amplicons" (Pacific Biosciences®). At each step, the DNA was quantified using the Qubit® dsDNA HS Assay Kit (Life Technologies™), and the amplicon size was assessed using the DNF-474-0500_HS NGS Fragment Analysis Kit (Agilent Technologies®). The purification steps were performed using AMPure® PB Beads (PacBio®). Purified SMRTbell® libraries of approximately 1560 bp were then sequenced using Binding Kit 3.1 and Sequencing Kit 2.0 by diffusion loading on a SMARTcell® of the PacBio Sequel® II instrument at 70 pM with a 0.8-h preextension and a 15-h run. Circular consensus sequence (CCS) reads were generated from the raw PacBio® sequencing data using the standard software tools provided by the manufacturer (Pacific Biosciences®), with minPasses = 3 and minPredictedAccuracy = 0.999 in SMRT Link software (release_12.0.0.177059).

### Quantification of the downy mildew inoculum in the soil samples

The downy mildew inoculum in all soil samples was quantified using droplet digital PCR (ddPCR) at the Transcriptomic-qPCR facility of the Neurocenter Magendie (Bordeaux, France). The primers used for the ddPCRs were Giop F (TCCTGCAATTCGCATTACGT) and Giop R (GGTTGCAGCTAATGGATTCCTA) [62]. PCRs were prepared with the required QX200™ ddPCR™ Supermix for Probes (Bio-Rad®, USA) at a final concentration of 750 nM for each primer and 500 nM for the TaqMan® probe (Giop P-VIC: TCGCAGTTCGCAGCGTTCTTCA) to a final volume of 22 µL. Then, 4 µL of each sample was added to the reaction mixture. Each reaction was loaded into a sample well of an 8-well disposable cartridge (Bio-Rad®, USA), and 70 µL of droplet generator oil (Bio-Rad®, USA) was added to the oil wells of the cartridge. Droplets were formed in the QX200™ Droplet Generator (Bio-Rad®, USA), transferred to a 96-well PCR plate, heat-sealed with foil in a PX1™ PCR Plate Sealer (Bio-Rad®, USA), and amplified using an Eppendorf™ Mastercycler™ Nexus Gradient (Eppendorf™, Germany) (95 °C primary denaturation/activation for 5 min, followed by 40 cycles of 95 °C for 30 s, 60 °C for 1 min, and 72 °C for 30 s, followed by 98 °C for 10 min). PCRs were analyzed using the QX200™ Droplet Reader (Bio-Rad®, USA), and data analysis was performed using QuantaSoft™ software (version 1.7; Bio-Rad®, USA).

### Bioinformatics

Bioinformatic analysis of Illumina and PacBio sequence data was performed using version 4.1 of the FROGS pipeline [63] on the Genotoul remote server [64]. For Illumina sequence data, separate analyses were performed for each barcode region (nrDNA ITS or 16S rRNA genes). The FROGS pipeline first assembled raw forward and reverse reads for each sample into paired-end reads with a minimum overlap of 10 nucleotides and a maximum mismatch of 0.1 using the VSEARCH algorithm [65]. For fungal data, unmerged reads were artificially joined by inserting a 100 N sequence between the forward and reverse reads [63]. The primers were removed using Cutadapt [66]. Sequences containing ambiguous nucleotides or whose size did not match the expected amplicon size (between 50 and 600 bp for the rDNA ITS gene and 250–400 bp for the 16S rRNA gene) were filtered out. The sequences were then dereplicated and clustered using SWARM [67] with a local clustering threshold parameter set to 1 and the 'fastidious' option enabled [68]. These clusters of sequences are equivalent in construction to Amplicon Sequence Variants (ASVs) [69] and are hereafter referred to as ASVs. Chimeras were detected and removed using VSEARCH [65]. A stringent filtering step was applied, which consisted of retaining only ASVs representing at least 0.00005% of the sequences in the dataset. For the fungal data, the highly variable part of the ITS1 region was extracted using ITSx [70]. PacBio HiFi 16S rRNA raw sequences were processed in a similar manner, specifying that the data were from long-read sequencing and adjusting the expected amplicon size to 500–2000 bp.

Taxonomic assignments of bacterial and fungal ASVs obtained from Illumina sequence data were performed using the SILVA 138.1 [71] and UNITE Fungi 8.3 [72] reference databases, respectively, using both the RDPClassifier [73] and BLASTn + algorithms [74] (Supplementary Methods S3). In addition, we performed taxonomic assignments using BLASTn + against a custom reference database. This custom database consisted of both sequences from bacterial ASVs generated using PacBio technology and Sanger sequences from 462 bacterial strains and 547 fungal strains (Supplementary Methods S2). These strains were all isolated from grapevine leaves collected from two of the plot pairs (Jaswa et al., in preparation) (Supplementary Methods S2). As a result, we obtained up to three taxonomic assignments for each ASV (Supplementary Methods S3). A decision tree was constructed to select the most reliable assignment (Supplementary Fig. S5).

Finally, we decontaminated the datasets using the *metabaR* R package v1.0.0 [75]. Since contamination may vary across experiments, depending on laboratory conditions and reagents, we performed separate decontamination of twelve subsets of the data, each corresponding to a combination of one barcode region (nrDNA ITS or 16S rRNA genes), one microbial habitat (topsoil, phyllosphere, or endosphere), and one sampling year (2022 or 2023). The *contaslayer* function was used to identify ASVs whose relative abundance in the entire dataset was highest in at least one control compared with true samples [75]. These ASVs were considered contaminants and were removed from the dataset. Next, the *tagjumpslayer* function was used to detect artifacts such as tag jumps and reduce the noise they cause by removing an ASV from a given PCR product if its relative abundance across the entire dataset was below a specified threshold. This threshold was determined empirically, as recommended in the metabaR tutorial,^1^ using the visualization functions of the *metabaR* package created for this purpose. Finally, a histogram of the number of reads per sample was plotted, and samples with low sequencing depth, located in the lower tail of the normal distribution curve, were considered failed PCRs and removed.

### Statistical analysis

All the statistical analyses were performed with R v4.2.3 [76]. Microbial community analyses were performed using the R packages *phyloseq* v1.48.0 [77] and *speedyseq* v0.5.3.9018 [78], and all figures were generated using the *ggplot2* v3 package. 5.1 [79], *cowplot* v1.1.3 [80], *ggh4x* v0.2.8 [81], ggsignif v0.6.4 [82], *patchwork* v1.2.0 [83], *microViz* v0.10.8 [84], and *ggtext* v0.1.2 [85]. Microbial community analyses were based on sample × ASV raw count matrices or matrices transformed to account for compositional effects. These effects were accounted for by transforming raw sequence counts using the centered log-ratio (CLR) transformation [86]. Prior to the CLR transformation, we applied a Bayesian multiplicative treatment of zeros in the sample × ASV matrices using the *cmultRepl* function of the *zComposition* package v1.5.0.3 [87]. This function converts zero counts, which would lead to errors in the log ratios, into estimates close to zero, assuming that these zeros are due to undersampling rather than absence. It also drops rows (ASVs) or columns (samples) with more than 80% zero or missing data. The analyses of beta and alpha diversity and the search for microbial consortia of interest for downy mildew biocontrol presented below were performed separately for each combination of microbial habitat (topsoil, phyllosphere, or leaf endosphere) and PCR marker (nrDNA ITS or 16S rRNA genes).

### Analysis of the relationship between foliar symptoms of downy mildew and the amount of inoculum in topsoil

We evaluated the relationship between the incidence and severity of downy mildew in the plot, which was defined based on several years of foliar and bunch symptom monitoring, and the concentration of *P. viticola* DNA in the topsoil at the time of sampling, using a linear mixed effects model. The model included the concentration of *P. viticola* DNA in the topsoil as the dependent variable and the the incidence and severity of downy mildew in the plot (high or low) as a fixed factor. Other variables that may influence the amount of *P. viticola* oospores in the topsoil were also included as fixed factors: the year of sampling (2022 or 2023), whether fungicide treatments were applied to the area sampled the year before (yes or no), and the location of the soil sample within the plot (on the edge of the plot or not). An interaction term between fungicide application and within-plot location was also included, and the plot pair was included as a random effect. Models were built using the *lmer* function from v3.1.3 of the *lmerTest* package [88] and evaluated with Type II ANOVA using v3.1.2 of the *car* package [89]. Graphical checks for homoscedasticity and normality of the residuals were performed using the packages *performance* v0.12.0 [90] and *DHARMa* v0.4.6 [91]. The variance explained by the model was estimated using the conditional coefficient of determination (R^2^c) and the marginal coefficient of determination (R^2^m) provided by the *r2* function of the *performance* package. The former coefficient represents the variance explained by the entire model, whereas the latter represents the variance explained by the fixed effects only.

### Analysis of the environmental factors driving within-plot and between-plot variation in the microbial composition of topsoil and leaf (endosphere and phyllosphere)

Principal Component Analysis (PCA) was applied to the sample × ASV matrix transformed by CLR to visualize variation in microbial community composition using the *microViz* package. We then identified key factors influencing community composition using variance partitioning and Redundancy Analysis (RDA), implemented using respectively the *varpart* and *rda* functions of the *vegan* package v2.6.4 [92]. The same explanatory factors were used in both analyses (Supplementary Table S1). For variance partitioning, we categorized them into 4 groups: soil physical chemistry (Soil; 9 variables), grape variety and management (Management; 3 variables), weather during the month before sampling (Weather; 30 variables), and sampling location (Space; 4 variables) (Supplementary Table S1). For both variance partitioning and RDA, the sample x ASV matrix transformed by CLR was used as the response. Non-numeric variables were treated as dummy variables, and all variables were standardized using the scale function. For the RDA, an automatic stepwise selection of explanatory variables, both forward and backward, was performed using the *ordistep* function of the *vegan* package in R. Finally, an RDA was performed with all selected explanatory variables included as constraints. Permutation tests were performed using the *anova.cca* function of the *vegan* package to assess the significance of the fitted models and to evaluate the marginal effects of the constraints.

### Testing whether microbial diversity in topsoil and leaves is higher in vineyard plots with lower downy mildew incidence and severity

To investigate whether microbial diversity in topsoil and leaves is higher in vineyard plots with lower downy mildew incidence and severity (hypothesis (i)), we calculated three α diversity indices. These indices are part of the Hill number framework [93], which includes a parameter *q* that determines the sensitivity of the indices to the relative abundance of ASVs. This framework gives less weight to rare ASVs as q increases. The Hill number corresponding to *q* = 0 represents the richness of ASVs, where each ASV counts is assigned a value of 1 regardless of its relative abundance. The Hill number corresponding to *q* = 1 is the exponential of Shannon's entropy index [94], where the weight of each ASV is proportional to its relative abundance. The Hill number corresponding to *q* = 2 is the inverse of Simpson's concentration index [95], which disproportionately favors abundant ASVs and is particularly relevant for metabarcoding data, as rare ASVs often correspond to artifacts, and their inclusion can lead to erroneous ecological conclusions [60]. The three observed alpha diversity indices, with *q* = 0 to 2, were calculated from the sample × ASV raw count matrix using the *ChaoRichness*, *ChaoShannon*, and *ChaoSimpson* functions in the *iNEXT* v3.0.1 package [93, 96]. The effect of downy mildew incidence and severity on microbial community alpha diversity was assessed using linear mixed effects models. We constructed 36 models, each corresponding to a combination of one microbial habitat (topsoil, phyllosphere or leaf endosphere), one PCR marker (nrDNA ITS or 16S rRNA genes), one year of sampling (2022 or 2023) and one alpha diversity index (*q* = 1, 2 or 3). For the 2022 sampling campaign, the model included three fixed effects: the incidence and severity of downy mildew in the plot (high or low), whether fungicides were applied to the sampled area in the year before (yes or not), and the location of the sample within the plot (on the edge or not). For the 2023 sampling campaign, the model included only the incidence and severity of downy mildew in the plot (high or low) because the samples were collected exclusively from the center of the plots, and all the sampled vines had received fungicide treatment the year before. Both models included pairs of plots as random effects. Graphical checks for homoscedasticity and normality of the residuals were performed using the *performance* and *DHARMa* packages. Model construction and evaluation were performed using the *lmerTest* and *car* packages.

### Testing whether plots with low downy mildew incidence and severity harbor a greater abundance of specific microbial taxa in topsoil and leaves

To investigate whether plots with low downy mildew incidence and severity harbored a higher abundance of specific microbial taxa (hypothesis (ii)), we used a set of four Differential Abundance Analysis (DAA) methods recommended in the recent literature: ANCOM-BC2 [97], Maaslin2 [98], LinDA [99] and ZicoSeq [100]. We selected these methods for the following reasons: they were specifically developed for microbiota analysis by explicitly accounting for zero inflation and compositional effects; they allow the specification of random and covariate effects; and they have been recommended in recent methodological studies [100–103]. This set of 4 DAA was used to compare ASV abundances between plots with high and low downy mildew incidence and severity while accounting for microbial community variation between plot pairs. For all four methods, the plot pair was included as a random factor, and the parameters were set to defaults except for the minimum prevalence threshold, which was set to 10%, and the adjusted p value for an ASV to be considered differentially abundant, which was set to 0.05. All analyses were performed using the sample × ASV raw count matrix. They were performed using the *ANCOMBC* v2.0.3 [104, 105], *Maaslin2* v1.12.0 [98], *GUniFrac* v1.8 [106], and *MicrobiomeStat* v1.2 [107] packages.

For each ASV identified as differentially abundant by at least one of the four DAA methods, we calculated two scores: (1) the number of methods that identified the ASV as differentially abundant, ranging from 1 to 5, and (2) the average association coefficient between the methods. To calculate the average association coefficient, the coefficients provided by each DAA method were standardized between 0 and 1 if the ASV was more abundant in low incidence and severity plots and between 0 and -1 if the ASV was more abundant in high incidence and severity plots before the average coefficient between methods was calculated. Finally, we included the Random Forest (RF) algorithm (see below) as a fifth method and used the Gini index provided by the RF algorithm to calculate the average association coefficient.

### Testing whether plots with low amount of downy mildew inoculum harbor a greater abundance of specific microbial taxa in topsoil and leaves

In addition, we investigated whether plots with low amounts of *P. viticola* oospores in the topsoil harbored specific microbial taxa using the Threshold Indicator Taxa ANalysis (TITAN) method of the *TITAN2* package v2.4.3 [108]. This method analyzes changes in community composition along ecological gradients. Our goal was to identify microbial taxa that covary with the concentration of *P. viticola* DNA in the topsoil. For this analysis, we used the sample × ASV raw count matrix, retaining only microbial ASVs with more than 100 total reads and present in at least 3 samples, as required by the *titan* function. The method identified ASVs whose abundance increased as the *P. viticola* DNA concentration decreased and ASVs whose abundance increased as the *P. viticola* DNA concentration increased. These ASVs are hereafter referred to as indicators of low and high *P. viticola* DNA concentrations in topsoil. To evaluate the strength of the relationship, we used the standardized Indicator Value (IndVal) score defined by Dufrene and Legendre [109] and expressed it as a z score.

### Testing whether the incidence and severity of downy mildew in the plot are predictable from microbiota composition

We used Random Forest (RF) algorithms to assess the predictive power of microbiota composition. We tested whether downy mildew incidence and severity in plots can be predicted from the microbiota composition (hypothesis (iii)) and whether the leaf microbiota is a better predictor of disease severity and incidence compared to the soil microbiota (hypothesis (iv)). For this purpose, we used the *microranger* package v0.0.0.9000 [110, 111], which includes RF classification functions derived from the *ranger* package [112] and was specifically designed to classify microbial communities.

We first analyzed the predictive power of microbiota composition on samples collected within the same year, using data collected in 2022. We used the *rf.opti.mtry.taxo* function to train the RF algorithm on a set of 36 ASV tables for each microbial habitat (phyllosphere, leaf endosphere, and topsoil). Each table was a sample × ASV raw count matrix corresponding to a microbiota subset (fungi only, bacteria only, or both), a filtering threshold (all ASVs or only abundant ASVs), and a level of taxonomic aggregation (ASV, species, genus, family, class, or order). Abundant ASVs were defined as those with a read count greater than the third quartile of the ASV read count distribution. Training of the RF algorithm involved 500 decision trees for each table, with nonrandom k-fold cross-validation. The algorithm used 80% of the pairs of plots as the training dataset and the remaining 20% as the validation dataset. The cross-validation was performed on all possible splits of the entire dataset, with the only condition that the plots belonging to the same pair were both in the training dataset or both in the validation dataset. This condition ensured that the algorithm focused on learning downy mildew incidence and severity rather than the spatial location of the plots. We optimized each algorithm using 20 different values for the mtry parameter, which represents the number of variables considered at each node division of the decision tree. The best mtry value for each of the 36 ASV tables was selected on the basis of the error rate of the algorithm, as done in Cambon et al. [111]. Next, we used the *rf.blind* function to obtain the Gini index and associated *p* value estimated by permutation using the Altmann method [113], allowing us to identify which ASVs were most important for classification.

Finally, we assessed the predictive power of the microbiome composition on the samples collected the following year. The 2022 dataset was used for training, and the 2023 dataset for validation. To create the validation sets, we randomly grouped pairs of plots sampled in 2023 into groups of two, with each plot assigned to a single validation group. We then used all possible combinations of 80% of the plot pairs sampled in 2022 for training and validated the model on each validation group.

## Results

### Seven pairs of vineyard plots were selected based on epidemiological records of downy mildew

**Fig. 1:**
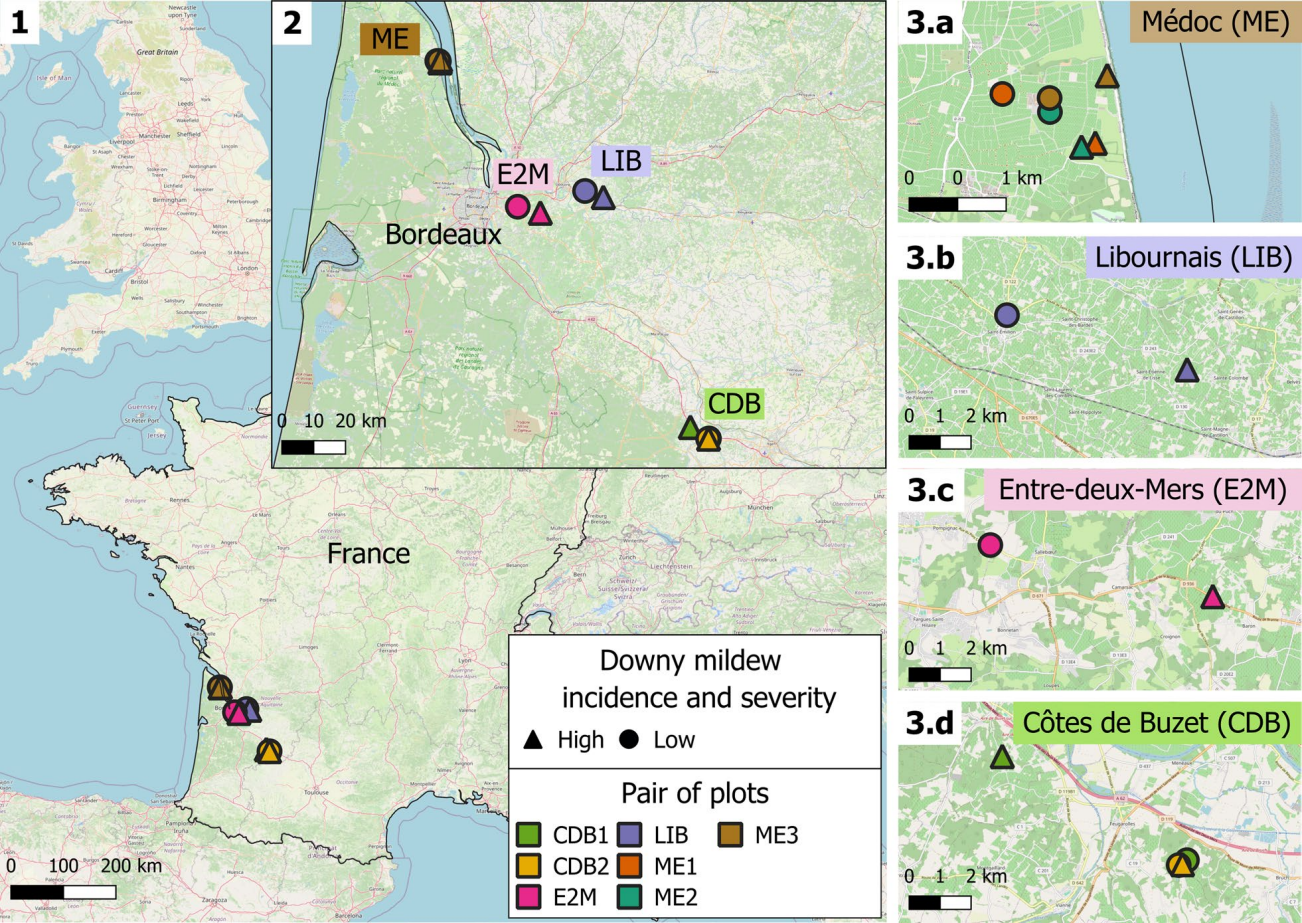
Map of the vineyard plots. Seven pairs of plots that differed in their downy mildew incidence and severity were selected for the present study. They were all located in France (map 1) in the Nouvelle-Aquitaine region (map 2). They belonged to four wine-growing areas, namely, Médoc (ME), Libournais (LIB), Entre-deux-Mers (E2M) and Côtes de Buzet (CDB) (maps 3a–d). Plots belonging to the same pair are represented with the same color, with symbols indicating high and low downy mildew incidence and severity (triangles and circles, respectively). The distance between plots within a pair ranged from 0 km for pair CDB2 (adjacent plots) to 7.5 km for pair E2M (Table 1)

**Fig. 2:**
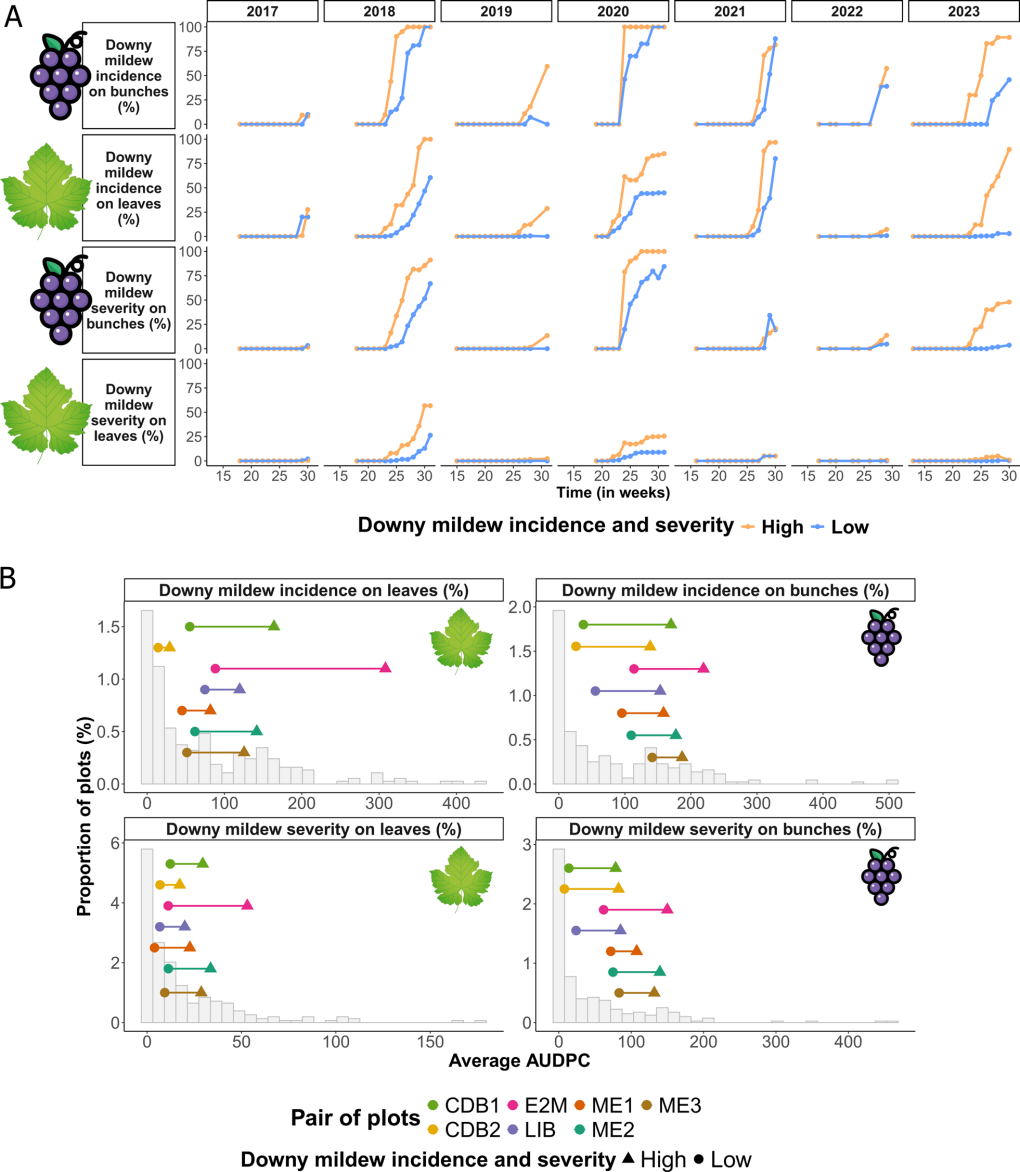
Epidemiological features of vineyard plots**.** **A** Downy mildew disease progression curves for the high- incidence and severity (in orange) and low- incidence and severity (in blue) plots of the ME2 plot pair over seven years of epidemiological monitoring and **B** average areas under the downy mildew progression curves (AUDPC) for the seven pairs of plots selected in the present study. AUDPC values were averaged across the years 2019, 2020, and 2021 (i.e., the common years of epidemiological monitoring for the seven pairs of plots). The average AUDPC values for plots belonging to the same pair are represented with the same color, with symbols indicating high or low downy mildew incidence and severity (triangles and circles, respectively). The gray histograms represent the distribution of average AUDPC values for the other plots included in the epidemiological database and monitored during the same years

Seven different pairs of vineyard plots, all located in southwestern France (Fig. 1), were selected for the present study based on the analysis of epidemiological records (Table 1). They came from four distinct wine-growing regions and were named according to their region of origin: three in the Médoc (ME1, ME2, ME3), one in Libournais (LIB), one in Entre-deux-Mers (E2M) and two in Côtes de Buzet (CDB1, CDB2) (Fig. 1 and Table 1). Both plots within a pair were planted with the same variety, managed in a similar way and located close to each other (Table 1). Each pair of plots consisted of a plot with lower downy mildew incidence and severity and a plot with higher downy mildew incidence and severity (Fig. 2A and Supplementary Fig. S6). The area under the disease progression curve (AUDPC) was calculated for the years 2019–2021 and averaged for each of the 7 pairs and compared with that of the 1200 plots monitored by IFV (Fig. 2B).

**Table 1:** Description of the pairs of vineyard plots

Plot pair	Plot name	Downy mildew incidence and severity	GPS coordinates	Geographic distance between plots	Grape variety	Management	Sampling year(s)
Libournais (LIB)	LIB_L	Low	44° 53′ 52.4″ N 0° 09′ 13.7″ W	6 km	Merlot Noir	Biodynamic	2022 and 2023
	LIB_H	High	44° 52′ 55.6″ N 0° 04′ 48.3″ W				
Médoc1 (ME1)	ME1_L	Low	45° 14′ 50.7″ N 0° 46′ 00.4″ W	1 km	Merlot Noir	Organic	2022 and 2023
	ME1_H	High	45° 14′ 35.8″ N 0° 45′ 15.4″ W				
Médoc2 (ME2)	ME2_L	Low	45° 14′ 45.4″ N 0° 45′ 37.6″ W	0.5 km	Cabernet Sauvignon	Organic	2022 and 2023
	ME2_H	High	45° 14′ 34.3″ N 0° 45′ 21.8″ W				
Médoc3 (ME3)	ME3_L	Low	45° 14′ 50.1″ N 0° 45′ 38.0″ W	0.6 km	Merlot Noir	Organic	2023
	ME3_H	High	45° 14′ 58.5″ N 0° 45′ 11.3″ W				
Entre-deux-Mers (E2M)	E2M_L	Low	44° 50′ 43.8″ N 0° 25′ 16.3″ W	7.5 km	Merlot Noir	Conventional	2022 and 2023
	E2M_H	High	44° 49′ 52.1″ N 0° 19′ 42.2″ W				
Côtes de Buzet1 (CDB1)	CDB1_L	Low	44° 12′ 38.5″ N 0° 22′ 21.7″ E	6.7 km	Merlot Noir	Conventional	2022
	CDB1_H	High	44° 14′ 26.7″ N 0° 17′ 53.0″ E				
Côtes de Buzet2 (CDB2)	CDB2_L	Low	44° 12′ 33.4″ N 0° 22′ 09.7″ E	0 km	Merlot Noir	Organic	2023
	CDB2_H	High	44° 12′ 34.0″ N 0° 22′ 15.3″ E				

Each pair consisted of one plot with low downy mildew incidence and severity (labeled_L) and one plot with high downy mildew incidence and severity (labeled_H) according to long-term epidemiological data. Five pairs of plots were sampled in 2022, and six were sampled in 2023. All the grape varieties in these plots are naturally susceptible to downy mildew

The AUDPC values of the 7 plots with low downy mildew incidence and severity were always lower than those of the plots with high-incidence and severity for the 4 variables of the same pair, as expected by the selection process (Fig. 2B). The differences between low and high values of the same pair varied between pairs and between variables (Fig. 2B). For most pairs except CDB2, all AUDPC values in the low-incidence and severity plots are lower than the lowest AUDPC value in the high-incidence and severity plots. The values of the selected pairs are among the most frequent values of the 1200 plots but not the extremes (Fig. 2B).

The pairs of plots differed slightly between the 2022 and 2023 sampling campaigns (Table 1). In 2022, 14 vineyard plots, which formed 7 pairs that resulted contrasting in terms of epidemiological history, met the 5 selection criteria. However, only 5 pairs (named LIB, ME1, ME2, E2M, and CDB1) could be sampled at the chosen phenological stage and before the first fungicide treatment. In 2023, 12 vineyard plots, which formed 6 pairs contrasting in terms of epidemiological history, met the 5 selection criteria. These pairs included 4 pairs already selected in 2022 (all except CDB1, where one of the plots started an organic conversion) and two new pairs (ME3, CDB2) (Table 1). All six selected pairs could be sampled at the chosen phenological stage and before the first fungicide treatment. In total, we studied 7 pairs of plots (LIB, ME1, ME2, ME3, E2M, CDB1, and CDB2), and each pair was sampled at least once (Table 1).

### The amount of downy mildew inoculum in topsoil is lower in plots with low downy mildew incidence and severity

The topsoil *P. viticola* DNA concentration was significantly lower in plots classified as low- incidence and severity plots based on symptoms on leaves and bunches (Linear Mixed-Effects Models, *p* < 0.05*; Supplementary Table S2 and Fig. S7). In addition, topsoil samples collected from plot areas treated with fungicide in the previous year had significantly lower topsoil *P. viticola* DNA concentrations (Linear Mixed-Effects Models, *p* < 0.001***; Supplementary Table S2 and Fig. S7). Within-plot location had a significant effect on the topsoil *P. viticola* DNA concentration, with samples collected along plot edges showing a lower concentration than those collected in the center of the plots (Linear Mixed-Effects Models, *p* < 0.001***; Supplementary Table S2 and Fig. S7). In addition, we detected a higher concentration of *P. viticola* DNA in the soil in 2022 than in 2023 (Linear Mixed-Effects Models, *p* < 0.001***; Supplementary Table S2 and Fig. S7), which was likely due to the more severe downy mildew epidemic in 2021 than in 2022 (Fig. 2A and Supplementary Fig. S6).

### Topsoil, phyllosphere and leaf endosphere microbial communities have different compositions

After filtering, the final fungal dataset contained 136,681,217 reads grouped into 795 ASVs, while the final bacterial dataset contained 32,678,727 reads grouped into 1561 ASVs (Supplementary Table S3). Using PacBio sequencing, we obtained 35,934 reads representing 524 bacterial ASVs, allowing us to improve the taxonomic assignment of 154 bacterial ASVs (out of 1561). Using Sanger sequencing of our collection of grapevine foliar microorganisms, we were able to improve the taxonomic assignment of 88 fungal ASVs (out of 795).

**Fig. 3:**
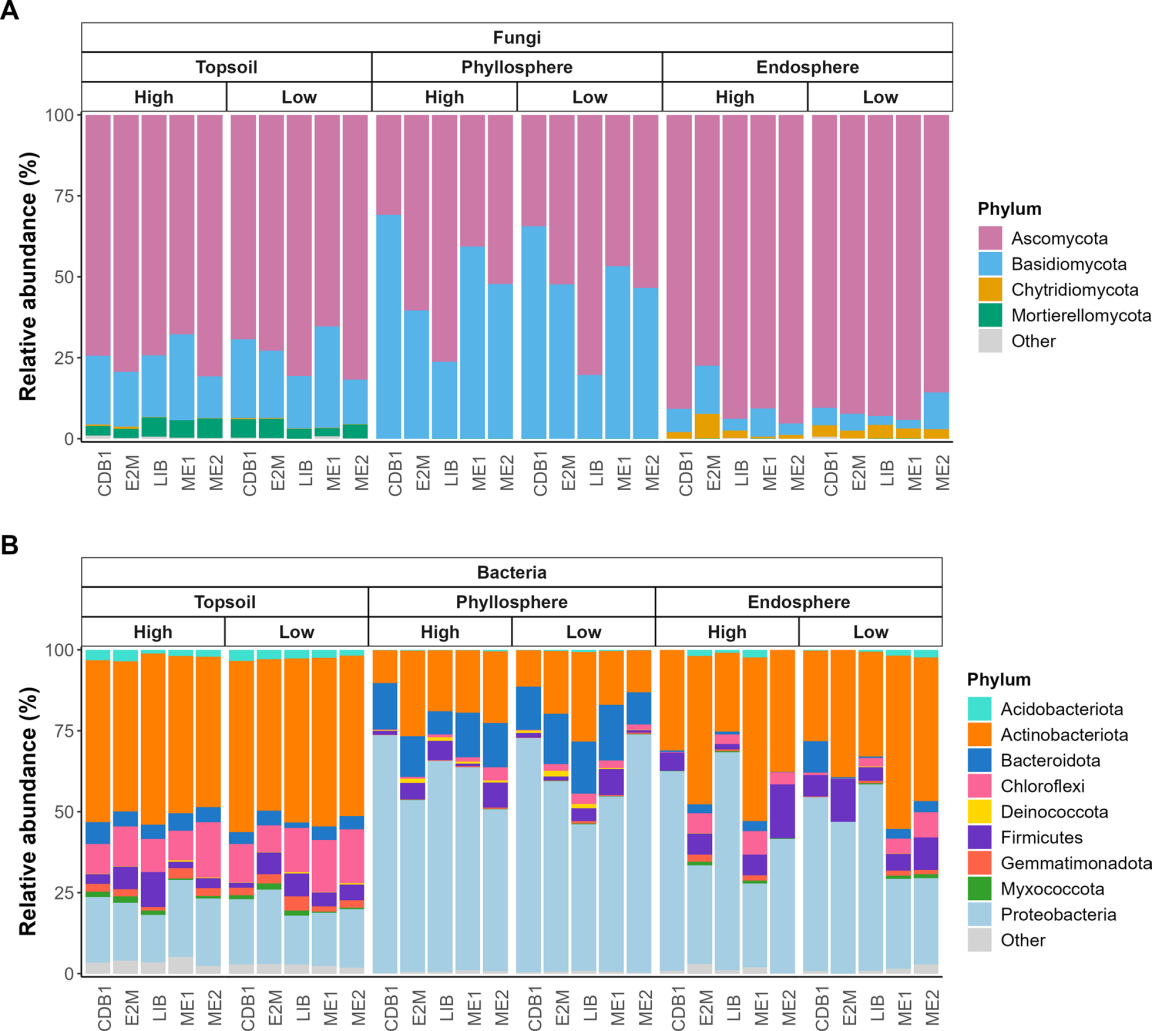
Microbial community profiles depending on vineyard plot downy mildew incidence and severity. **A** Fungal and **B** bacterial community profiles of the topsoil, phyllosphere and leaf endosphere, representing the relative abundance of the different phyla averaged over the sixteen samples collected in each plot and each microbial habitat during the 2022 sampling campaign. Phyla representing less than 1% of the sequences were grouped into the “Other” category

Topsoil, phyllosphere and leaf endosphere harbored different microbial community compositions (Fig. 3), including variations in the most abundant fungal species and bacterial genera (Supplementary Table S4 and S5). Microbial community profiles remained consistent across years (Fig. 3, Supplementary Fig. S8; Supplementary Tables S4–S7).

### Soil physico-chemistry and weather have a stronger influence on microbiota composition than grape variety and management practices

**Fig. 4:**
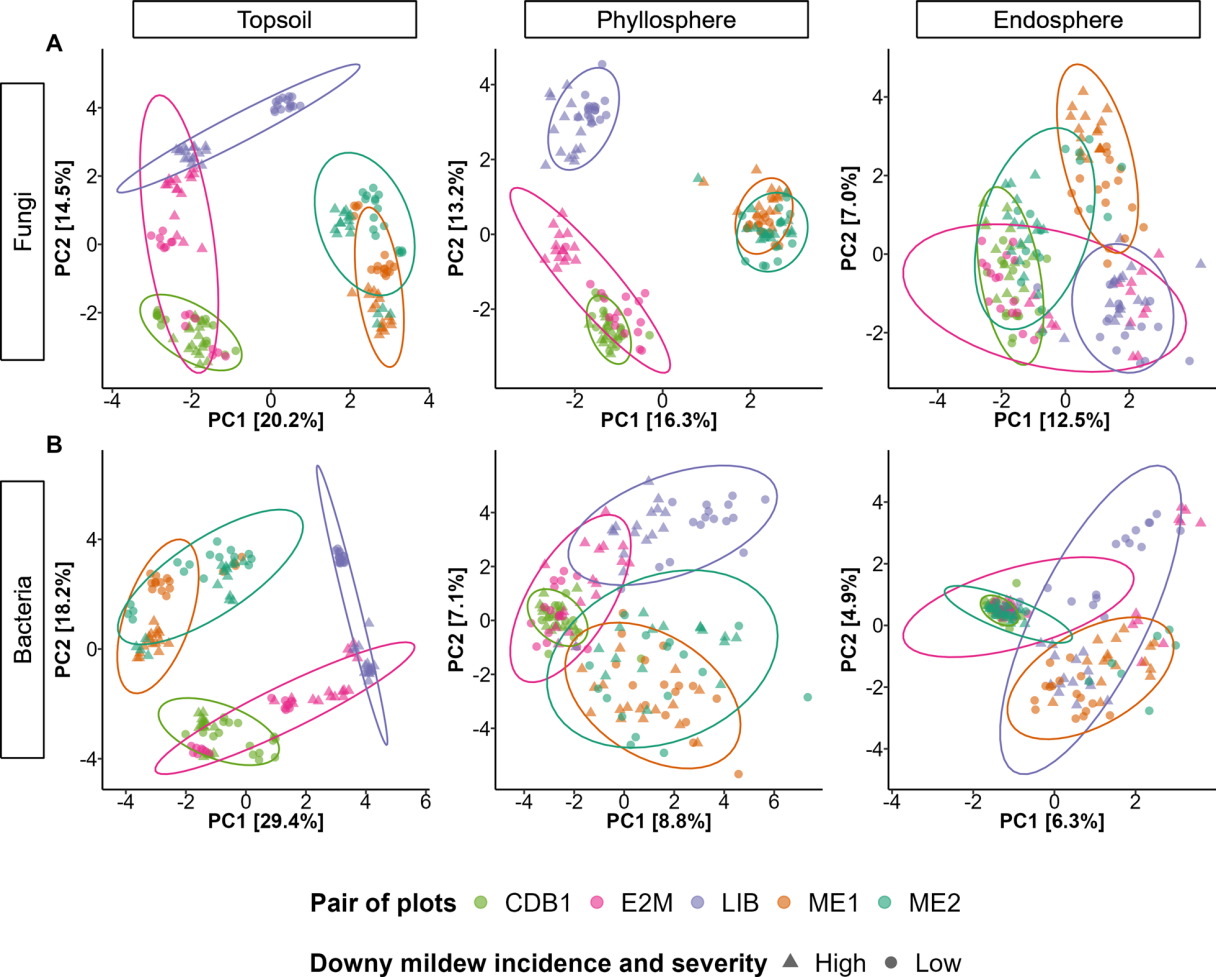
Variation in microbial community composition across vineyard plots. Dissimilarities among **A** fungal and **B** bacterial communities in the topsoil, phyllosphere and leaf endosphere for the five pairs of plots sampled in 2022, represented using a Principal Component Analysis (PCA). Samples collected from the same pair of plots are shown in the same color, with symbols indicating high and low downy mildew incidence and severity (triangles and circles, respectively)

Topsoil microbial communities (both fungi and bacteria) and phyllosphere fungal communities were spatially structured, with marked differences in composition between plots and plot pairs. This spatial structuring was consistent across years (Fig. 4 for 2022 and Supplementary Fig. S9 for 2023). Each plot pair had its own microbial community composition, and the plot pairs that were geographically close (for example, ME1 and ME2 in 2022, both located in the Medoc region; Fig. 1) had more similar communities (Fig. 4 and Supplementary Fig. S9). In contrast, the phyllosphere bacterial communities and leaf endosphere communities (both fungi and bacteria) showed little spatial structuring (Fig. 4 for 2022 and Supplementary Fig. S9 for 2023).

The composition of the topsoil fungal and bacterial communities, as well as the phyllosphere fungal communities, were mainly structured by two factors: the weather of the past month (*Weather*) and the physicochemical properties of the soil (*Soil*) (Supplementary Table S1). In 2022, *Weather* explained 43.01% of the variance in topsoil fungal community composition, 53.56% of the variance in topsoil bacterial community composition, and 33.75% of the variance in phyllosphere fungal communities (Supplementary Fig. S10). *Soil* was nearly as influential, accounting for 42.08% of the variance in topsoil fungal community composition, 54.76% of the variance in topsoil bacterial community composition, and 29.77% of the variance in phyllosphere fungal communities (Supplementary Fig. S10). In 2023, the ranking of environmental drivers remained similar, with *Weather* and *Soil* being the primary drivers of topsoil fungal and bacterial communities and phyllosphere fungal communities (Supplementary Fig. S11 and Supplementary file S2).

*Management* factors, including grape variety, management type (organic, biodynamic, conventional), and whether fungicide treatments were applied the year before sampling, had less influence on the microbiota composition than *Weather* and *Soil* (Supplementary Fig. S10) but were still retained as significant variables (Supplementary file S2). For example, management type, grape variety and fungicide treatment applied the year before sampling significantly influenced the composition of phyllosphere fungal communities (Supplementary file S2).

### Fungicide treatments decrease microbiota diversity in both topsoil and grapevine leaves, whereas edge effects increase it

Fungicide treatments altered not only the composition of phyllosphere fungal communities (Supplementary file S2) but also their diversity (Supplementary Tables S8 and S9). Using the 2022 dataset, we showed that fungicide treatments applied the year before sampling decreased the diversity of fungal communities in both the phyllosphere and topsoil (Supplementary Table S8). In contrast, edge effects increased microbiota diversity. Topsoil and endosphere fungal and bacterial communities, were more diverse at the edge of the plot than at the center (Supplementary Table S8). These analyses could not be applied to the 2023 data because samples were collected exclusively from the center of the plots that year, and all the samples originated from grapes that had received fungicide treatment the previous year.

### Microbial diversity is not consistently higher in plots with lower downy mildew incidence and severity

According to hypothesis (i), phyllosphere and endosphere fungal communities sampled in 2022 were more diverse in plots with low downy mildew incidence and severity than in plots with high downy mildew incidence and severity (Supplementary Table S8 and Fig. S12). Differences were significant when diversity was measured by Shannon's index but not Simpson's index or observed richness (Supplementary Table S8 and Fig. S12). However, these results were not consistent across years. In 2023, there was no difference in phyllosphere fungal diversity between plots with low and high downy mildew incidence and severity (Supplementary Table S9 and Fig. S13). In addition, the diversity of endophytic fungi showed the opposite pattern from that in 2023 (Supplementary Tables S8 and S9 and Fig. S12 and S13). Similarly, the difference in topsoil microbiota diversity between plots with low and high downy mildew incidence and severity was not consistent across years. In 2022, both topsoil fungal and bacterial communities were more diverse in plots with high downy mildew incidence and severity, contrary to hypothesis (i), while these differences were not significant in 2023 (Supplementary Tables S8 and S9 and Fig. S12 and S13).

### Plots with low downy mildew incidence and severity harbor a greater abundance of specific microbial taxa

**Fig. 5:**
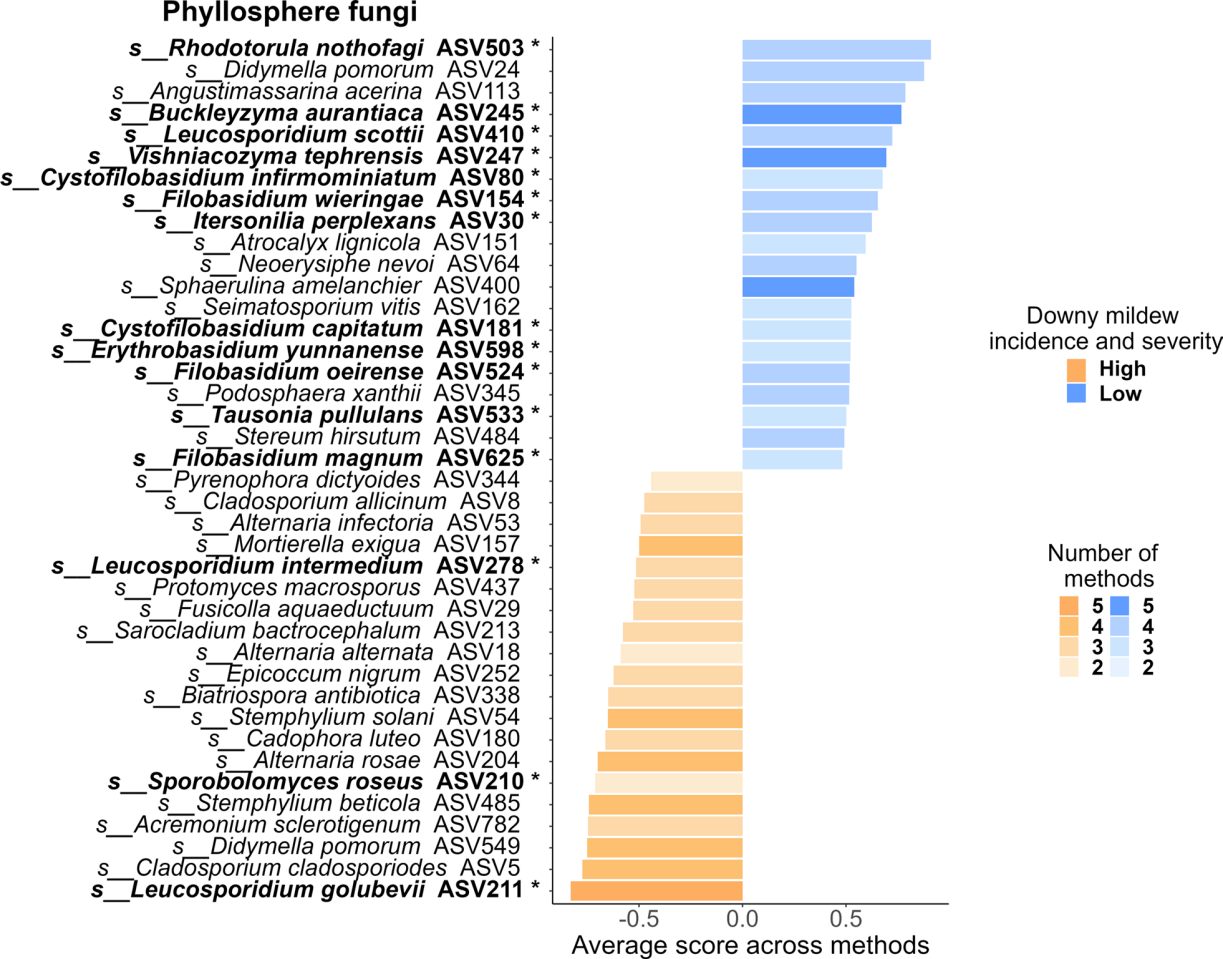
Phyllosphere fungal taxa that vary in abundance with downy mildew incidence and severity. For each condition (high vs. low incidence and severity), we represented the 20 Amplicon Sequence Variants (ASVs) that were significantly associated with the condition according to at least two methods, that could be assigned at the species level, and that had the highest average association scores. The five methods used to identify these ASVs were ANCOM-BC2 [97], Maaslin2 [98], LinDA [99], ZicoSeq [100] and Random Forest Classification [111]. The results are based on data collected in 2022. ASVs belonging to Basidiomycete yeasts are highlighted in bold and marked with an asterisk (*)

Consistent with hypothesis (ii), our analyses of the 2022 dataset revealed 241 fungal and 462 bacterial ASVs that were significantly more abundant in plots with low downy mildew incidence and severity (Supplementary file S3). Differentially abundant ASVs were mainly found in the topsoil (141 fungal and 453 bacterial ASVs) and, to a lesser extent, in the phyllosphere (128 fungal and 70 bacterial ASVs). Only 11 differentially abundant taxa, all fungi, were detected in the leaf endosphere (Supplementary Fig. S14). In the topsoil, fungal species such as *Coniochaeta fasciculata*, *Fusarium brachygibbosum*, *Didymella pomorum*, *Volutella ciliata*, *Robillarda sessilis*, *Paraphoma pye*, and the *Trichoderma* species (*T. virens* and *T. lixii*) scored the highest (Supplementary Fig. S15). Additionally, the genus *Mortierella* (including *M. exigua*, *M. alpina*, and *M. elongata*) and basidiomycetous yeasts, such as *Papiliotrema* (including *P. laurentii*, *P. terrestris*, and *P. flavescens*), *Rhodotorula* (including *R. babjevae* and *R. nothofagi*), *Leucosporidium* (*L. scottii* and *L. fragarium*), *Cystofilobasidium infirmominiatum*, *Erythrobasidium yunnanense*, *Filobasidium magnum*, *Filobasidium oeirense*, and *Dioszegia hungarica* were also well-represented (Supplementary Fig. S15 and Supplementary file S3). In the phyllosphere, a large proportion of the taxa that were significantly more abundant in plots with low downy mildew incidence and severity were basidiomycete yeasts, such as *E. yunnanense*, *Tausonia pullulans*, *C. infirmominiatum*, *C. capitatum*, *R. nothofagi*, *Vishniacozyma tephrensis*, *Buckleyzyma aurantiaca*, *Itersonilia perplexans*, *F. magnum*, *F. wieringae*, and *F. oeirense* (Fig. 5). *B. aurantiaca*, *V. tephrensis* and *Sphaerulina amelanchier* were consistently associated with low downy mildew incidence and severity across all five DAA methods (Fig. 5). Conversely, some fungal ASVs were significantly more abundant in high-incidence and severity plots, such as the grapevine foliar pathogen *Botrytis cinerea*, which was more abundant in both the topsoil and phyllosphere (Supplementary file S3).

**Fig. 6:**
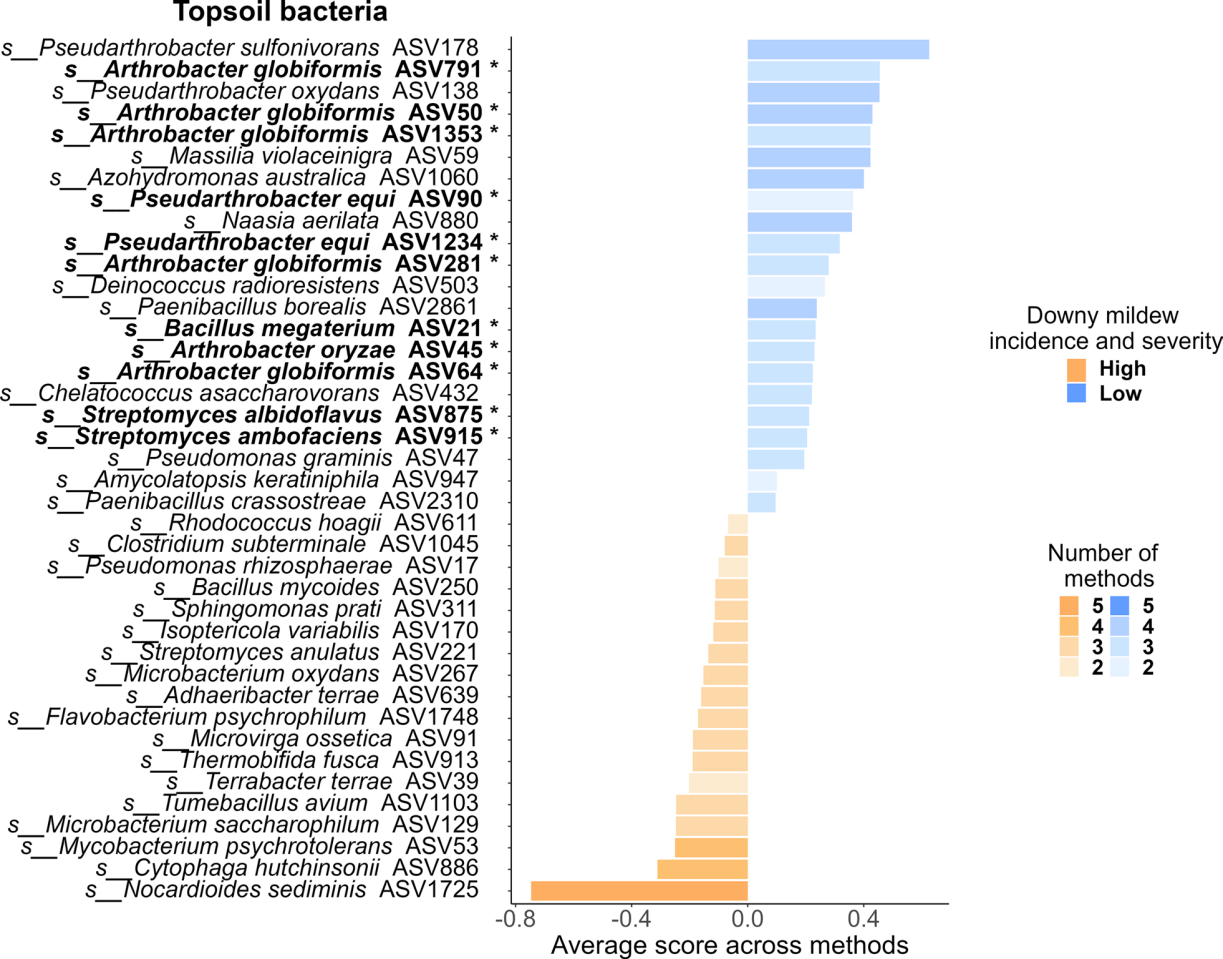
Topsoil bacterial taxa that vary in abundance with downy mildew incidence and severity. For each condition (high vs. low incidence and severity), we represented the 20 Amplicon Sequence Variants (ASVs) that were significantly associated with the condition according to at least two methods, that could be assigned at the species level, and that had the highest average association scores. The five methods used to identify these ASVs were ANCOM-BC2 [97], Maaslin2 [98], LinDA [99], ZicoSeq [100] and Random Forest Classification [111]. The results are based on data collected in 2022. ASVs highlighted in bold and marked with an asterisk (*) also scored highest in the TITAN [108] analysis

The most represented bacterial genera among the ASV*s* that could be assigned to species level were *Pseudarthrobacter*, including *P. equi* (represented by 2 different *ASVs*), *P. oxydans*, and *P. sulfonivorans*; *Pseudomonas*, such as *P. graminis* and *P. viridiflava*; *Streptomyces*, which includes *S. albidoflavus*, *S. ambofaciens*, *S. hygroscopicus*, and *S. xinghaiensis*; *Massilia*, encompassing *M. violaceinigra*, *M. putida*, and *M. aurea*; *Bacillus*, comprising *B. nealsonii*, *B. litoralis*, *B. megaterium*, and *B. thermolactis*; and *Sphingomonas*, featuring *S. astaxanthinifaciens*, *S. aurantiaca*, and *S. sediminicola* (represented by 2 different *ASVs*) (Fig. 6 and Supplementary file S3). In addition, five ASVs of *Arthrobacter globiformis* were also found to be significant (Fig. 6 and Supplementary file S3). In the phyllosphere, two bacterial ASVs that could be assigned to species level were more abundant in low- incidence and severity plots as determined by at least two methods: *Arthrobacter pascens* and *Corynebacterium stationis* (Supplementary Fig. S16), among others that could not be assigned to species.

Similar analyses were performed with the 2023 dataset, which was smaller than the 2022 dataset because only 4 samples per plot were collected in 2023, whereas 16 samples per plot were collected in 2022. Using this smaller dataset, we identified 75 fungal and 374 bacterial ASVs that were significantly more abundant in plots with low downy mildew incidence and severity (Supplementary file S3). Most of the differentially abundant ASVs were found in the topsoil (73 fungal ASVs and 373 bacterial ASVs). More than half of these ASVs (40 fungal ASVs and 227 bacterial ASVs) were also found to be differentially abundant in the 2022 dataset, indicating some reproducibility of the results across years. In addition, four differentially abundant ASVs (2 fungal ASVs and 2 bacterial ASVs) were detected in the phyllosphere in the 2023 dataset, one of which (*Pseudopeziza medicaginis* ASV720) had already been identified as differentially abundant in the 2022 dataset (Supplementary file S3).

### Areas with low amounts of downy mildew primary inoculum in the topsoil harbor a greater abundance of specific microbial taxa

Consistent with hypothesis (ii), our analyses of the 2022 data identified 205 fungal ASVs and 518 bacterial ASVs whose relative abundances changed significantly with *P. viticola* DNA concentration in the topsoil (Supplementary file S4). Among them, 94 fungal ASVs (57 in the topsoil, 37 in the phyllosphere, and 7 in the endosphere) and 387 bacterial ASVs (196 in the topsoil, 272 in the phyllosphere, and none in the endosphere) were indicators of low DNA concentrations of *P. viticola* (Supplementary file S4). More than half of these indicators of low amounts of *P. viticola* primary inoculum in the topsoil were identified as more abundant in plots classified as low downy mildew incidence and severity based on symptoms observed on leaves and bunches (Supplementary file S3–S4). Among the fungal ASVs most strongly associated with low levels of *P. viticola* primary inoculum, we detected *Trichoderma hamatum, R. babjevae* in the topsoil, *Bullera alba, A. pullulans, C. infirmominiatum, Cystofilobasidium macerans* in the phyllosphere, and *Vishniacozyma dimennae* in the endosphere (Supplementary Table S10 and Supplementary file S4). The most represented bacterial genera among the ASVs that could be assigned to the species level were *Bacillus*, *Sphingomonas*, *Pseudarthrobacter*, *Pseudomonas*, *Methylobacterium*, and *Streptomyces* (Supplementary file S4). *S. albidoflavus*, *S. ambofaciens*, *S. oralis*, *B. megaterium*, *A. globiformis* and *P. equi* scored the highest in topsoil and phyllosphere (Table 2 and Supplementary Table S11).

**Table 2:** Bacterial taxa indicators of low downy mildew primary inoculum in topsoil

Bacterial ASV	TITAN z score	Relative abundance (%)	Prevalence (%)
*Exiguobacterium sibiricum* ASV169	7.08	0.01	26.88
*Streptomyces ambofaciens* ASV915	6.73	0.02	69.38
*Bacillus megaterium* ASV21	6.72	1.23	92.50
*Streptomyces albidoflavus* ASV875	6.05	0.02	66.25
*Arthrobacter globiformis* ASV281	5.69	0.16	80.63
*Arthrobacter globiformis* ASV64	5.57	0.62	81.88
*Pseudarthrobacter equi* ASV1234	5.29	0.02	50.63
*Arthrobacter globiformis* ASV791	5.19	0.07	10.00
*Arthrobacter globiformis* ASV1353	5.11	0.03	10.00
*Arthrobacter oryzae* ASV45	4.97	0.89	91.88

These ten bacterial ASVs increase significantly in abundance as the concentration of *P. viticola* DNA in the topsoil decreases, according to the Threshold Indicator Taxa Analysis (TITAN) method [108], and they have the highest z scores. These results are based on the 2022 topsoil data. Relative abundance (%) indicates the proportion of sequences assigned to the taxa relative to the total number of sequences in the dataset. Prevalence (%) indicates the percentage of samples where the taxa are present with at least one sequence. ASVs highlighted in bold also scored highest in the Differential Abundance Analysis

Similarly, analysis of the smaller dataset collected in 2023 revealed 27 fungal ASVs (22 in the topsoil, 5 in the phyllosphere and none in the endosphere) and 68 bacterial ASVs (57 in the topsoil, 12 in the phyllosphere and none in the endosphere) that were indicators of low *P. viticola* DNA concentrations in the topsoil (Supplementary file S4). Among these, 9 fungal ASVs and 26 bacterial ASVs were also identified as indicators using the data from 2022 (Supplementary file S4).

### Vineyard downy mildew incidence and severity can be predicted from microbiota composition, with soil fungal communities being the best predictor

**Fig. 7:**
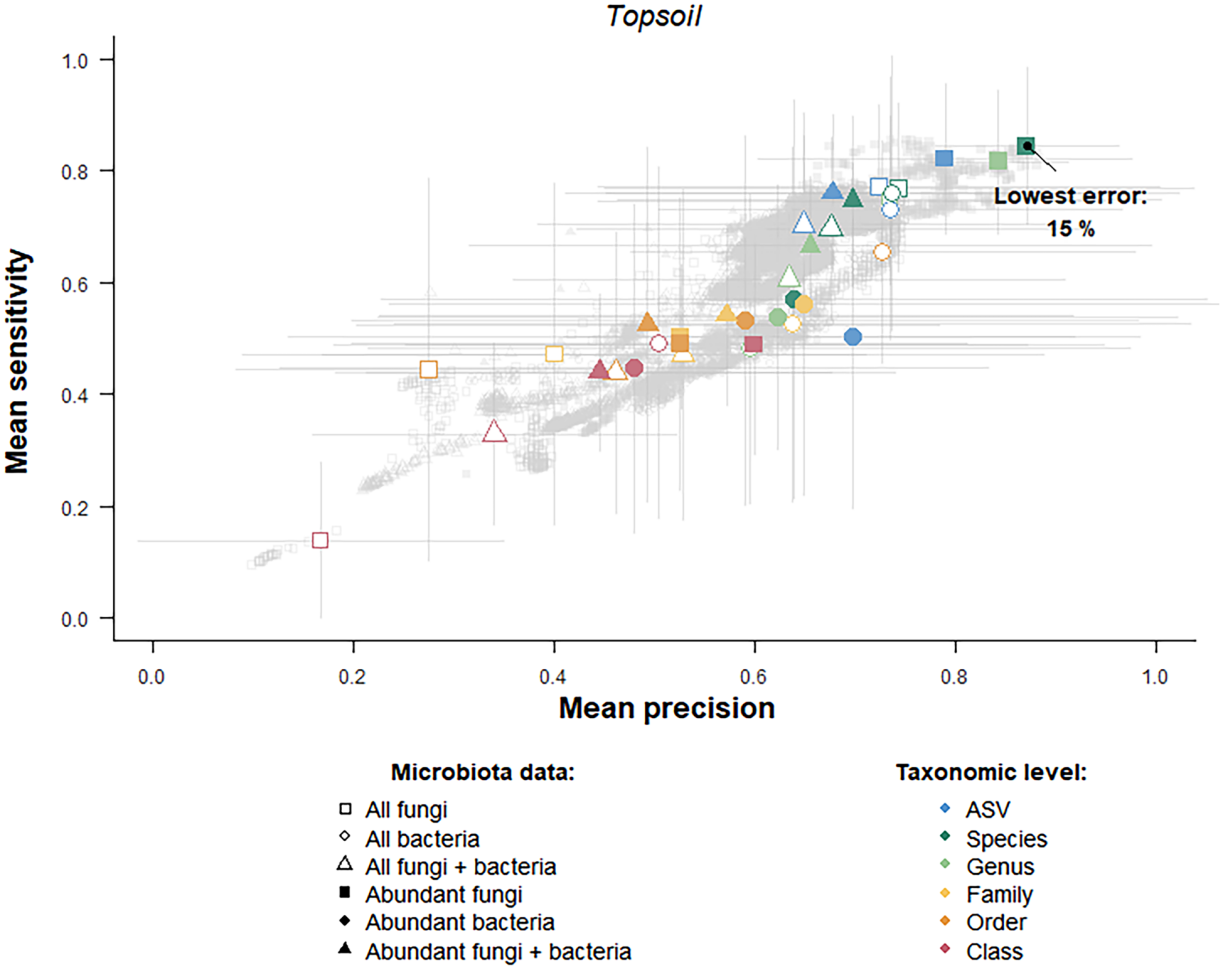
Random Forest algorithm performance in predicting grapevine downy mildew incidence and severity using topsoil microbiota composition. Each dot represents the mean sensitivity and precision obtained for a given subset of the topsoil microbiota data collected in 2022. The subsets differ in their composition (all fungi, all bacteria, all taxa or only abundant ones) and their level of taxonomic aggregation (from no aggregation of the ASVs to aggregation to the class level). The colored dots are those obtained with the optimal mtry value (i.e., the value that predicts low downy mildew incidence and severity with the lowest error rate). The bars represent the standard deviation over the various iterations of the cross-validation step. The lowest error rate is indicated as a percentage. The analysis and figure use the scripts developed by Cambon et al. [111]

According to hypothesis (iii), downy mildew incidence and severity could be predicted from microbiota data. The RF algorithm trained with topsoil samples collected in 2022 successfully predicted downy mildew incidence and severity for other topsoil samples collected in the same year. The lowest error rate was 15% ± 10. This was achieved using a data subset that included abundant fungal taxa aggregated to the species level (Fig. 7). The optimal mean mtry value was 35, indicating that a set of 35 topsoil fungal species best predicted downy mildew incidence and severity. The lowest error rates for the phyllosphere and leaf endosphere samples were much higher (34% ± 12 and 52% ± 19, respectively) than those for the topsoil samples (Supplementary Fig. S17). Therefore, contrary to hypothesis (iv), the topsoil microbiota was a better predictor of the incidence and severity of leaf symptoms than the leaf microbiota.

In addition, our analyses revealed that the topsoil fungal community can predict symptoms incidence and severity in the following year. The RF algorithm trained on topsoil samples from 2022 successfully predicted the incidence and severity of topsoil samples collected in 2023. The lowest error rate was 6% ± 12, achieved using abundant fungal taxa aggregated at the genus level, and the mean mtry value was 12 (Supplementary Fig. S18). In comparison, phyllosphere and leaf endosphere microbial communities were poorer predictors, with the lowest error rates of 35% ± 15 and 41% ± 17, respectively (Supplementary Fig. S19).

## Discussion

To the best of our knowledge, this study is the first to demonstrate a relationship between disease epidemiology and the host microbiome in a plant pathosystem. To elucidate the interactions between plant diseases and the microbiome, studies typically compare the microbiomes of symptomatic and asymptomatic tissue samples [114–118]. The originality of our study lies in analysing the microbiome of healthy plants only. These plants were selected from plots with contrasting disease incidence and severity, according to long-term epidemiological data. We analyzed the microbiome of young, asymptomatic leaves to test the hypothesis that the microbiome that develops before the pathogen spreads to the leaves can influence or indicate the likelihood of disease development.

Our experimental design was specifically tailored to avoid potential confounding effects that may obscure the relationships between disease epidemiology and the plant microbiome [119]. To achieve this goal, we selected pairs of plots with contrasting disease incidence and severity over the years but otherwise as similar as possible (planted with the same variety, managed with the same practices, and geographically close to each other). We also repeated the sampling over two consecutive years to ensure robust results. Finally, we improved the taxonomic assignment of microbial taxa by sequencing a subset of the samples using long-read technology and by sequencing a collection of cultivable microorganisms isolated from the biological material of the study [60]. Using this approach, we were able to improve the taxonomic assignments for 11% of the fungal ASVs and 10% of the bacterial ASVs. Obtaining the most accurate taxonomic assignments was critical, as the interpretation of our analyses, aimed at identifying candidates for disease biocontrol, largely depended on our ability to assign DNA sequences to specific species.

### Using microbiome data as a predictor of disease incidence and severity

Recent research in human health indicates that microbiome data can enhance the accuracy of disease risk screening and predict dietary and lifestyle changes likely to slow disease progression in individuals [120, 121]. Such predictive approaches, based on microbiome data collected at the individual level, are still in their infancy in terms of plant health. Our study explored the potential of the plant microbiome for precision agriculture [122] by attempting to predict plot incidence and severity of a major disease (grapevine downy mildew caused by *P. viticola*) based on topsoil and leaf microbiomes. Advances in human health and the promising results obtained in this study highlight the potential of using microorganisms to predict health outcomes, both in humans and plants.

In the present study, we showed that the topsoil microbiome in vineyards is an accurate predictor of downy mildew incidence and severity, with an error rate of 15% for within-year predictions and 6% for between-year predictions. Surprisingly, the best predictions of downy mildew incidence and severity, defined by symptoms observed on aerial organs (leaves and grapes), were obtained using topsoil fungal abundance as a predictor. In contrast, foliar communities (from the endosphere and phyllosphere) were poorer predictors, with error rates exceeding 30%. These results may be explained by the fact that the soil is the reservoir of microorganisms that colonize the aerial organs of grapevines [123, 124]. In this study, we sampled the vineyard microbiota early in the growing season. At this time of year, the topsoil contained both microorganisms that can inhibit the germination of downy mildew oospores and microorganisms that can subsequently spread to grapevine leaves, where they can prevent infection by zoospores. With a different sampling strategy, the leaf microbiota might have been a better predictor. For instance, collecting leaf microbiota samples at different times throughout the season could have enhanced their predictive power, as the microbiota exhibits strong dynamics over time [125–127]. Performing predictions at the individual plant level might also increase the predictive power of the leaf microbiota. Indeed, a dysbiotic state, characterized by a temporary loss of the plant's ability to regulate its microbiota at the individual level [128, 129], is considered the proximate cause of disease.

The greater richness of the soil microbiota compared to the leaf microbiota may also explain its higher predictive power. This higher microbial richness enhances the number of variables (i.e., ASVs) that can be used as predictors. Such richness is a biological reality that has been demonstrated in many other studies of plant microbiota [124, 127, 129–131]. Moreover, the difference in microbiota richness between soil and leaves could be exacerbated by molecular biology protocols. In our study, obtaining high-quality DNA in sufficient amounts was more challenging from leaves than from soil. We had less biological material from the phyllosphere, from which DNA was extracted from microbial pellets, compared to soil (100 mg lyophilized soil). Furthermore, grapevine leaves contain high levels of secondary metabolites, such as polysaccharides, polyphenols and tannins, which not only bind to nucleic acids during extraction, leading to their loss, but also act as PCR inhibitors, thus making leaves a challenging environment for microbial characterization [132–134]. For the leaf endosphere, the DNA extracts likely contain more grapevine DNA than microbial DNA, making amplification of target microbial DNA less effective and allowing the amplification of nontarget, contaminating sequences. This problem is particularly true for bacteria because we used degenerate primers to avoid chloroplast amplification of the 16S rRNA gene, which results in less effective amplification [111]. These methodological issues may explain the higher predictive power of the topsoil microbiota over the leaf microbiota, as well as the reason fungi were more effective predictors than bacteria. These results parallel those of Cambon et al. [111], who used the same random forest algorithms (*microranger* R package) to assess the predictive power of the microbiota of several tree species. These authors reported that prediction error rates were inversely correlated with sequencing depth and emphasized that high-quality sequencing data are crucial for accurate microbiota-based predictions [111].

### Relationship between microbiome diversity and disease incidence and severity

Increasing microbial diversity is generally considered beneficial for the health of organisms and ecosystems [135, 136]. Loss of microbial diversity is typically considered a marker of deteriorating health, as it may lead to loss of microbial functions and disruption of microbial interaction networks [135]. However, contrary to these expectations, we did not find a strong relationship between microbial diversity and downy mildew incidence and severity. Only the leaf fungal communities sampled in 2022 had higher diversity in plots with low downy mildew incidence and severity, which is consistent with the Biodiversity-Ecosystem Functioning (BEF) theory [11]. However, these results did not extend to the leaf bacterial communities, nor to the soil fungal and bacterial communities. Furthermore, they could not be replicated in 2023, suggesting that microbiome diversity alone may not be a critical factor in downy mildew incidence and severity.

### Microbial consortia that may interfere with the pathogen asexual stage

Plant pathogens often have complex life cycles that span multiple microbial habitats [137]. In the case of grapevine downy mildew, the asexual stage of the life cycle occurs in green organs during the growing season [12, 13]. During this period, *P. viticola* can interact with microorganisms inhabiting both the phyllosphere and the leaf endosphere. In the present study, we show that interactions with phyllosphere microorganisms, especially basidiomycete yeasts, are more likely than interactions with endophytes. Twenty species of epiphytic basidiomycete yeasts were associated with low downy mildew incidence and severity or low levels of primary pathogen inoculum. They belong to several genera, including *Buckleyzyma*, *Bullera*, *Cystofilobasidium*, *Dioszegia*, *Erythrobasidium*, *Filobasidium*, *Itersonilia*, *Leucosporidium*, *Naganishia*, *Papiliotrema*, *Rhodotorula*, *Tausonia*, and *Vishniacozyma*. The biocontrol activity of basidiomycete yeasts is well documented, as several species are already used as biocontrol agents (BCAs) against postharvest diseases [138–140]. These yeasts regulate plant pathogens through various mechanisms, including competition for nutrients and space, secretion of toxins, enzymes, and volatile organic compounds (VOCs), parasitism, and indirect mechanisms, such as resistance induction [138–140]. These mechanisms could be effective in controlling foliar diseases, including downy mildew.

The yeast species *B. aurantiaca* (previously classified as *Rhodotorula aurantiaca*) is particularly promising, as its higher abundance in low- incidence and severity than in high- incidence and severity plots was confirmed by all 5 methods used. Moreover, this yeast species is known to reduce the incidence of blue mold in pears [141] and the severity of soft rot in peppers [142]. It has also shown promising results in the control of bacterial fruit blotch in melons [143, 144]. In addition, regarding the grape pathogens, *B. aurantiaca* liquid culture reduced the growth of *Erysiphe necator* by approximately 30% and significantly increased the number of collapsed conidia [145]. However, it had no effect on *Botrytis cinerea* [146].

According to our results, other yeast species that may act as a primary line of defense against *P. viticola* in young leaves include *L. scottii*, *F. oeirense*, *V. carnescen*s, and *B. alba*. The yeast *L. scottii* has been identified as a good BCA against apple blue and gray mold caused by *Penicillium expansum* and *B. cinerea*, respectively [147]. *F. oeirense* and *V. carnescens* are known to inhibit *B. cinerea* development through VOCs production [148]. *B. alba* has been shown to produce a lethal toxin that inhibits many yeast-like ascomycete and basidiomycete fungi and to have an excellent biocontrol effect on apple gray mold [149, 150].

Finally, we found that the ubiquitous ascomycete yeast *A. pullulans* is more abundant in vineyard plots with low downy mildew incidence and severity. The biocontrol activity of this yeast species is well known. In France, it is commercialized in products such as BOTECTOR®, CINERKIL, AUREO SHIELD, and Blossom Protect™. The presence of yeast consortia early in the season in the phyllosphere of low- incidence and severity plots seems to be a good indicator of plot downy mildew incidence and severity.

### Microbial consortia that may interfere with the pathogen sexual stage

In contrast to the asexual stage, the sexual stage of the downy mildew life cycle occurs primarily in the soil during fall and winter [12, 13]. *Plasmopara viticola* overwinters as oospores in the topsoil, where it can interact with the multitude of microorganisms present in this biodiversity reservoir. These belowground interactions have been largely overlooked, but our results suggest they exist, as we found several hundred fungal and bacterial ASVs that varied in abundance between plots with high and low downy mildew incidence and severity. Among these numerous ASVs, some may directly affect oospore survival and germination, while others are likely mere indicators of soil conditions unfavorable to oospores [151].

In addition, we showed that the amount of *P. viticola* DNA in the topsoil is significantly lower in plots classified as having low downy mildew incidence and severity, based on epidemiological records of symptoms observed on leaves and bunches. This correlation between the amount of oospores in the topsoil and disease incidence and severity in aerial organs suggests that reducing primary pathogen inoculum in the soil through prophylaxis or soil microbial management [22, 152] could be an effective strategy for controlling disease epidemics. However, effective soil microbial management requires clear targets. Our analyses provide some insight into the soil microbial taxa that may serve as relevant targets.

Our analyses revealed several fungal species that were significantly more abundant in the topsoil of vineyard plots with low downy mildew incidence and severity compared to those with high incidence and severity. Among them, four species are known for their biocontrol activity against the asexual stage of downy mildew: *Albifimbria verrucaria* [153], *A. alternata* [154, 155], *F. brachygibbosum* [40, 156], and *E. nigrum* [34]. Our analyses do not allow us to determine whether these fungal species detected in topsoil samples interact with downy mildew oospores (sexual stage) in the topsoil or if they first colonize young leaves and then interact with downy mildew zoospores (asexual stage).

In addition, our results revealed that several basidiomycetous yeasts were more abundant in the topsoil of plots with low downy mildew incidence and severity. Among them, four species were also more abundant in the phyllosphere of vineyard plots with low downy mildew incidence and severity. All four are known for their biocontrol properties against plant pathogens: *B. alba* [149, 150], *F. oeirense* [157], *P. laurentii* [158, 159], and *P. terrestris* (previously classified as *Cryptococcus laurentii*) [160, 161]. These congruent results between topsoil and young leaves indicate that both microbial habitats are connected by microorganism dispersal at the beginning of the growing season, suggesting that management of the topsoil microbiota may influence the phyllosphere microbiota composition.

Filamentous fungi with known biocontrol properties were also found in higher abundance in the topsoil of vineyard plots with low downy mildew incidence and severity. These included *Bjerkandera adusta* [162], *Issatchenkia orientalis* [163–165], *M. alpina* [166], and two species of *Trichoderma* (*T. lixii* and *T. virens*). While the biocontrol properties of *T. lixii* have not been studied, *T. virens* is a well-established biocontrol agent with the ability to produce antibiotics, parasitize pathogenic fungi, and induce systemic resistance in plants [167, 168]. Strains of *Trichoderma* are widely distributed in soil environments and can parasitize a wide range of plant pathogens, including the downy and powdery mildews of grapevines [169–172]. The higher abundance of these microorganisms in low- incidence and severity plots compared to high- incidence and severity plots supports our comparative approach.

Soil bacteria of the genus *Streptomyces* (including *S. albidoflavus*, *S. ambofaciens*, *S. hygroscopicus*, and *S. xinghaiensis*) were also associated with low downy mildew incidence and severity. They stand out as promising candidates for the biocontrol of downy mildew because of their well-documented properties. Streptomyces species have previously been identified as effective biocontrol agents against oomycete pathogens, including grapevine downy mildew, owing to their potent antimicrobial properties [27, 39, 173, 174]. For example, Abdalla et al. [30] demonstrated that khatmiamycin, a compound isolated from a terrestrial *Streptomyces* species, exhibited inhibitory and lytic activities against the zoospores of *P. viticola*. Similarly, Islam et al. [175] reported that extracts from several marine *Streptomyces* strains impaired zoospore motility and caused their lysis. Antimicrobial substances in the fermentation broth of the strain *Streptomyces atratus* PY-1 were also shown to be effective against downy mildew by damaging sporangia and sporangiophores [39]. Finally, El-Sharkawy et al. [176] reported that *Streptomyces viridosporus* HH1 and *Streptomyces violaceus* HH5 can reduce the severity of downy mildew in the field. These results suggest that soil-borne microorganisms could prevent the infection of aerial parts by *P. viticola*. During the winter season, the soil could act as a reservoir for protective strains that would colonize the aerial parts of the vines later in the growing season.

### Environmental and anthropogenic drivers of microbiota composition

According to our results, the microbial taxa that varied in abundance between the low- and high- incidence and severity plots were both rare and abundant. Their relative abundance ranged from approximately 0.00006% to 14% of the total number of sequences. Increasing the abundance of rare taxa of interest through inoculation with microbial strains or consortia seems feasible, but increasing the abundance of already abundant taxa that may be more challenging. In the latter case, microbiota management could rely on changes in vineyard management practices. Our analysis provides some insight into the practices that may have an impact on the resident microbiota.

Our analyses revealed that grape variety had small but significant effects on the microbiota composition. The significant influence of grape variety on both leaf and soil microbiota is consistent with findings from other studies [129, 177–182]. This is an interesting result, as genotype can influence resistance to downy mildew [183] and may also affect the recruitment of microorganisms with biocontrol properties that could protect the plant from pathogens.

In addition, our results suggest that reducing the frequency of fungicide treatments could alter the composition of the microbiota and increase microbial diversity. The absence of fungicide application increased microbial diversity in the soil, as documented in other studies [184–186]. It also increased microbial diversity in the phyllosphere the following spring, probably due to the dispersal of microorganisms from the soil to the leaves. Finally, proximity to the plot edge increased microbial diversity in the topsoil, phyllosphere, and endosphere. These results parallel those of Ricono et al. [187], who reported a decrease in diversity with increasing distance from the edge in the endosphere of wheat roots. In vineyard agroecosystems, several studies have demonstrated the importance semi-natural habitats in enhancing biodiversity and farm productivity through its influence on pest control services provided by biological control [188]. These findings suggest that edge management may be an option to increase microbial diversity in vineyard plots.

## Conclusion and perspectives

We identified bacterial and fungal taxa naturally occurring in vineyard environments that may be promising candidates for the biocontrol of downy mildew. These microorganisms may play a role in disrupting either the sexual or asexual stages of the pathogen, offering potential alternatives to conventional control methods. Specifically, fungi from the genera *Rhodotorula*, *Buckleyzyma*, *Vishniacozyma*, and *Filobasidium* may target the asexual stage, while fungi from *Trichoderma*, *Mortierella*, and *Papiliotrema* could affect the sexual stage. Additionally, bacteria from the genera *Arthrobacter*, *Bacillus*, *Streptomyces*, and *Pseudarthrobacter* may play a role in controlling the sexual stage. The results of this study open the door to further research aimed at assessing the efficacy and sustainability of these biocontrol agents under different climatic conditions, soil types and vineyard management practices. After isolation, a deeper understanding of their mode of action and interactions with the pathogen will be essential for the development of more robust and environmentally friendly integrated management strategies to protect vineyards from downy mildew. Our study also provides insights into vineyard management practices that could help harness vineyard resident microbiota. Our results suggest, for instance, that edge management may impact microbial diversity. Additionally, we found that specific soil microbial taxa can predict disease incidence and severity with an average accuracy of 85%. This surprising finding, given that the disease is airborne, highlights the potential for integrating soil microbiota data into disease surveillance strategies [189].

## Supplementary Information

Supplementary Material 1. Methods S1: Amplification trial with the primer pair ITS1catta—ITS2ngs. Methods S2: Custom reference database built from a microbial culture collection. Methods S3: Taxonomic assignments using both public and custom reference databases. Figure S1: Map of vineyard plots monitored for downy mildew incidence and severity. Figure S2: Sampling design. Figure S3: Comparison of microbial community profiles for the leaf endosphere depending on the primer pair. Figure S4: Oomycetes detected in the leaf endosphere using the ITS1catta-ITS2ngs primer pair. Figure S5: Decision tree for assigning fungal (nrDNA ITS gene) and bacterial (16S rRNA gene) sequences. Figure S6: Disease progression curves for the 7 plot pairs. Figure S7: Variation in downy mildew primary inoculum in topsoil. Figure S8: Microbial community profiles depending on downy mildew incidence and severity in vineyard plots. Figure S9: Variation in microbial community composition across vineyard plots. Figure S10: Environmental factors driving variation in microbial community composition in 2022. Figure S11: Environmental factors driving variation in microbial community composition in 2023. Figure S12: Variations in the diversity of microbial communities depending on downy mildew incidence and severity in vineyard plots (2022 data). Figure S13: Variations in the diversity of microbial communities depending on downy mildew incidence and severity in vineyard plots (2023 data). Figure S14: Endosphere fungal taxa that vary in abundance with downy mildew incidence and severity. Figure S15: Topsoil fungal taxa that vary in abundance with downy mildew incidence and severity. Figure S16: Phyllosphere bacterial taxa that vary in abundance with downy mildew incidence and severity. Figure S17: Random Forest algorithm performance in predicting grapevine downy mildew incidence and severity using leaf microbiota composition. Figure S18: Random Forest algorithm performance in predicting grapevine downy mildew incidence and severity using topsoil microbiota data year-to-year. Figure S19: Random Forest algorithm performance in predicting grapevine downy mildew incidence and severity using leaf microbiota data year-to-year. Table S1: Environmental variables used to explain the variation in microbiota composition. Table S2: Factors driving variation in downy mildew inoculum in topsoil. Table S3: Summary of read and ASV loss during data processing. Table S4: Most abundant fungal species in the topsoil, phyllosphere and leaf endosphere of vineyard plots (2022 data). Table S5: Most abundant bacterial genera in the topsoil, phyllosphere and leaf endosphere of vineyard plots (2022 data). Table S6: Most abundant fungal species in the topsoil, phyllosphere and leaf endosphere of vineyard plots (2023 data). Table S7: Most abundant bacterial genera in the topsoil, phyllosphere and leaf endosphere of vineyard plots (2023 data). Table S8: Factors driving variation in microbiota alpha diversity (2022 data). Table S9: Factors driving variation in microbiota alpha diversity (2023 data). Table S10: Fungal taxa indicators of low downy mildew primary inoculum in topsoil. Table S11: Bacterial taxa indicators of low downy mildew primary inoculum in topsoil.

Supplementary Material 2. Factors influencing the beta diversity of the microbiota.

Supplementary Material 3. Microbial ASVs significantly more abundant in plots with high versus low downy mildew incidence and severity.

Supplementary Material 4. Fungal and bacterial ASVs indicating low and high *Plasmopara viticola* DNA concentrations in topsoil.

## Data Availability

The raw sequence data obtained from Illumina and PacBio sequencing were deposited in the European Nucleotide Archive (ENA) under the project numbers PRJEB79310 and PRJEB79661, respectively. The raw sequence data obtained from Sanger sequencing were deposited in the National Center for Biotechnology Information (NCBI) under the Accession Numbers PQ348104—PQ348540 for bacteria and PQ350423—PQ350897 for fungi and yeasts. The filtered and assembled sequence data, the metadata, and the scripts for bioinformatic and statistical analyses are available on Recherche Data Gouv under the DOI [https://doi.org/10.57745/XNEH5O].

## Footnotes

1 https://metabarfactory.github.io/metabaR/articles/metabaRF-vignette.html.
