# Supplementary material for: Revealing microbial consortia that interfere with grapevine downy mildew through microbiome epidemiology
Source: Environ Microbiome. 2025 Mar 27;20:37. doi: 10.1186/s40793-025-00691-9 (PMC11948771; doi:10.1186/s40793-025-00691-9)
Supplement: Supplementary file 1 — Supplementary Material 1. Methods S1: Amplification trial with the primer pair ITS1catta—ITS2ngs. Methods S2: Custom reference database built from a microbial culture collection. Methods S3: Taxonomic assignments using both public and custom reference databases. Figure S1: Map of vineyard plots monitored for downy mildew incidence and severity. Figure S2: Sampling design. Figure S3: Comparison of microbial community profiles for the leaf endosphere depending on the primer pair. Figure S4: Oomycetes detected in the leaf endosphere using the ITS1catta-ITS2ngs primer pair. Figure S5: Decision tree for assigning fungal (nrDNA ITS gene) and bacterial (16S rRNA gene) sequences. Figure S6: Disease progression curves for the 7 plot pairs. Figure S7: Variation in downy mildew primary inoculum in topsoil. Figure S8: Microbial community profiles depending on downy mildew incidence and severity in vineyard plots. Figure S9: Variation in microbial community composition across vineyard plots. Figure S10: Environmental factors driving variation in microbial community composition in 2022. Figure S11: Environmental factors driving variation in microbial community composition in 2023. Figure S12: Variations in the diversity of microbial communities depending on downy mildew incidence and severity in vineyard plots (2022 data). Figure S13: Variations in the diversity of microbial communities depending on downy mildew incidence and severity in vineyard plots (2023 data). Figure S14: Endosphere fungal taxa that vary in abundance with downy mildew incidence and severity. Figure S15: Topsoil fungal taxa that vary in abundance with downy mildew incidence and severity. Figure S16: Phyllosphere bacterial taxa that vary in abundance with downy mildew incidence and severity. Figure S17: Random Forest algorithm performance in predicting grapevine downy mildew incidence and severity using leaf microbiota composition. Figure S18: Random Forest algorithm performance in predicting grapevine downy m [file 40793_2025_691_MOESM1_ESM.docx]

**Revealing microbial consortia that interfere with grapevine downy mildew through microbiome epidemiology**

*Paola FOURNIER (1), Lucile PELLAN (1), Aarti JASWA (1), Marine C. CAMBON (2), Alexandre CHATAIGNER (1), Olivier BONNARD (3), Marc RAYNAL (4), Christian DEBORD (4), Charlotte POEYDEBAT (5), Simon LABARTHE (3), François DELMOTTE (1), Patrice THIS (6), Corinne VACHER (1*)*

1. *INRAE, Bordeaux Sciences Agro, ISVV, SAVE, Villenave-d’Ornon, France*
2. *School of Biosciences, Birmingham Institute of Forest Research, Institute of Microbiology and Infection, University of Birmingham, United Kingdom*
3. *Univ. Bordeaux, INRAE, BIOGECO, Cestas, France*
4. *IFV, Blanquefort, France*
5. *Bordeaux Sciences Agro, INRAE, ISVV, SAVE, Villenave-d’Ornon, France*
6. *UMR AGAP Institut, Univ. Montpellier, CIRAD, INRAE, Institut Agro, Montpellier, France*

**Supplementary File S1**

**Table of contents**

[Supplementary methods 1](#_Toc191289706)

[Methods S1: Amplification trial with the primer pair ITS1catta - ITS2ngs. 1](#_Toc191289707)

[Methods S2: Custom reference database built from a microbial culture collection. 2](#_Toc191289708)

[Methods S3: Taxonomic assignments using both public and custom reference databases. 3](#_Toc191289709)

[Supplementary figures 4](#_Toc191289710)

[Figure S1: Map of vineyard plots monitored for downy mildew incidence and severity. 4](#_Toc191289711)

[Figure S2: Sampling design. 5](#_Toc191289712)

[Figure S3: Comparison of microbial community profiles for the leaf endosphere depending on the primer pair. 6](#_Toc191289713)

[Figure S4: Oomycetes detected in the leaf endosphere using the ITS1catta-ITS2ngs primer pair. 7](#_Toc191289714)

[Figure S5: Decision tree for assigning fungal (nrDNA ITS gene) and bacterial (16S rRNA gene) sequences. 8](#_Toc191289715)

[Figure S6: Disease progression curves for the 7 plot pairs. 10](#_Toc191289716)

[Figure S7: Variation in downy mildew primary inoculum in topsoil. 20](#_Toc191289717)

[Figure S8: Microbial community profiles depending on downy mildew incidence and severity in vineyard plots. 21](#_Toc191289718)

[Figure S9: Variation in microbial community composition across vineyard plots. 22](#_Toc191289719)

[Figure S10: Environmental factors driving variation in microbial community composition in 2022. 23](#_Toc191289720)

[Figure S11: Environmental factors driving variation in microbial community composition in 2023. 24](#_Toc191289721)

[Figure S12: Variations in the diversity of microbial communities depending on downy mildew incidence and severity in vineyard plots (2022 data). 25](#_Toc191289722)

[Figure S13: Variations in the diversity of microbial communities depending on downy mildew incidence and severity in vineyard plots (2023 data). 26](#_Toc191289723)

[Figure S14: Endosphere fungal taxa that vary in abundance with downy mildew incidence and severity. 27](#_Toc191289724)

[Figure S15: Topsoil fungal taxa that vary in abundance with downy mildew incidence and severity. 28](#_Toc191289725)

[Figure S16: Phyllosphere bacterial taxa that vary in abundance with downy mildew incidence and severity. 29](#_Toc191289726)

[Figure S17: Random Forest algorithm performance in predicting grapevine downy mildew incidence and severity using leaf microbiota composition. 31](#_Toc191289727)

[Figure S18: Random Forest algorithm performance in predicting grapevine downy mildew incidence and severity using topsoil microbiota data year-to-year. 32](#_Toc191289728)

[Figure S19: Random Forest algorithm performance in predicting grapevine downy mildew incidence and severity using leaf microbiota data year-to-year. 33](#_Toc191289729)

[Supplementary tables 34](#_Toc191289730)

[Table S1: Environmental variables used to explain the variation in microbiota composition. 34](#_Toc191289731)

[Table S2: Factors driving variation in downy mildew inoculum in topsoil. 35](#_Toc191289732)

[Table S3: Summary of read and ASV loss during data processing. 36](#_Toc191289733)

[Table S4: Most abundant fungal species in the topsoil, phyllosphere and leaf endosphere of vineyard plots (2022 data). 37](#_Toc191289734)

[Table S5: Most abundant bacterial genera in the topsoil, phyllosphere and leaf endosphere of vineyard plots (2022 data). 38](#_Toc191289735)

[Table S6: Most abundant fungal species in the topsoil, phyllosphere and leaf endosphere of vineyard plots (2023 data). 39](#_Toc191289736)

[Table S7: Most abundant bacterial genera in the topsoil, phyllosphere and leaf endosphere of vineyard plots (2023 data). 40](#_Toc191289737)

[Table S8: Factors driving variation in microbiota alpha diversity (2022 data). 41](#_Toc191289738)

[Table S9: Factors driving variation in microbiota alpha diversity (2023 data). 43](#_Toc191289739)

[Table S10: Fungal taxa indicators of low downy mildew primary inoculum in topsoil. 44](#_Toc191289740)

[Table S11: Bacterial taxa indicators of low downy mildew primary inoculum in topsoil. 45](#_Toc191289741)

[References 46](#_Toc191289742)

## Supplementary methods

### Methods S1: Amplification trial with the primer pair ITS1catta - ITS2ngs.

To characterize fungal communities interacting with the oomycete *Plasmopara viticola*, we tested the primer pair ITS1catta - ITS2ngs [1, 2] because it targets the internal transcribed spacer 1 (ITS1) region of the nuclear ribosomal DNA (nrDNA) ITS gene of both fungi and oomycetes and was used successfully on grapevine [3]. We confirmed that these primers indeed amplified the nrDNA ITS gene of both fungi and oomycete by performing PCR on the DNA of two fungal strains (*Debaromyces hansenii* and *Wallemia sebi*) and a strain of *P. viticola*. However, when applied to endosphere samples, the primer pair lacked specificity. We observed variable PCR profiles between endosphere samples on agarose gels, with the number of bands ranging from 1 to 4 and the intensity of the bands varying among samples. Sequencing of a few PCR products obtained using the ITS1catta - ITS2ngs primer pair confirmed the amplification of nontarget sequences (Fig. S3). Although the ITS1catta - ITS2ngs primer pair successfully amplified both fungi and oomycetes (represented by 5 ASVs; Fig. S4), a significant portion of the sequences was also attributed to other groups, including Alveolata, Metazoa, Rhizaria, and Viridiplantae (Fig. S3). In contrast, the ITS1F-ITS2 primer pair was highly specific to the fungal nrDNA ITS gene, with 100% of the sequences belonging to fungi (Fig. S3). These findings parallel those obtained by Savian et al. [4]. Using the ITS1catta - ITS4ngs primer pair, they reported that some samples presented a large band (750 bp) on agarose gels, probably due to amplification of plant-derived DNA. Therefore, we chose the ITS1F‒ITS2 primer pair for our study. This primer pair allowed us to explore a greater range of fungal diversity than the ITS1catta-ITS2ngs primer. In our trial, the ITS1F-ITS2 dataset consisted of 110 fungal ASVs, while the ITS1catta-ITS2ngs dataset included only 74 ASVs.

### Methods S2: Custom reference database built from a microbial culture collection.

Building a custom reference database is recommended for assigning metabarcoding sequence data since including species from the regional species pool increases the reliability of taxonomic assignments [5]. Therefore, we isolated cultivable fungal and bacterial strains from grapevine leaves collected from two pairs of plots (ME1 and ME2; Table 1) using four culture media. Leaves were collected from the center (CEN) of each plot on the same day as the leaves collected for the present study. We obtained 1008 isolates (462 bacteria, 547 fungi), which were identified by Sanger sequencing of the 16S region for bacteria using the 8f [6] and 1115r [7] primer pair, and the nrDNA ITS gene for fungi using the ITS1F‒ITS4 primer pair [8, 9]. Taxonomic assignments of the Sanger sequences were made using the BLASTn+ algorithm [10], with the NCBI standard database used as a reference. In the case of fungi, our final custom reference database consisted of the Sanger sequences of cultivable microorganisms. For bacteria, we extended the Sanger database by adding the 16S longreads obtained using PacBio technology and assigned using BLASTn+ and RDPClassifier against SILVA 138.1 [11].

### Methods S3: Taxonomic assignments using both public and custom reference databases.

We used three methods to assign the metabarcoding sequence data. For bacteria, we used (1) the BLASTn+ algorithm [10] with SILVA 138.1 [11] as the reference database; (2) the BLASTn+ algorithm with our custom reference database; and (3) the RDPClassifier algorithm [12] with SILVA 138.1 as the reference database. The same approach was used for fungi, except that UNITE Fungi 8.3 [13] was used as the public reference database.

To choose between the different taxonomic assignments available for a given ASV, we built decision trees (Fig. S5). We considered that assignments obtained using BLASTn+ were more reliable than those obtained using RDPClassifier [14]. If an ASV was successfully assigned to the species level using BLASTn+ (*i.e.,* e-value <= e-20, percentage identity >=97% for fungi and >=99% for bacteria, query coverage >=90%) [13, 15–17], with both the public database and our custom database, we compared the e-values obtained with both databases. The ASV was assigned according to which database provided the best score (lowest e value). If an ASV was successfully assigned to the species level using BLASTn+ with only one database, it was assigned using this database. If an ASV could not be assigned using BLASTn+, the RDPClassifier assignment was considered. The ASV was assigned to the lowest taxonomic level with a confidence score >= 0.8 [18, 19]. ASVs with a confidence score < 0.8 at the phylum level were discarded [20]. Finally, we discarded the ASVs assigned to the archaea, cyanobacteria, chloroplast and mitochondria.

## Supplementary figures

### Figure S1: Map of the vineyard plots monitored for downy mildew incidence and severity.

Each blue dot represents a vineyard plot including a set of untreated control vines (UCs). The incidence and severity of downy mildew are monitored in each UC by the *Institut Français de la Vigne et du Vin* (IFV). These epidemiological records were used to select pairs of vineyard plots for the present study.


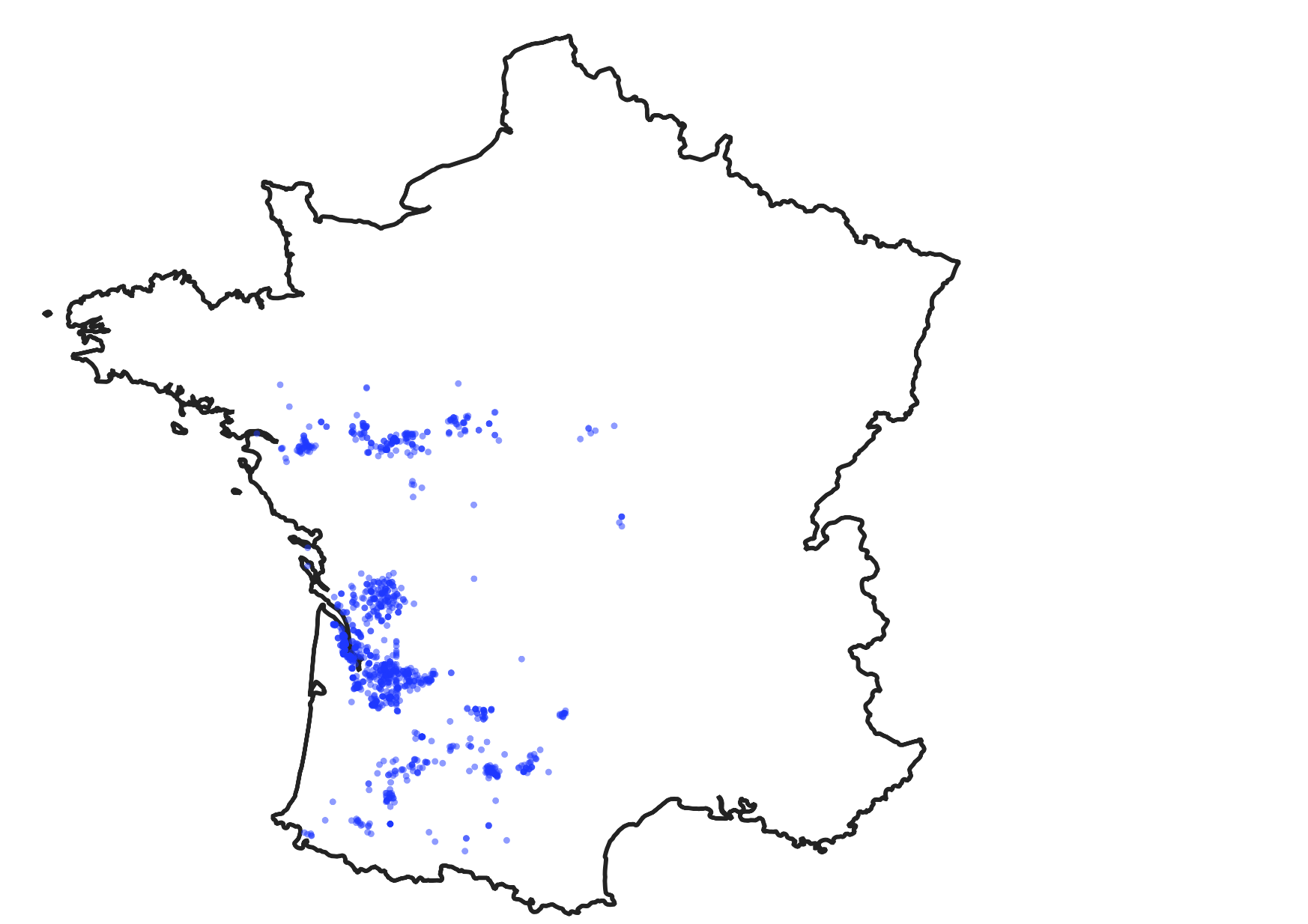


### **Figure S2: Sampling design.**

In each sampling area, we selected four adjacent vines in the same row, typically between two stakes and representative of the age and condition of the plot. Six leaves distributed across the entire plant were collected and pooled into a single composite sample. For the soil, three 20 × 20 cm quadrats were defined at a distance of 20 cm from the trunk of each vine. Two quadrats were directly under the row of vines, and one was in the interrow space. The top 5 cm of soil from each quadrat was collected, mixed and placed into a bag to form a composite sample.


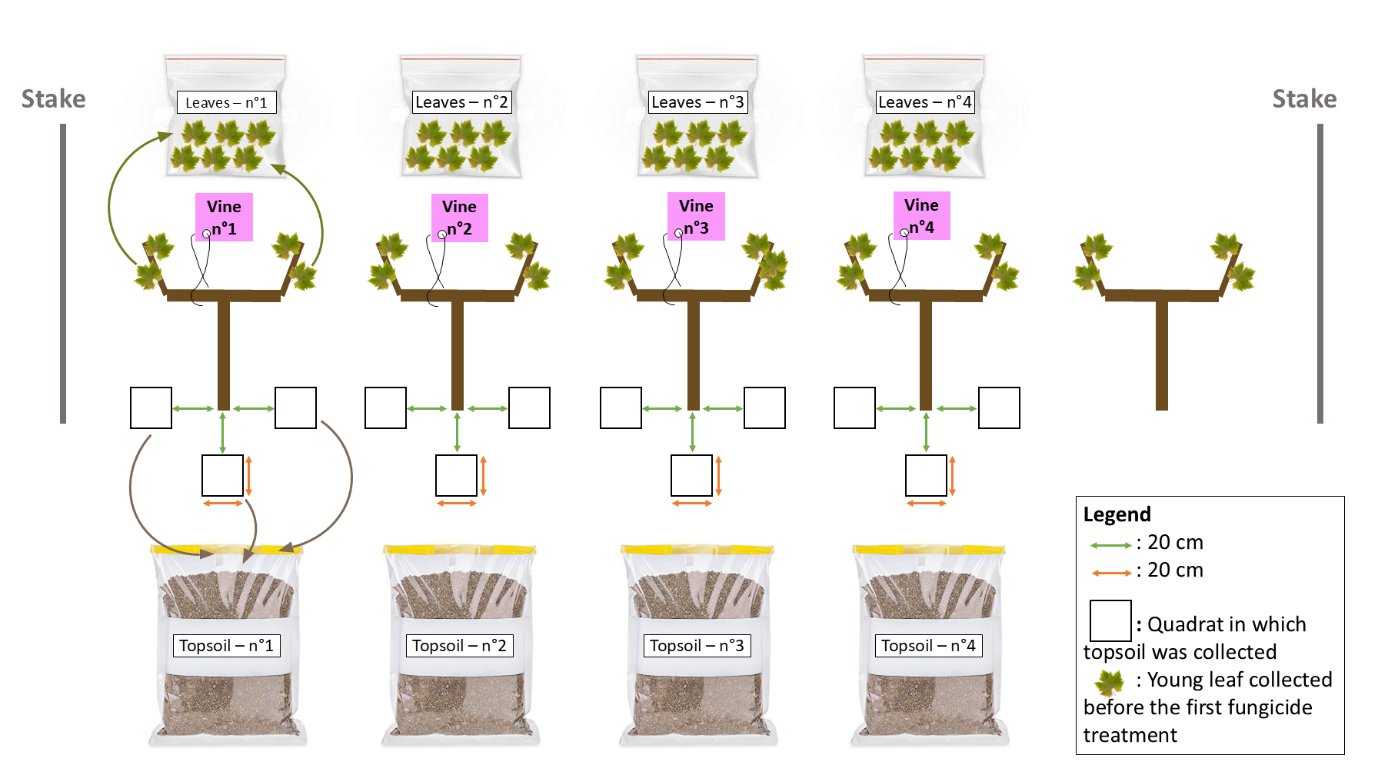


### **Figure S3: Comparison of microbial community profiles for the leaf endosphere depending on the primer pair.**

Six endosphere samples amplified with both the ITS1catta-ITS2ngs primer pair and the ITS1F-ITS2 primer pair were sequenced. These six samples were selected because they had different amplification profiles on agarose gels with the ITS1catta-ITS2ngs primer pair (number of bands ranging from 1 to 4, contrasting intensity of bands among samples). The bar plot represents the proportion of sequences assigned to each domain (A) and phylum (B) in each sample. For fungi, phyla representing less than 1% of sequences were grouped into the 'other' category. To ensure a fair comparison between the two primer pairs, some modifications were made to the bioinformatic pipeline described in the main text. Specifically, the ITSx step, which extracts the highly variable ITS1 and ITS2 subregions, was omitted, and taxonomic assignments were performed using UNITE Eukaryote 8.3 as the public reference database.


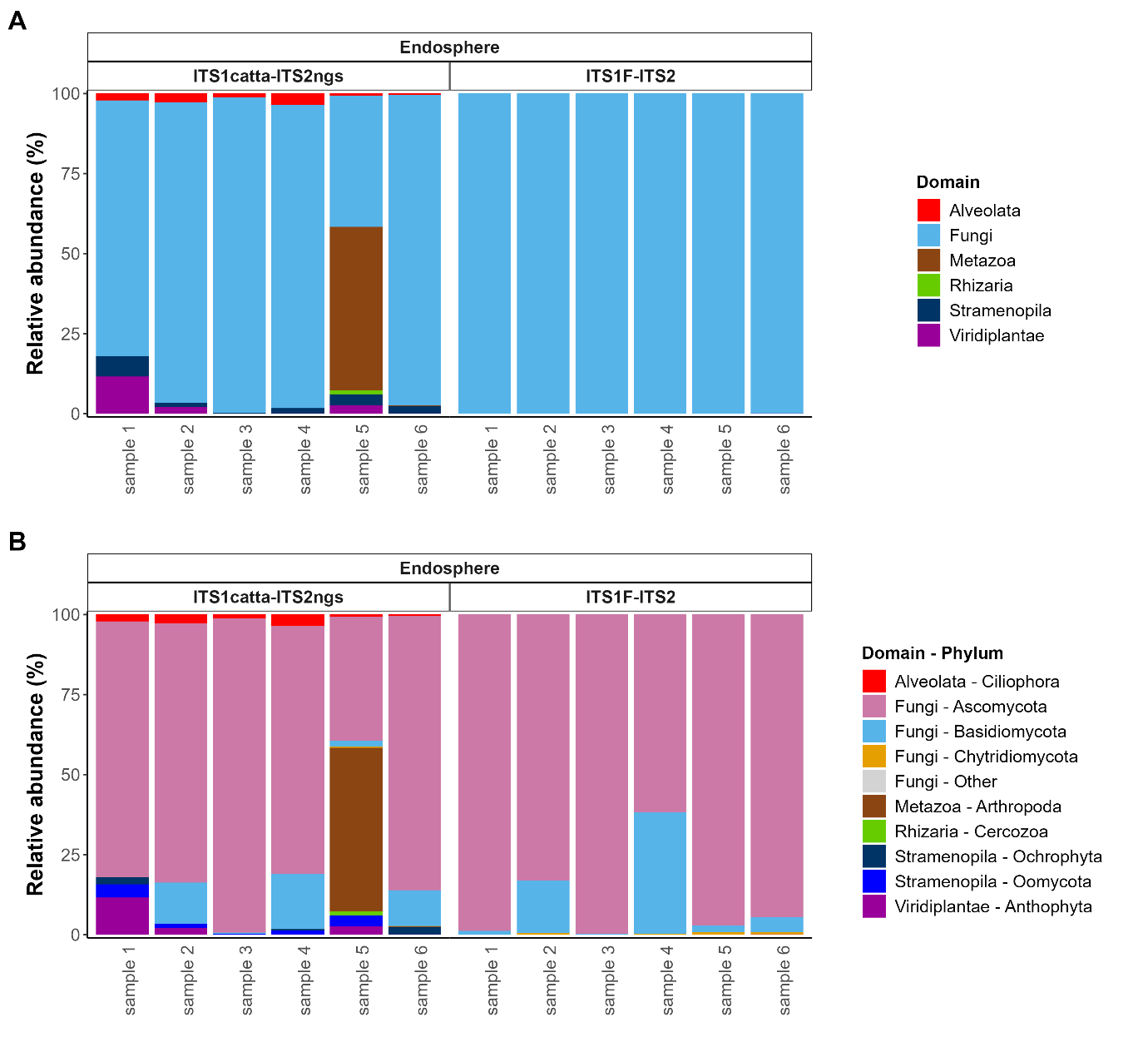


### Figure S4: Oomycetes detected in the leaf endosphere using the ITS1catta-ITS2ngs primer pair.

The figure shows the relative abundance of oomycetes among the six endosphere samples that were sequenced with the ITS1catta-ITS2ngs primer pair. Five ASVs assigned to the OOmycota phylum were detected. The bar plot represents their relative abundance. Note that the ASV (ASV28) assigned to Saprolegnia maragheica was also found in sample 3, with a relative abundance of 0.0007%, which is not visible in the figure.


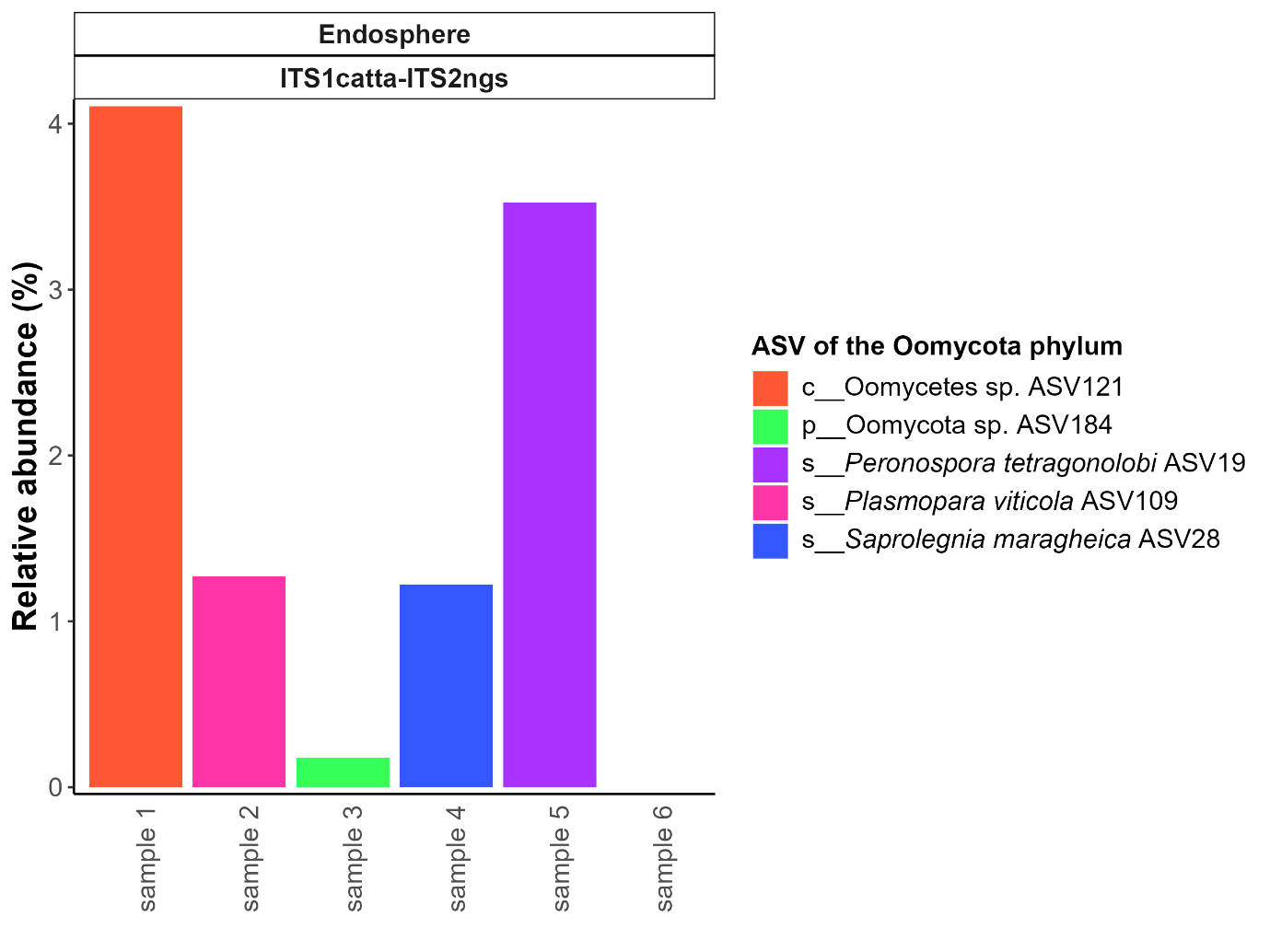


### **Figure S5: Decision tree for assigning fungal (nrDNA ITS gene) and bacterial (16S rRNA gene) sequences.**

Each ASV was assigned using a combination of two tools (BLASTn+ and the RDPClassifier algorithm) and two databases (a public reference database and a custom database) (Supplementary Methods S2 and S3). The decision tree describes the questions that allowed us to choose the most reliable taxonomic assignment. The tables indicate the number of ASVs corresponding to each response (yes or no). The final dataset is the whole dataset, corresponding to the microbial habitats (topsoil, phyllosphere and leaf endosphere) and the two sampling campaigns (2022 and 2023).


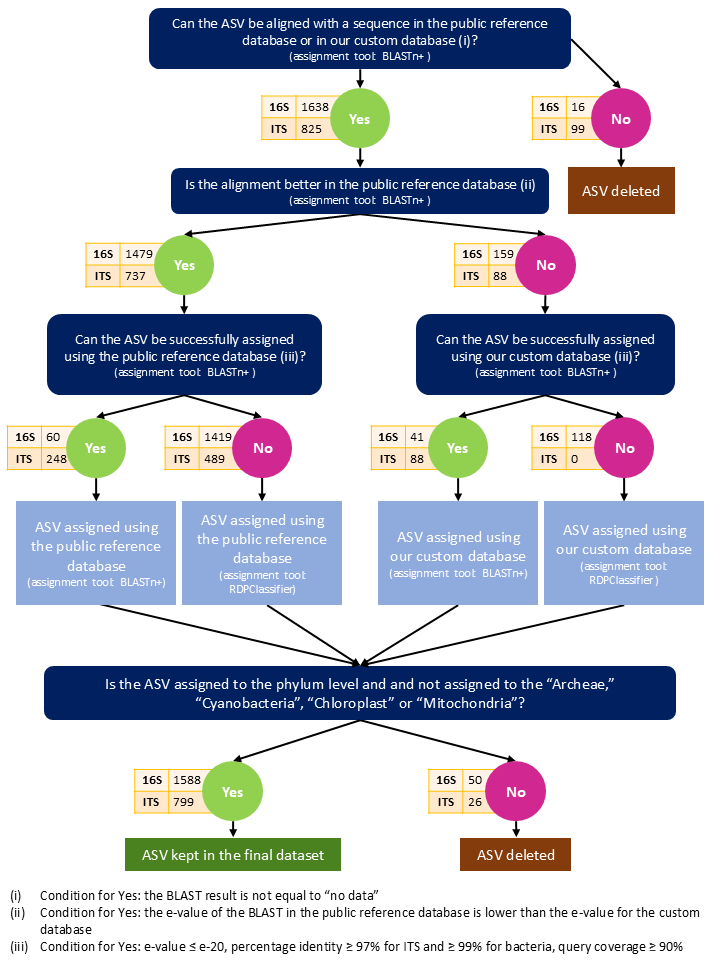


### **Figure S6: Disease progression curves** **for the 7 plot pairs.**

Disease curves for the 4 epidemiological variables measured by visual estimation by vine growers or IFV technicians (severity of symptoms on leaves, incidence of symptoms on leaves, severity of symptoms on bunches, incidence of symptoms on bunches) are shown for the pairs CDB1, CDB2, E2M, LIB, ME1 and ME3 across all years of joint monitoring of the two plots forming each pair. **For the pair LIB, the curves are split into two figures** due to 11 years of joint monitoring. For better readability, the first figure shows 2013–2017, and the second covers 2018–2023. Estimations are expressed as percentages.


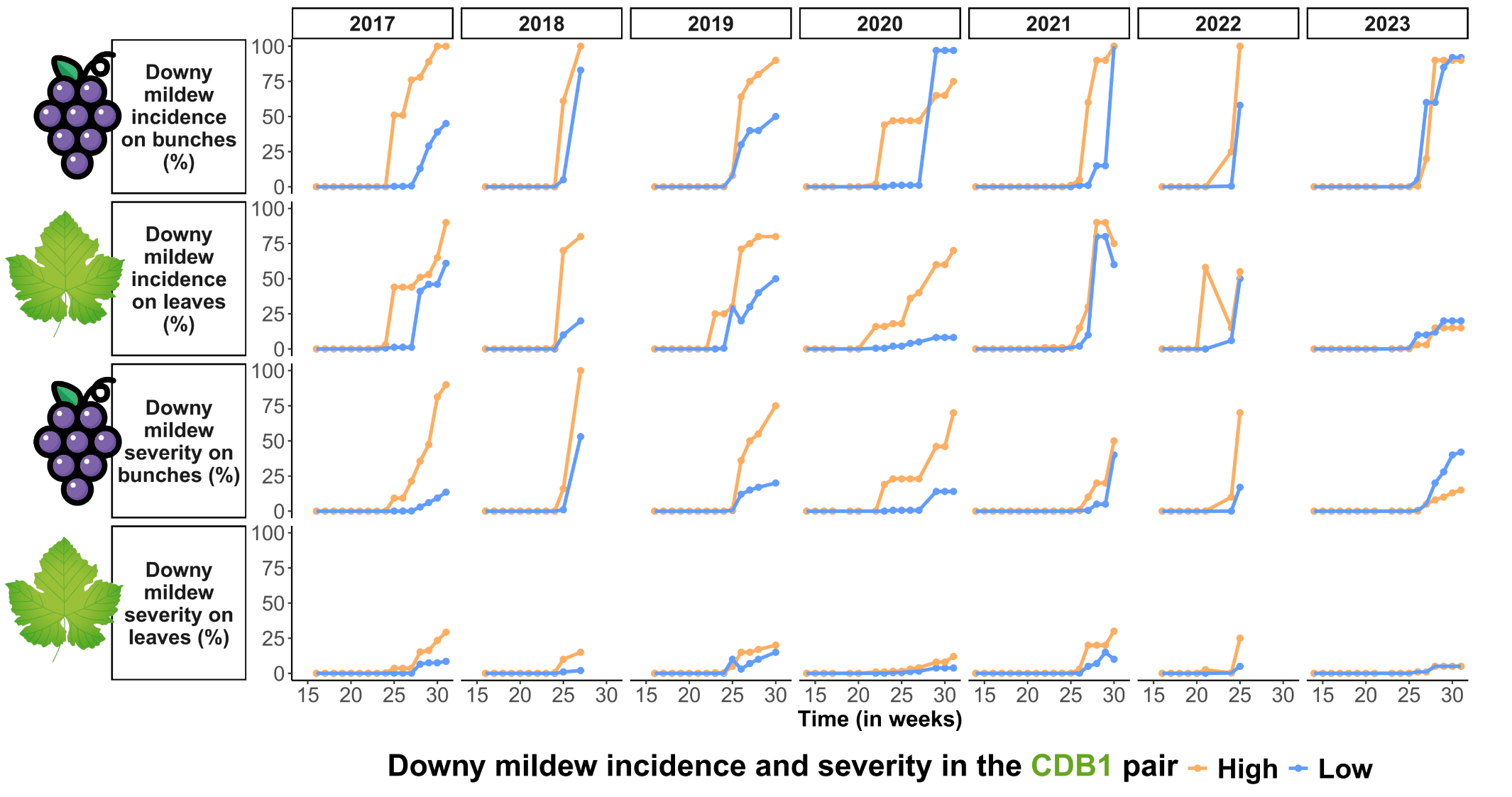

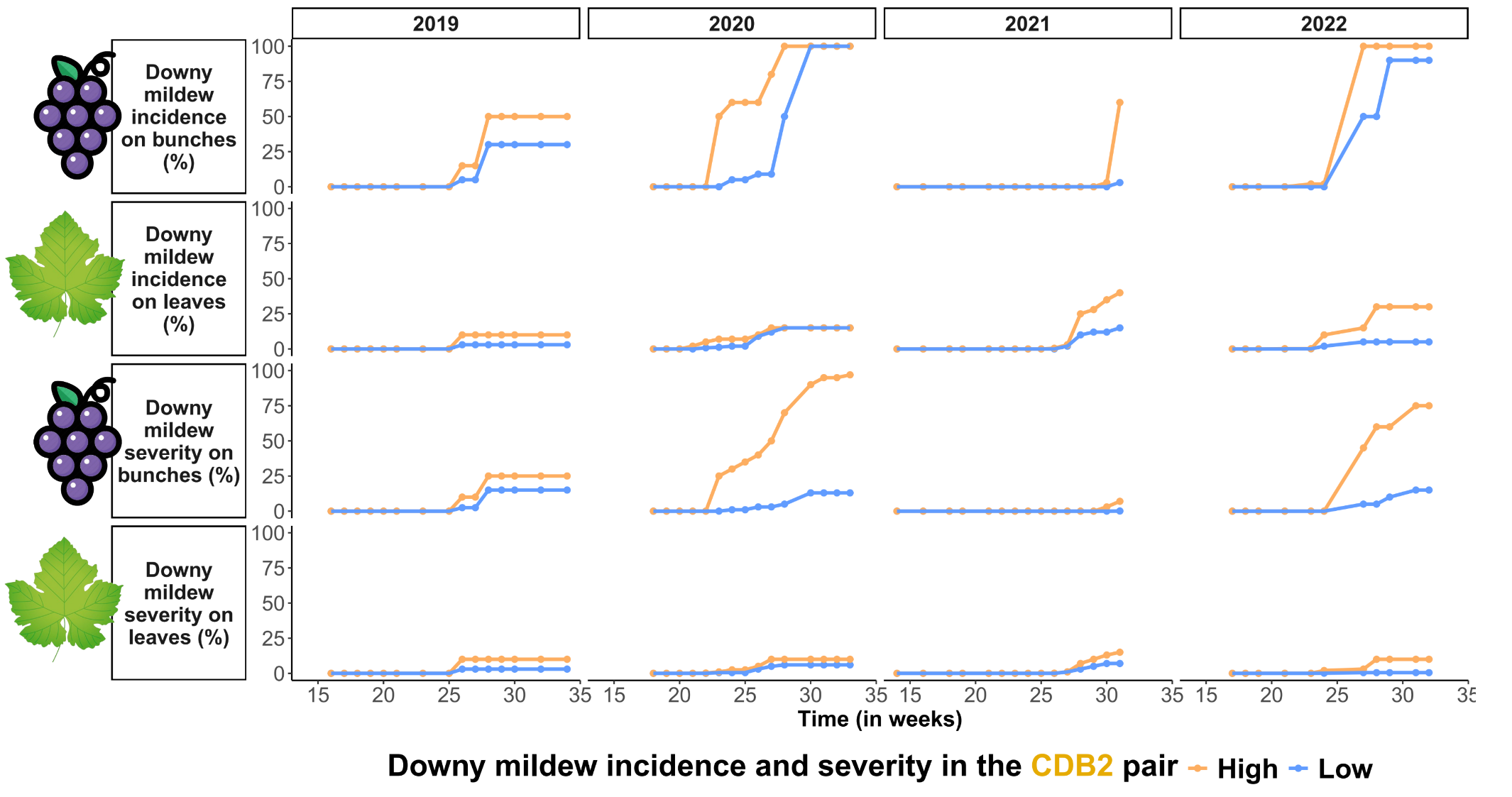

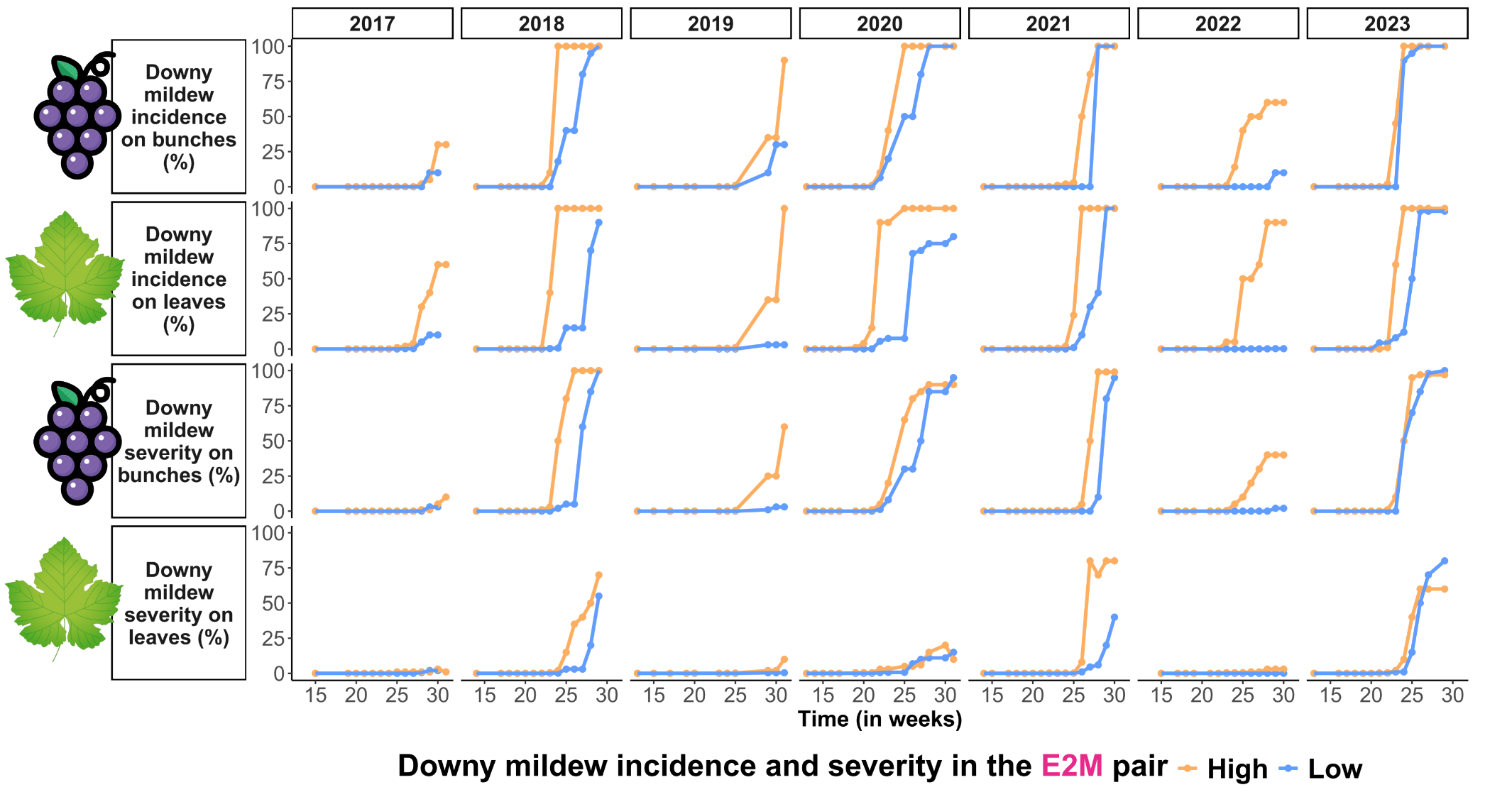

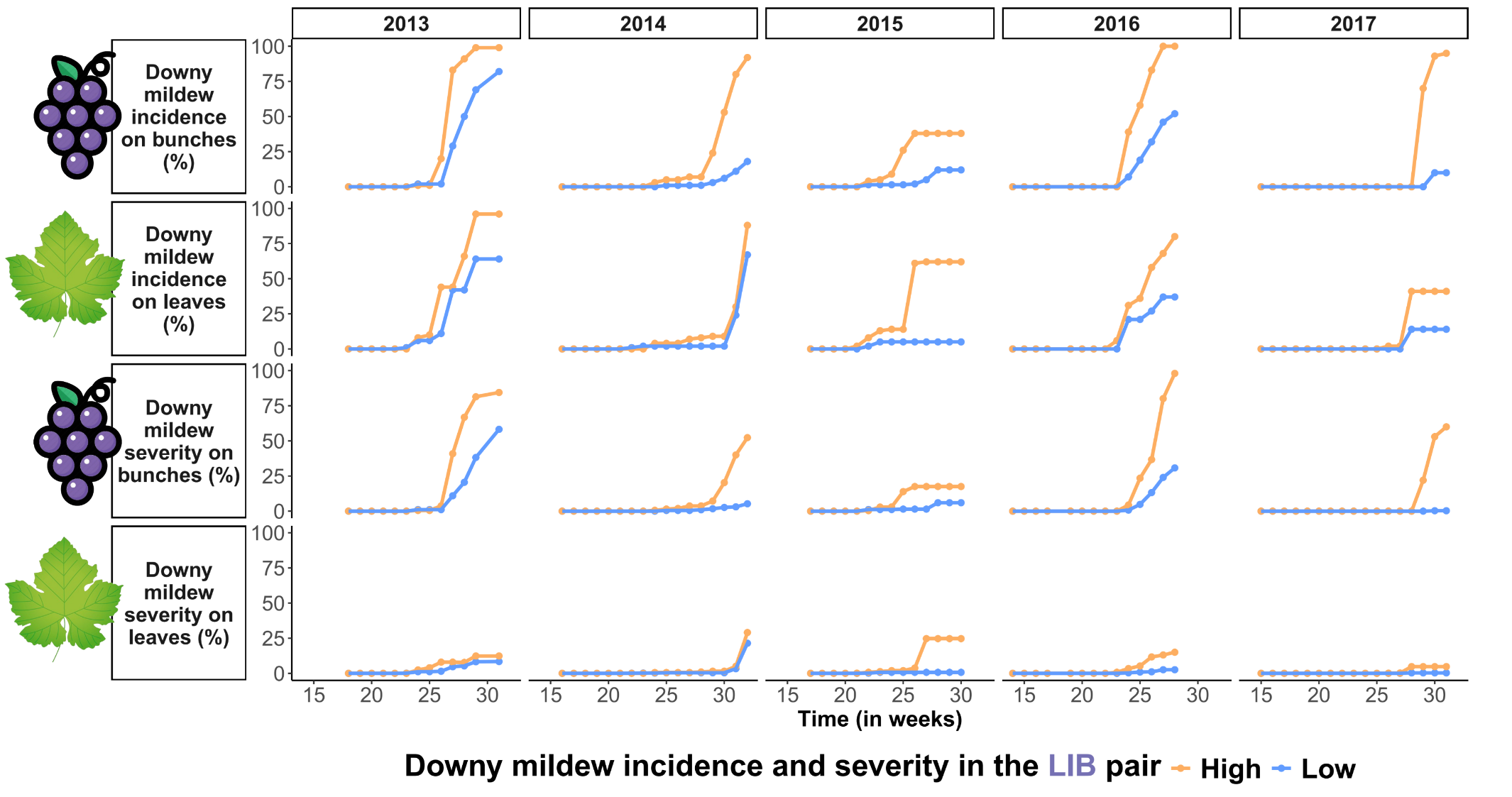

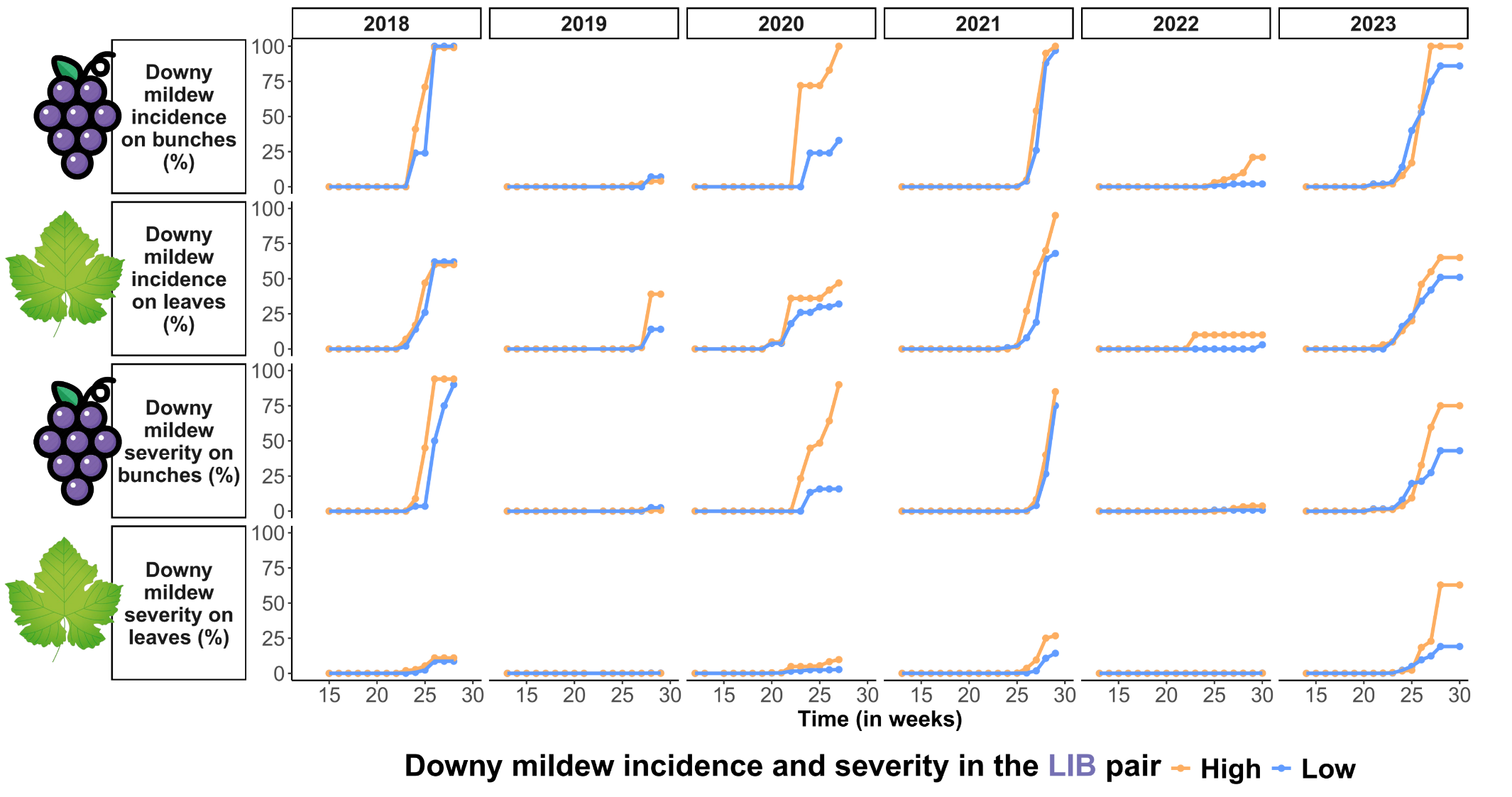

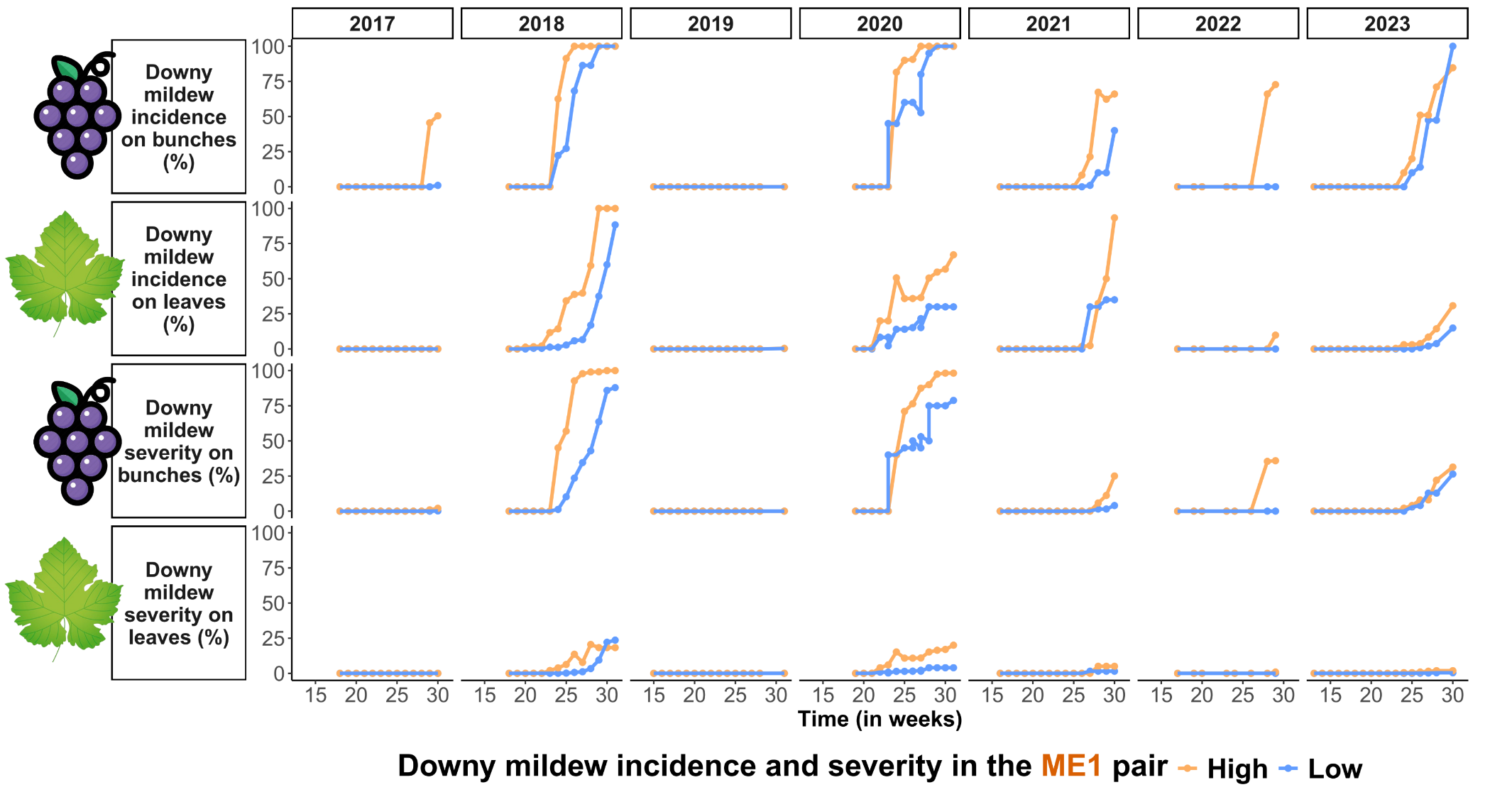

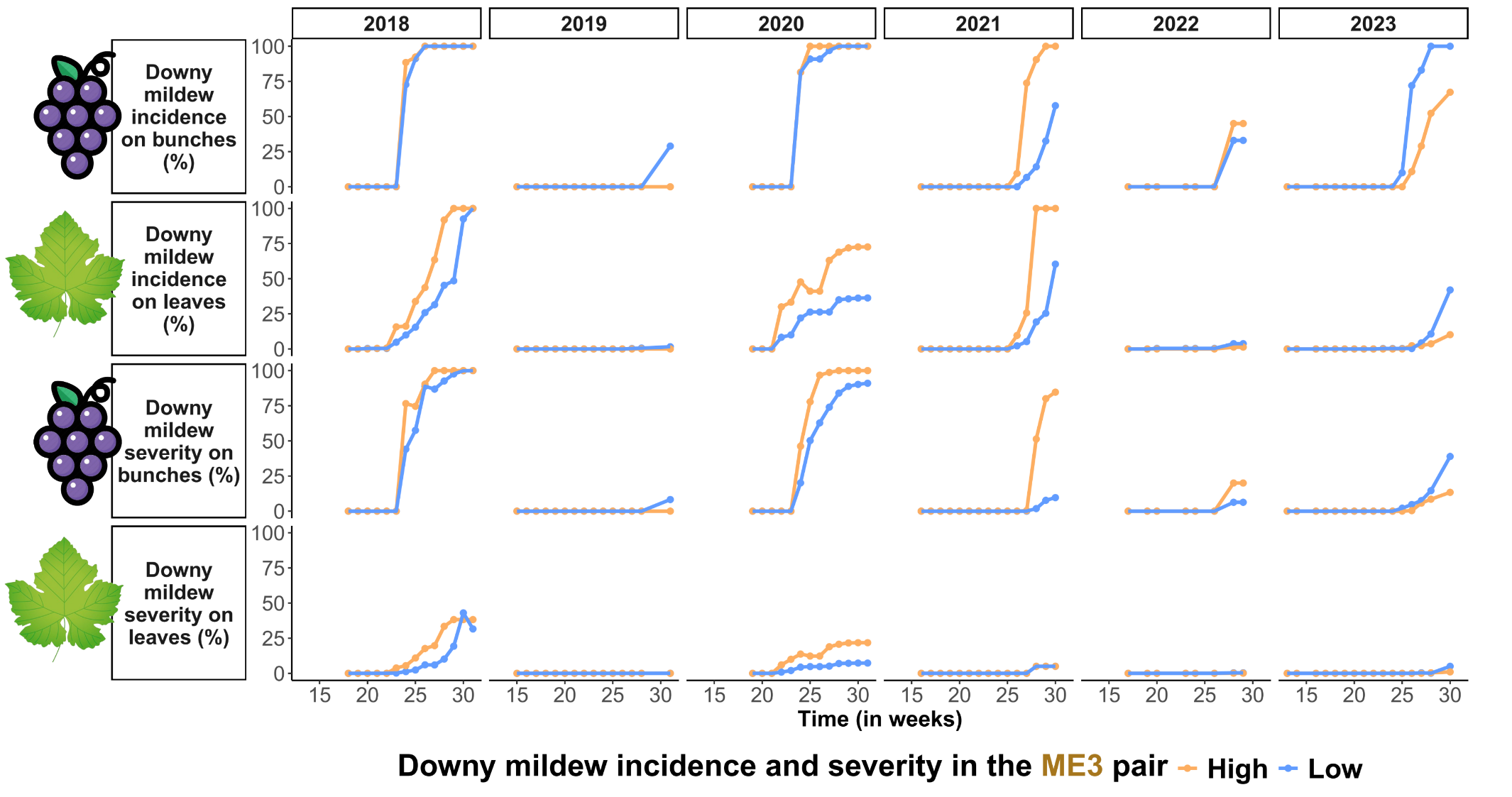


### Figure S7: Variation in downy mildew primary inoculum in topsoil.

The figure shows the concentration of *P. viticola* DNA in topsoil as a function of the incidence and severity of downy mildew in vineyard plots, the year of sampling, whether the topsoil sample was collected at the edge of the plot, and whether fungicides were applied to the sampled area in the year before sampling. The statistical significance of the linear mixed effects models is indicated as follows: ns (not significant). (nonsignificant trend), *, (p<0.05), ** (p<0.01), *** (p<0.001).


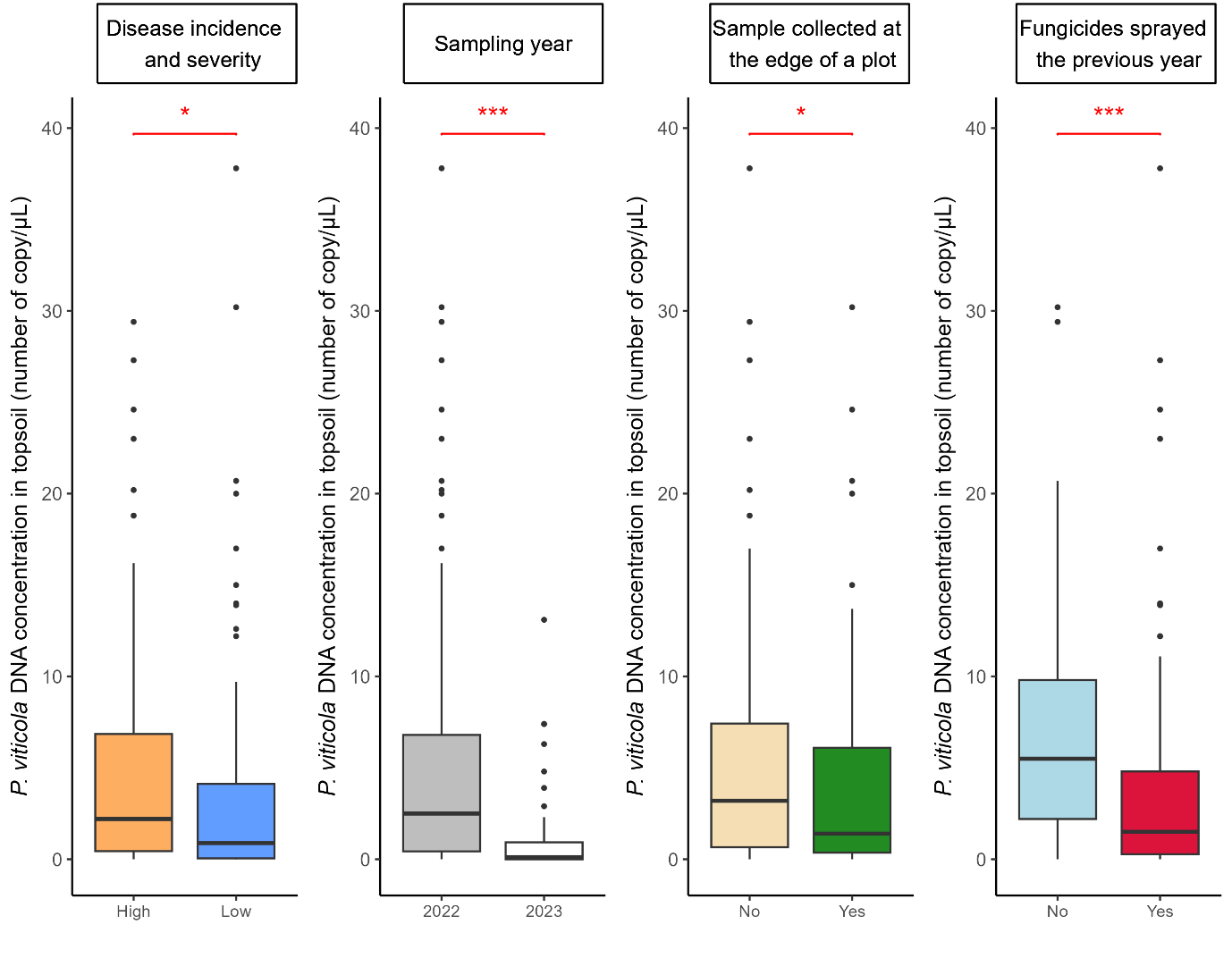


### **Figure S8: Microbial community profiles depending on downy mildew** incidence and severity **in vineyard plots.**

(A) Fungal and (B) bacterial community profiles of the topsoil, phyllosphere and leaf endosphere, representing the relative abundance of the different phyla averaged over the four samples collected in each plot and each microbial habitat during the 2023 sampling campaign. Phyla representing less than 1% of the sequences were grouped into the "Other" category.


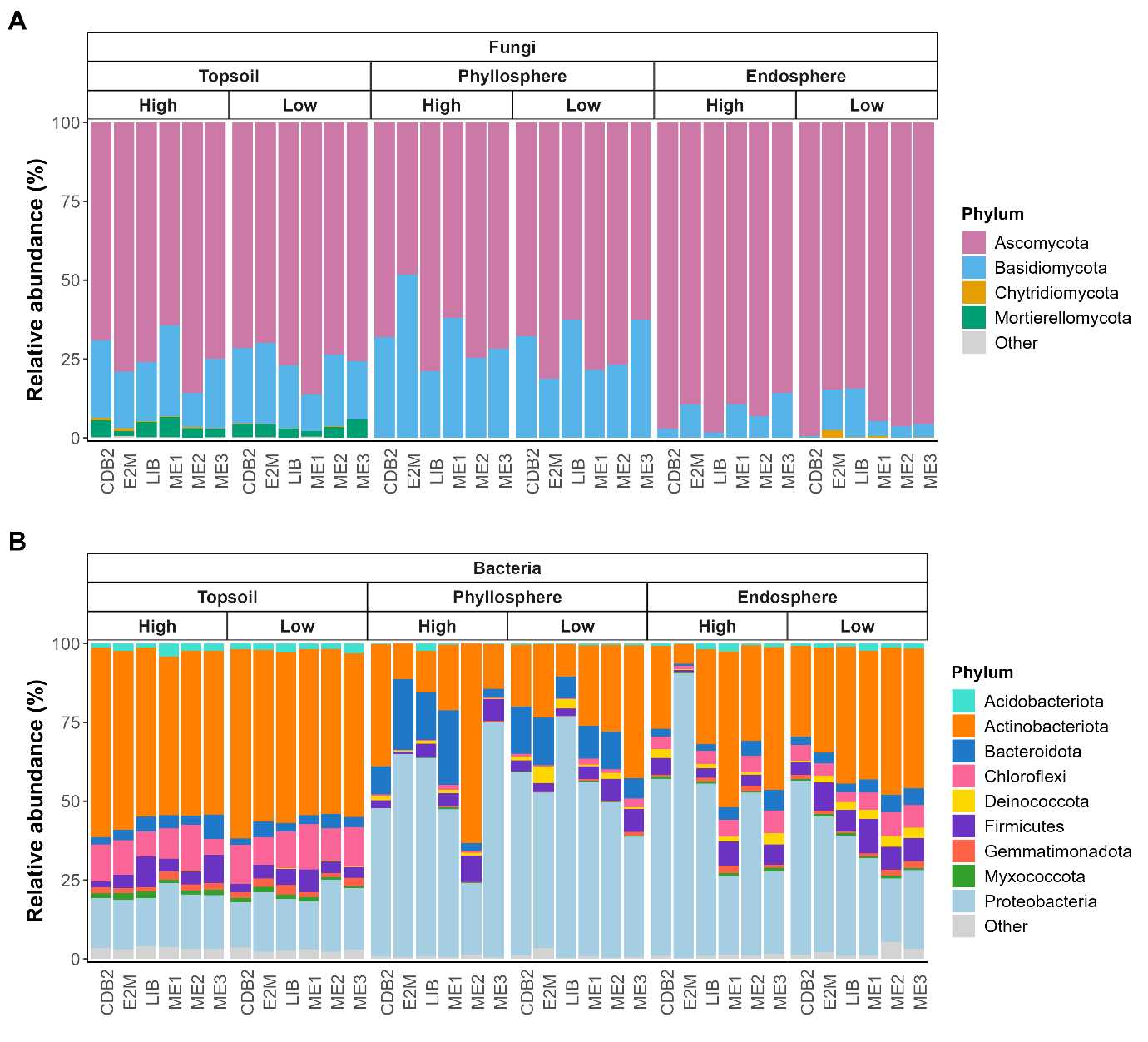


### Figure S9: Variation in microbial community composition across vineyard plots.

Dissimilarities among (A) fungal and (B) bacterial communities of the topsoil, phyllosphere and leaf endosphere for the five pairs of plots sampled in 2023, represented with a Principal Component Analysis (PCA). Samples collected from the same pair of plots are represented with the same color, with symbols indicating high and low downy mildew incidence and severity (triangles and circles, respectively).


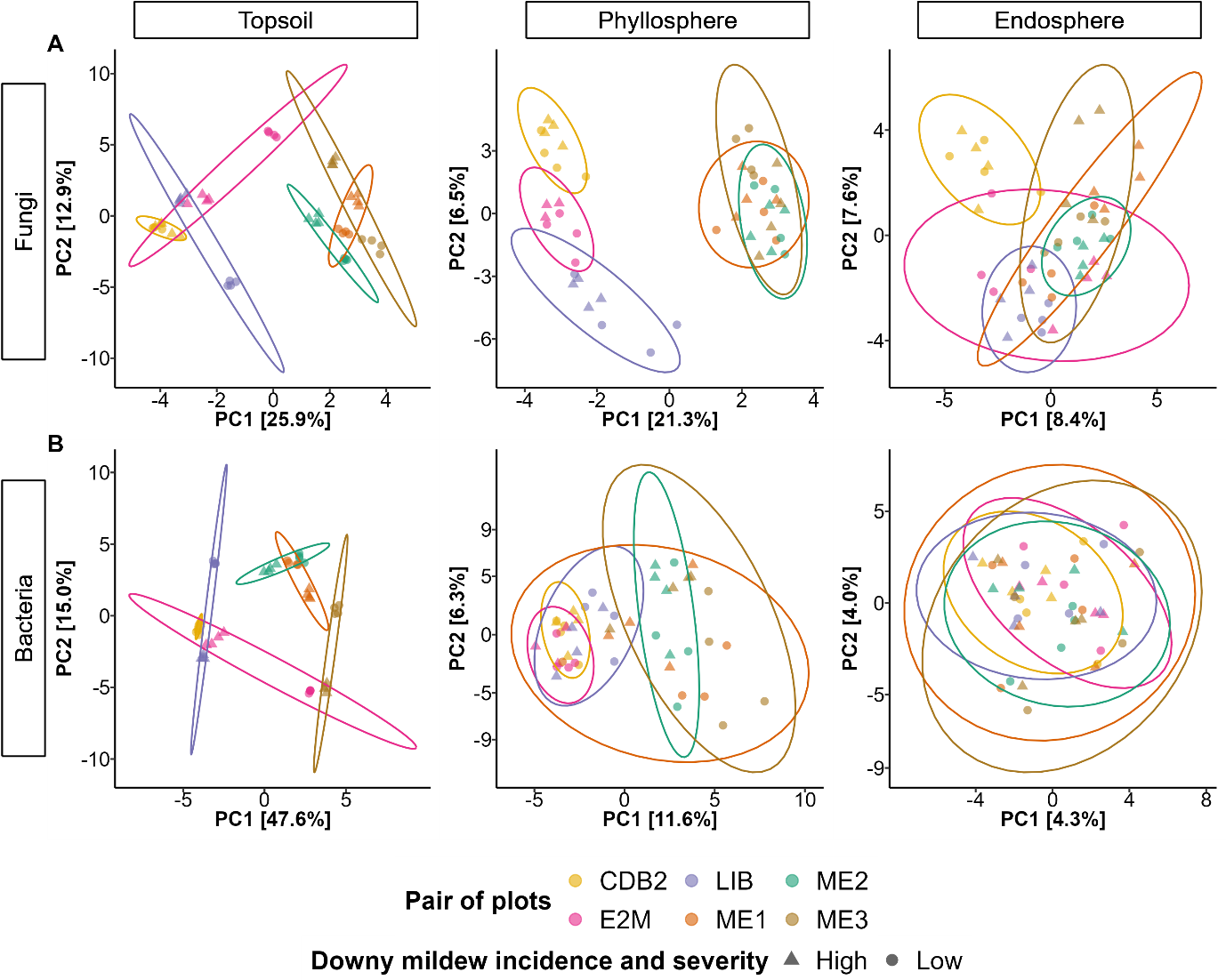


### **Figure S10: Environmental factors driving variation in microbial community composition in 2022.**

The figure shows the factors influencing the composition of the (A) fungal and (B) bacterial communities. The environmental variables were grouped into four categories (Soil, Management, Space, and Weather; Table S1) for variance partitioning. The Venn diagrams show the percentage of variance explained by each group of variables, either individually or in combination with other groups.


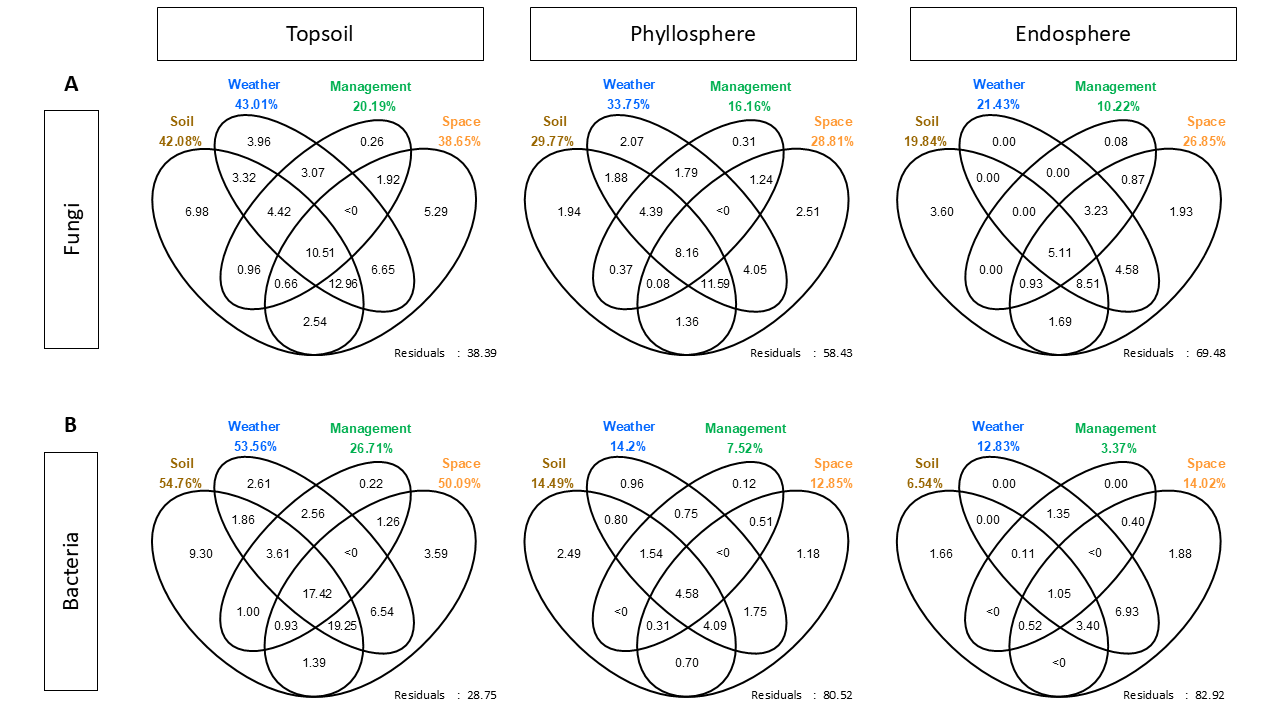


### **Figure S11: Environmental factors driving variation in microbial community composition in 2023.**

The figure shows the factors influencing the composition of the (A) fungal and (B) bacterial communities. The environmental variables were grouped into four categories (Soil, Management, Space, and Weather; Table S1) for variance partitioning. The Venn diagrams show the percentage of variance explained by each group of variables, either individually or in combination with other groups.

**
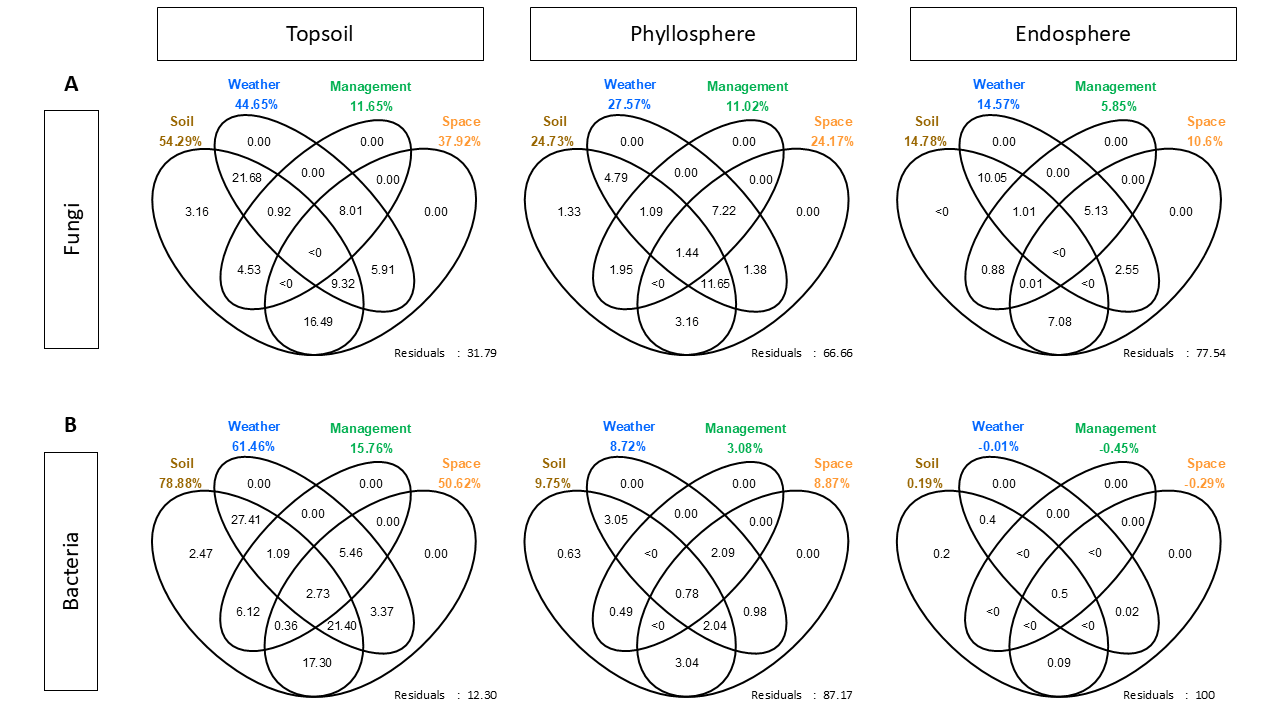
**

### **Figure S12: Variations** **in the diversity of microbial communities depending on downy mildew** incidence and severity **in vineyard plots (2022 data).**

The figure shows the diversity of (A) fungal and (B) bacterial communities in the topsoil, phyllosphere and endosphere of young grapevine leaves collected before the first phytosanitary treatment. The diversity indices are, from left to right, the observed total number of ASVs (Hill number q= 0), the exponential of the Shannon entropy index (Hill number q= 1), and the inverse of the Simpson concentration index (Hill number q= 2). The indices were calculated for each sample collected in 2022. The statistical significance of the linear mixed effects models is indicated as follows: ns (not significant). (nonsignificant trend), *, (p<0.05), ** (p<0.01), *** (p<0.001).

**
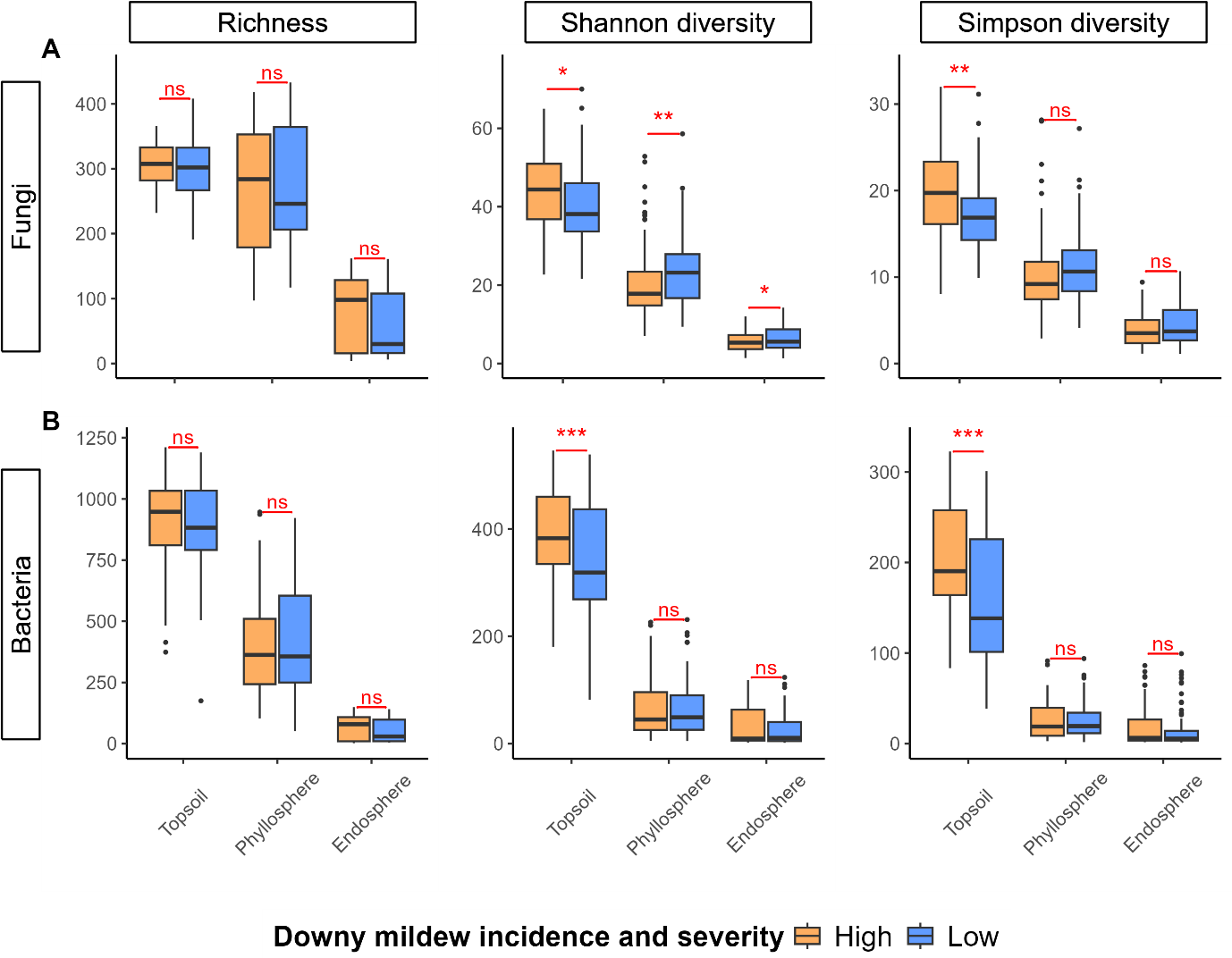
**

### **Figure S13: Variations in the diversity of microbial communities depending on downy mildew** incidence and severity **in vineyard plots (2023 data).**

The figure shows the diversity of (A) fungal and (B) bacterial communities in the topsoil, phyllosphere and endosphere of young grapevine leaves collected before the first phytosanitary treatment. The diversity indices are, from left to right, the observed total number of ASVs (Hill number q= 0), the exponential of the Shannon entropy index (Hill number q= 1), and the inverse of the Simpson concentration index (Hill number q= 2). The indices were calculated for each sample collected in 2022. The statistical significance of the linear mixed effects models is indicated as follows: ns (not significant). (nonsignificant trend), *, (p<0.05), ** (p<0.01), *** (p<0.001).


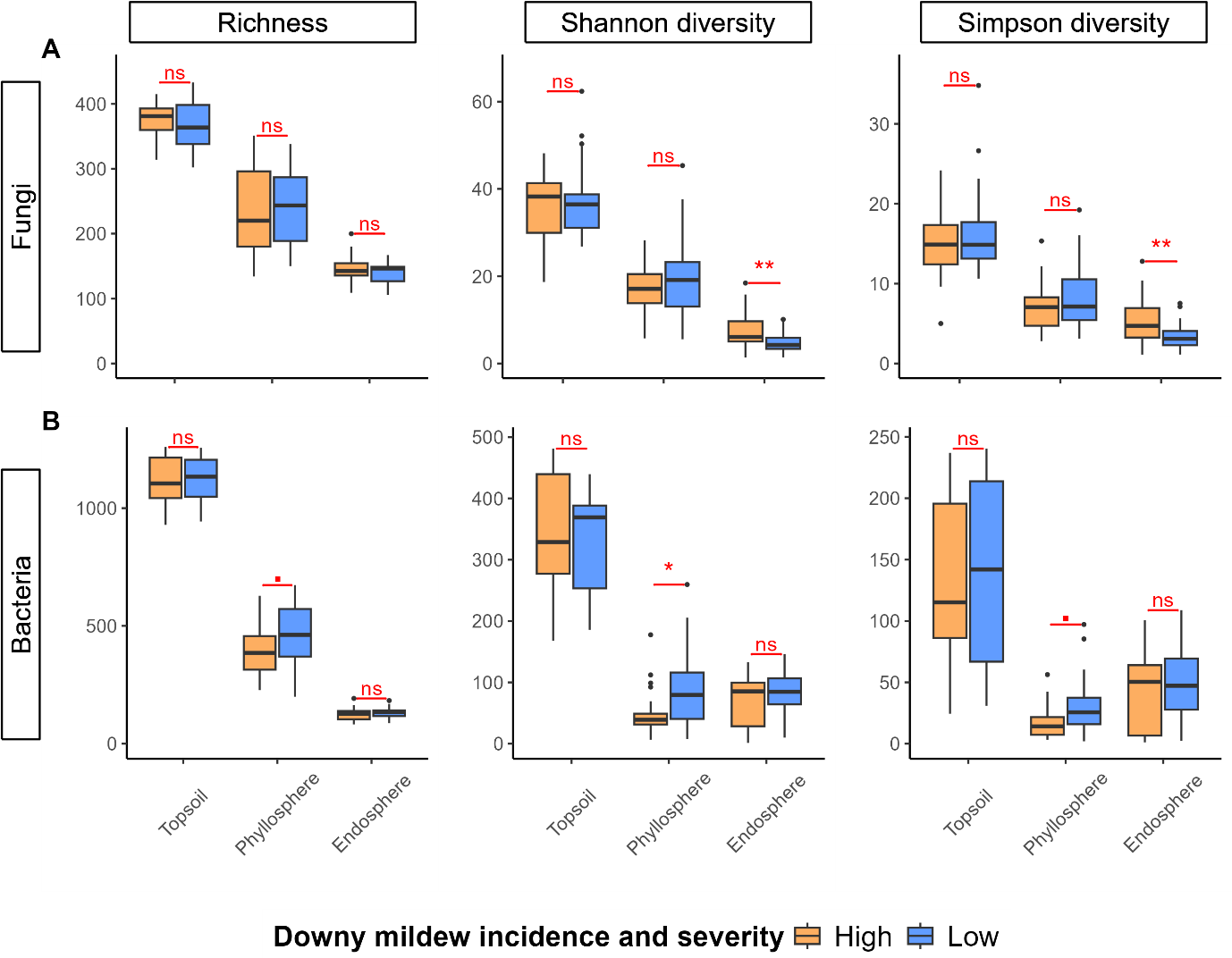


### **Figure S14:** **Endosphere fungal taxa that vary in abundance with downy mildew** incidence and severity**.**

For each condition (high *vs.* low incidence and severity), we represented the 20 Amplicon Sequence Variants (ASVs) that were significantly associated with the condition according to at least two methods and that had the highest average association scores. The five methods used to identify these ASVs were ANCOM-BC2 [21], Maaslin2 [22], LinDA [23], ZicoSeq [24] and Random Forest Classification [25]. The results are based on data collected in 2022.

**
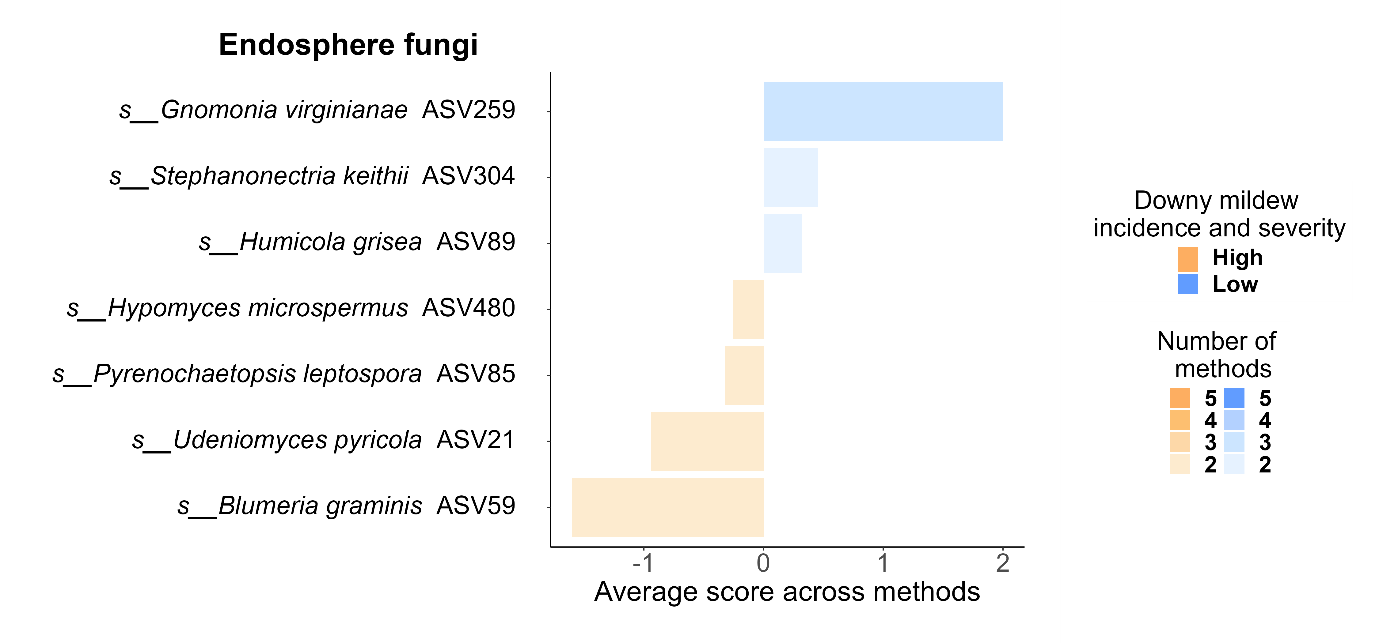
**

### **Figure S15: Topsoil fungal taxa that vary in abundance with downy mildew** incidence and severity**.**

For each condition (high vs. low incidence and severity), we represented the 20 Amplicon Sequence Variants (ASVs) that were significantly associated with the condition according to at least two methods and that had the highest average association scores. The five methods used to identify these ASVs are ANCOM-BC2 [21], Maaslin2 [22], LinDA [23], ZicoSeq [24] and Random Forest Classification [25]. The results are based on data collected in 2022.

**
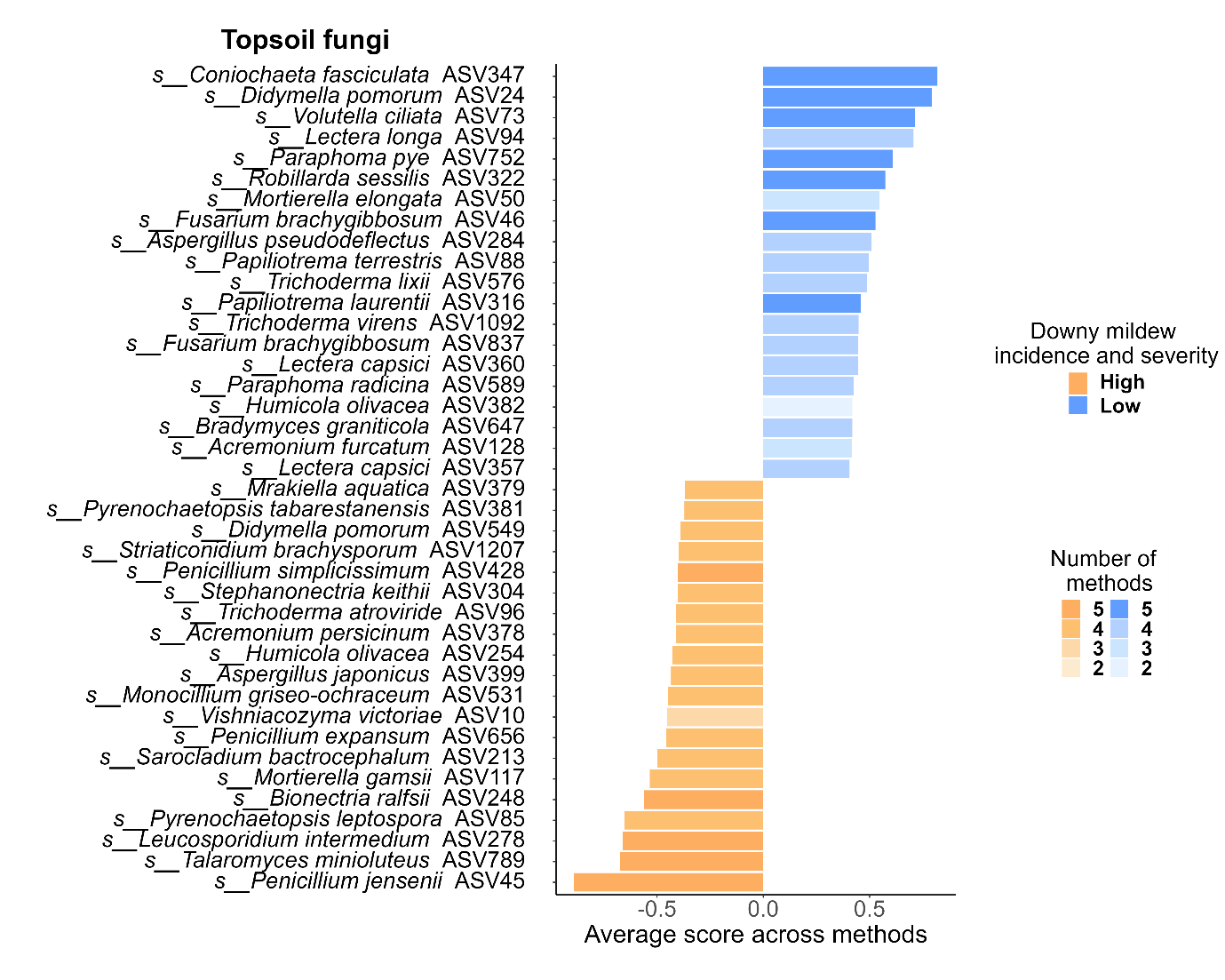
**

### **Figure S16: Phyllosphere bacterial taxa that vary in abundance with downy mildew** incidence and severity**.**

For each condition (high vs. low incidence and severity), we represented the 20 Amplicon Sequence Variants (ASVs) that were significantly associated with the condition according to at least two methods and that had the highest average association scores. The five methods used to identify these ASVs are ANCOM-BC2 [21], Maaslin2 [22], LinDA [23], ZicoSeq [24] and Random Forest Classification [25]. The results are based on data collected in 2022.


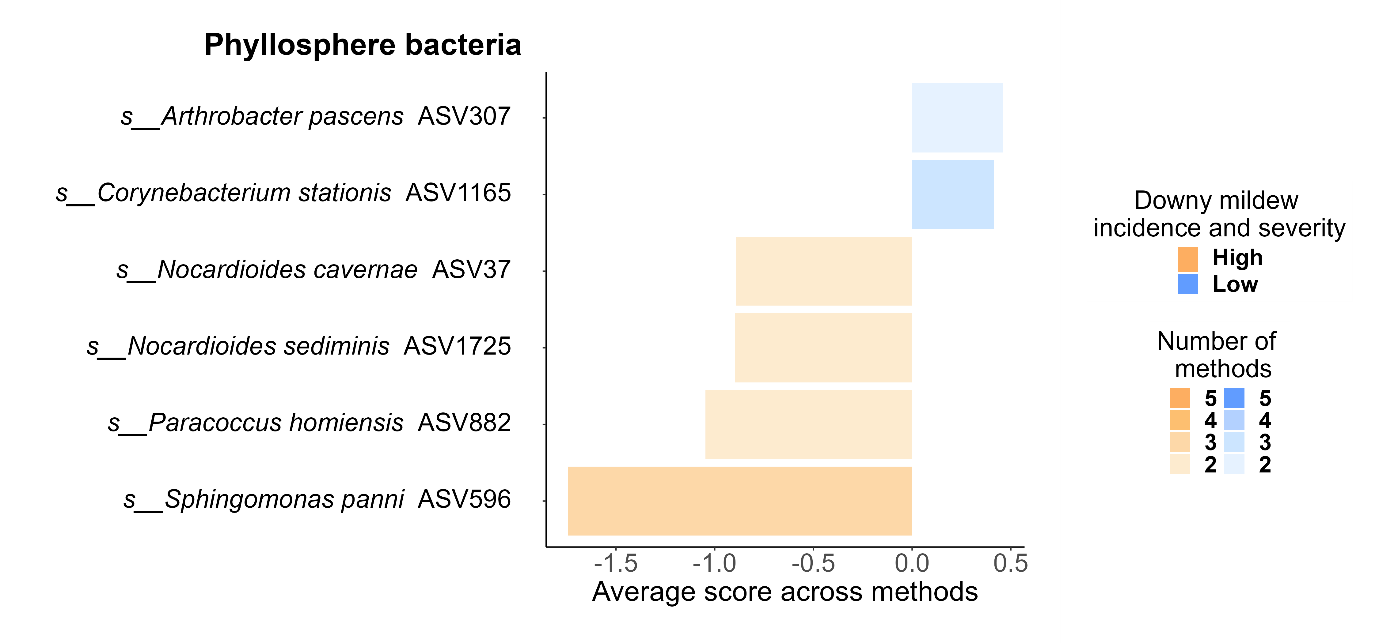


### **Figure S17: Random Forest algorithm performance in predicting grapevine** downy mildew incidence and severity **using leaf microbiota composition.**

Each dot represents the mean sensitivity and precision obtained for a given subset of the phyllosphere or endosphere microbiota data collected in 2022. The subsets differ in their composition (all fungi, all bacteria, all taxa or only abundant ones) and their level of taxonomic aggregation (from no aggregation of the ASVs to aggregation to the class level). The colored dots are those obtained with the optimal *mtry* value (*i.e.*, the value that predicts low downy mildew incidence and severity with the lowest error rate). The bars represent the standard deviation over the various iterations of the cross-validation step. The lowest error rate is indicated as a percentage. The analysis and figure use the scripts developed by [25].


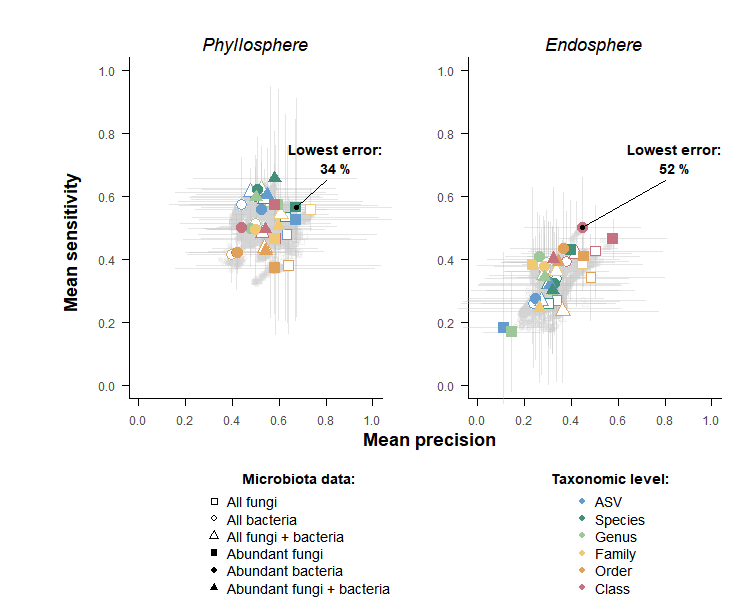


### **Figure S18: Random Forest algorithm performance in predicting grapevine** downy mildew incidence and severity **using topsoil microbiota data year-to-year.**

Each point represents the average sensitivity and accuracy obtained for a given subset of soil microbiota data collected in 2022 (training dataset) and 2023 (validation dataset). The subsets differ in their composition (all fungi, all bacteria, all taxa or only abundant ones) and their level of taxonomic aggregation (from no aggregation of the ASVs to aggregation to the class level). The colored dots are those obtained with the optimal mtry value (*i.e.*, the value that predicts low downy mildew incidence and severity with the lowest error rate). The bars represent the standard deviation over the various iterations of the cross-validation step. The lowest error rate is indicated as a percentage. The analysis and figure use the scripts developed by [25].


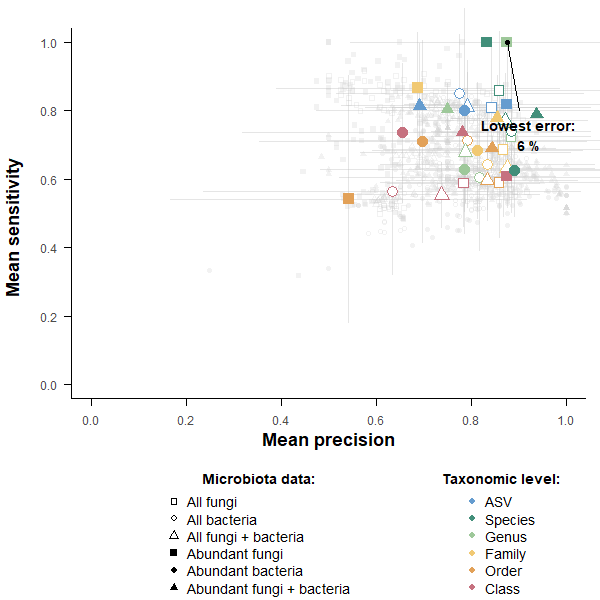


### **Figure S19: Random Forest algorithm performance in predicting grapevine** downy mildew incidence and severity **using leaf microbiota data year-to-year.**

Each point represents the average sensitivity and accuracy obtained for a given subset of the phyllosphere or endosphere microbiota data collected in 2022 (training dataset) and 2023 (validation dataset). The subsets differ in their composition (all fungi, all bacteria, all taxa or only abundant ones) and their level of taxonomic aggregation (from no aggregation of the ASVs to aggregation to the class level). The colored dots are those obtained with the optimal *mtry* value (*i.e.*, the value that predicts low downy mildew incidence and severity with the lowest error rate). The bars represent the standard deviation over the various iterations of the cross-validation step. The lowest error rate is indicated as a percentage. The analysis and figure use the scripts developed by [25].


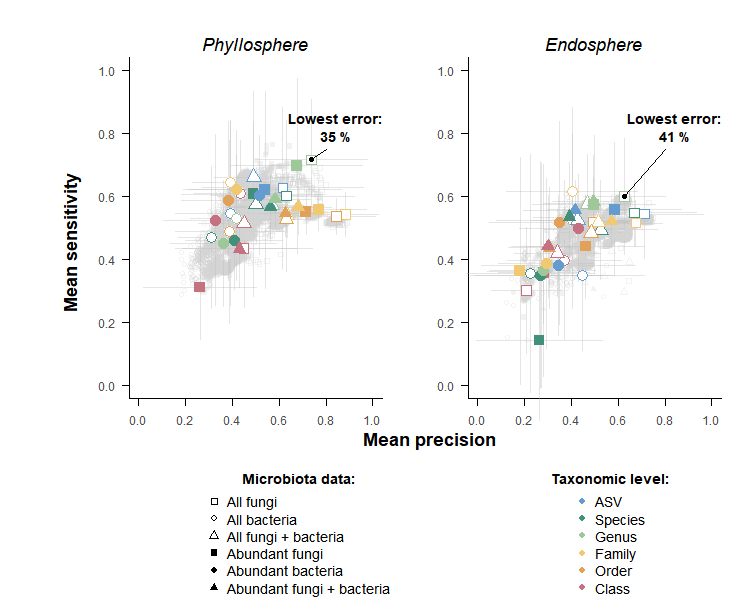


## Supplementary tables

### **Table S1: Environmental variables used to explain the variation in microbiota composition.**

The variables were grouped into four categories (Soil, Management, Space and Weather) to perform variance partitioning. The variables included in the analyses of the 2022 and 2023 datasets were the same, except for the variables ‘vine individual located near the plot edge (Yes/No)’ and ‘phytosanitary treatments applied the year before sampling (Yes/No)’. These variables were excluded from the 2023 analysis because we collected samples only from the center of each plot, which had been treated the year before.

| **Group** | **Explanatory variables** |
| --- | --- |
| Soil physical chemistry (*Soil*) | Clays (g/kg), sands (g/kg), silts (g/kg), C/N ratio, pH, organic matter (g/kg), total carbon (g/kg), total limestone (g/kg), total nitrogen (g/kg), total organic carbon (g/kg) |
| Vine variety and management (*Management*) | Vine variety (Cabernet-Sauvignon vs. Merlot Noir), management (Organic, Biodynamic, Conventional), whether fungicides were applied or not to the sampled area the year before sampling (Yes or No) |
| Position of the plot and position of the vine within the plot (*Space*) | Principal Coordinates of Neighborhood Matrix representing plot location (PCNM1 to PCNM5), within-plot location of the sample (edge vs. nonedge) |
| Weather during the month before sampling (*Weather*) | Total humidity (%), rainfall (mm) and temperature (°C) at 5 a.m., 10 a.m., 3 p.m. and 10 p.m. on the day before sampling, and at 5 a.m. and 10 a.m. on the day of sampling.  Maximum humidity (%), maximum insolation (hours), maximum temperature (°C) and total rainfall (mm) on the day of sampling, as well as averages of these measures for the 24 hours, 15 days and 30 days before sampling. |

### **Table S2: Factors driving variation in downy mildew inoculum in topsoil.**

We analyzed the effects of plot susceptibility to downy mildew, sampling year, sample location (whether on the plot edge or not), and application of fungicide treatment the year before sampling on the *Plasmopara viticola* DNA concentration in topsoil samples. R²m and R²c correspond to the marginal and conditional coefficients of determination of the model, respectively. N corresponds to the number of topsoil samples included in the model. The significance of the variables is as follows: ns (not significant). (nonsignificant trend: 0.1>p>0.05), * (p<0.05), ** (p<0.01), *** (p<0.001).

| **Model** | | | **Susceptibility to downy mildew**  **(High or Low)** | | **Sampling year**  **(2022 or 2023)** | | **Vine located to the edge plot**  **(Yes or No)** | | **Vine treated with fungicides the previous year**  **(Yes or No)** | | **Interaction between location and fungicide treatment** | |
| --- | --- | --- | --- | --- | --- | --- | --- | --- | --- | --- | --- | --- |
| N | R²c | R²m | p | Variation direction | p | Variation direction | p | Variation direction | p | Variation direction | p | Variation direction |
| 208 | 0.234 | 0.227 | * | High> Low | *** | 2022>2023 | * | No > Yes | *** | No > Yes | ns | - |

### **Table S3: Summary of read and ASV loss during data processing.**

The table shows the number of sequences and ASVs at different stages of the bioinformatic pipeline, including data decontamination using the metabaR R package v1.0.0 [1]. The "Input fasta file" refers to the raw, unmerged fasta file provided by the sequencing facility. "Reads processed" indicates the number of reads successfully assembled, trimmed and dereplicated to form ASVs. "Chimera removed" indicates the number of ASVs that are not chimeras. "Rare ASVs filtered out" includes ASVs that represent at least 0.00005% of the sequences. "ITS1 subregion extracted" denotes ASVs containing the ITS1 region in their DNA sequence. "Taxonomically assigned" refers to ASVs with reliable affiliations corresponding to fungi or bacteria. "Dataset decontaminated" indicates ASVs not considered contaminants.

|  | **barcode region:**  fungal nrDNA ITS genes | | **barcode region:**  bacterial 16S rRNA | |
| --- | --- | --- | --- | --- |
|  | **Sequences** | **ASVs** | **Sequences** | **ASVs** |
| **Input fasta file** | 201,915,252 | x | 99,358,259 | x |
| **Reads processed** | 200,734,858 | 11,834,604 | 93,942,170 | 3,498,571 |
| **Chimera removed** | 200,199,438 | 11,704,682 | 87,655,868 | 495,519 |
| **Rare ASVs filtered out** | 175,672,262 | 929 | 72,005,795 | 1,654 |
| **ITS1 subregion extracted** | 175,217,886 | 924 | x | x |
| **Taxonomically assigned** | 160,840,561 | 799 | 47,536,337 | 1,588 |
| **Dataset decontaminated** | 136,681,217 | 795 | 32,678,727 | 1,561 |

### Table S4: Most abundant fungal species in the topsoil, phyllosphere and leaf endosphere of vineyard plots (2022 data).

The table represents the top ten most abundant fungal species in the 2022 dataset. Species in bold are also among the top ten most abundant species in 2023. Relative abundance (%) indicates the proportion of sequences assigned to the species relative to the total number of sequences in the dataset. Prevalence (%) indicates the percentage of samples where the species is present with at least one sequence.

| **Topsoil** | | | **Leaf phyllosphere** | | | **Leaf endosphere** | | |
| --- | --- | --- | --- | --- | --- | --- | --- | --- |
| **Fungal species** | **Relative abundance (%)** | **Prevalence (%)** | **Fungal species** | **Relative abundance (%)** | **Prevalence (%)** | **Fungal species** | **Relative abundance (%)** | **Prevalence (%)** |
| ***Gibberella intricans*** (Ascomycota) | 12.08 | 99.37 | ***Aureobasidium pullulans*** (Ascomycota) | 11.24 | 94.3 | ***Epicoccum nigrum*** (Ascomycota) | 15.67 | 61.88 |
| *Cladosporium cladosporioides* (Ascomycota) | 4.56 | 97.48 | ***Cladosporium cladosporioides*** (Ascomycota) | 9.61 | 99.37 | ***Alternaria alternata*** (Ascomycota) | 7.44 | 44.38 |
| ***Saitozyma podzolica*** (Basidiomycota) | 4.34 | 75.47 | ***Vishniacozyma carnescens*** (Basidiomycota) | 7.21 | 92.41 | *Botrytis cinerea* (Ascomycota) | 4.93 | 45 |
| ***Vishniacozyma victoriae*** (Basidiomycota) | 3.27 | 93.71 | *Itersonilia pannonica* (Basidiomycota) | 6.9 | 79.11 | ***Didymella negriana*** (Ascomycota) | 4.8 | 10 |
| ***Fusarium avenaceum*** (Ascomycota) | 2.25 | 96.86 | ***Cladosporium allicinum*** (Ascomycota) | 6.8 | 95.57 | *Hirsutella lecaniicola* (Ascomycota) | 3.19 | 6.25 |
| *Plectosphaerella cucumerina* (Ascomycota) | 2.06 | 91.19 | ***Vishniacozyma victoriae*** (Basidiomycota) | 6.17 | 99.37 | ***Blumeria graminis*** (Ascomycota) | 2.94 | 33.12 |
| ***Mortierella elongata*** (Mortierellomycota) | 1.74 | 84.91 | *Filobasidium stepposum* (Basidiomycota) | 5.31 | 99.37 | ***Alternaria infectoria*** (Ascomycota) | 2.47 | 38.12 |
| *Boeremia exigua* (Ascomycota) | 1.47 | 79.87 | *Udeniomyces pyricola* (Basidiomycota) | 4.41 | 73.42 | ***Itersonilia perplexans*** (Basidiomycota) | 2.29 | 19.38 |
| *Didymella pomorum* (Ascomycota) | 1.46 | 88.68 | ***Cladosporium ramotenellum*** (Ascomycota) | 3.83 | 84.81 | *Cladosporium cycadicola* (Ascomycota) | 2.17 | 30 |
| *Clonostachys rosea* (Ascomycota) | 1.43 | 94.97 | *Botrytis cinerea* (Ascomycota) | 2.42 | 92.41 | *Cladosporium allicinum* (Ascomycota) | 2.12 | 27.5 |
| **Total** | **34.66** |  | **Total** | **63.9** |  | **Total** | **48.02** |  |

### Table S5: Most abundant bacterial genera in the topsoil, phyllosphere and leaf endosphere of vineyard plots (2022 data).

The table represents the top ten most abundant bacterial genera in the 2022 dataset. Genera in bold are also among the top ten most abundant genera in 2023. Relative abundance (%) indicates the proportion of sequences assigned to the genera relative to the total number of sequences in the dataset. Prevalence (%) indicates the percentage of samples where the genera is present with at least one sequence.

| **Topsoil** | | | **Leaf phyllosphere** | | | **Leaf endosphere** | | |
| --- | --- | --- | --- | --- | --- | --- | --- | --- |
| **Bacterial genus** | **Relative abundance (%)** | **Prevalence (%)** | **Bacterial genus** | **Relative abundance (%)** | **Prevalence (%)** | **Bacterial genus** | **Relative abundance (%)** | **Prevalence (%)** |
| ***Nocardioides*** *(*Actinobacteriota) | 5.85 | 100 | ***Sphingomonas*** (Proteobacteria) | 18.13 | 99.38 | *Cutibacterium* (Actinobacteriota) | 17.17 | 82.5 |
| ***Bacillus*** (Firmicutes) | 3.91 | 100 | ***Pseudomonas*** (Proteobacteria) | 13.03 | 93.75 | *Rickettsiella* (Proteobacteria) | 10.46 | 3.75 |
| ***Rubrobacter*** (Actinobacteriota) | 3.28 | 81.25 | ***Hymenobacter*** (Bacteroidota) | 9.74 | 98.12 | *Chryseolinea* (Bacteroidota) | 2.26 | 0.62 |
| ***Gaiella*** (Actinobacteriota) | 2.82 | 100 | *Massilia* (Proteobacteria) | 7.11 | 99.38 | *Variovorax* (Proteobacteria) | 1.85 | 46.88 |
| ***Pseudarthrobacter*** (Actinobacteriota) | 2.52 | 98.12 | ***Methylobacterium-Methylorubrum*** (Proteobacteria) | 6.27 | 98.75 | ***Wolbachia*** (Proteobacteria) | 1.48 | 21.88 |
| ***Sphingomonas*** (Proteobacteria) | 2.44 | 100 | ***Nocardioides*** (Actinobacteriota) | 1.75 | 99.38 | *Thermoanaerobacterium* (Firmicutes) | 1.47 | 41.25 |
| ***Arthrobacter*** (Actinobacteriota) | 2.31 | 100 | *Pseudarthrobacter* (Actinobacteriota) | 1.62 | 83.12 | *Caulobacter* (Proteobacteria) | 1.33 | 25 |
| ***Blastococcus*** (Actinobacteriota) | 1.96 | 99.38 | *Friedmanniella* (Actinobacteriota) | 1.4 | 85.62 | *Nocardioides* (Actinobacteriota) | 1.29 | 50.62 |
| ***Streptomyces*** (Actinobacteriota) | 1.58 | 100 | ***Variovorax*** (Proteobacteria) | 1.32 | 91.88 | ***Bacillus*** (Firmicutes) | 1.23 | 43.75 |
| *Massilia*  (Proteobacteria) | 1.41 | 100 | *Spirosoma* (Bacteroidota) | 1.03 | 87.5 | *Rhizobacter* (Proteobacteria) | 1.17 | 20 |
| **Total** | **28.08** |  | **Total** | **61.4** |  | **Total** | **39.71** |  |

### **Table S6: Most abundant fungal species in the topsoil, phyllosphere and leaf endosphere of vineyard plots (2023 data).**

The table represents the top ten most abundant fungal species in the 2023 dataset. Species in bold are also among the top ten most abundant species in 2023. *Relative abundance (%)* indicates the proportion of sequences assigned to the species relative to the total number of sequences in the dataset. *Prevalence (%)* indicates the percentage of samples where the species is present with at least one sequence.

| **Topsoil** | | | **Leaf phyllosphere** | | | **Leaf endosphere** | | |
| --- | --- | --- | --- | --- | --- | --- | --- | --- |
| **Fungal species** | **Relative abundance (%)** | **Prevalence (%)** | **Fungal species** | **Relative abundance (%)** | **Prevalence (%)** | **Fungal species** | **Relative abundance (%)** | **Prevalence (%)** |
| ***Gibberella intricans*** (Ascomycota) | 11.57 | 100 | ***Aureobasidium pullulans*** (Ascomycota) | 23.63 | 97.87 | ***Didymella negriana*** (Ascomycota) | 10.6 | 31.25 |
| ***Saitozyma podzolica*** (Basidiomycota) | 3.72 | 93.75 | *Cryptovalsa ampelina* (Ascomycota) | 11.15 | 74.47 | ***Epicoccum nigrum*** (Ascomycota) | 7.92 | 56.25 |
| *Humicola sardiniae* (Ascomycota) | 3.37 | 100 | ***Vishniacozyma carnescens*** (Basidiomycota) | 7.01 | 95.74 | ***Alternaria alternata*** (Ascomycota) | 4.98 | 52.08 |
| *Solicoccozyma phenolica* (Basidiomycota) | 2.06 | 85.42 | *Diplodia seriata*  (Ascomycota) | 5.36 | 82.98 | *Golovinomyces artemisiae* (Ascomycota) | 4.29 | 22.92 |
| *Truncatella angustata* (Ascomycota) | 1.67 | 100 | ***Cladosporium cladosporioides***  (Ascomycota) | 3.18 | 91.49 | *Aureobasidium pullulans* (Ascomycota) | 4.27 | 87.5 |
| *Penicillium jensenii* (Ascomycota) | 1.52 | 93.75 | ***Cladosporium ramotenellum***  (Ascomycota) | 3.13 | 91.49 | ***Alternaria infectoria*** (Ascomycota) | 3.8 | 68.75 |
| *Neocosmospora rubicola* (Ascomycota) | 1.46 | 100 | ***Cladosporium allicinum*** (Ascomycota) | 2.11 | 76.6 | *Diplodia seriata* (Ascomycota) | 3.35 | 64.58 |
| ***Vishniacozyma victoriae*** (Basidiomycota) | 1.45 | 100 | ***Vishniacozyma victoriae*** (Basidiomycota) | 1.72 | 100 | ***Itersonilia perplexans*** (Basidiomycota) | 3 | 50 |
| ***Fusarium avenaceum*** (Ascomycota) | 1.32 | 100 | *Peniophora versicolor* (Basidiomycota) | 1.51 | 95.74 | ***Blumeria graminis*** (Ascomycota) | 2.92 | 58.33 |
| ***Mortierella elongata*** (Mortierellomycota) | 1.31 | 100 | *Itersonilia perplexans* (Basidiomycota) | 1.45 | 80.85 | *Cryptovalsa ampelina* (Ascomycota) | 2.28 | 75 |
| **Total** | **29.45** |  | **Total** | **60.25** |  | **Total** | **47.41** |  |

### Table S7: Most abundant bacterial genera in the topsoil, phyllosphere and leaf endosphere of vineyard plots (2023 data).

The table represents the top ten most abundant bacterial genera in the 2023 dataset. Genera in bold are also among the top ten most abundant genera in 2023. Relative abundance (%) indicates the proportion of sequences assigned to the genera relative to the total number of sequences in the dataset. Prevalence (%) indicates the percentage of samples where the genera is present with at least one sequence.

| **Topsoil** | | | **Leaf phyllosphere** | | | **Leaf endosphere** | | |
| --- | --- | --- | --- | --- | --- | --- | --- | --- |
| **Bacterial genus** | **Relative abundance** | **Prevalence** | **Bacterial genus** | **Relative abundance** | **Prevalence** | **Bacterial genus** | **Relative abundance** | **Prevalence** |
| ***Rubrobacter*** (Actinobacteriota) | 7.24 | 100 | ***Sphingomonas*** (Proteobacteria) | 11.22 | 100 | ***Wolbachia*** (Proteobacteria) | 32.26 | 47.92 |
| ***Nocardioides*** (Actinobacteriota) | 4.32 | 100 | ***Hymenobacter*** (Bacteroidota) | 9.56 | 97.87 | *Rubrobacter* (Actinobacteriota) | 2.6 | 97.92 |
| ***Bacillus*** (Firmicutes) | 3.65 | 100 | ***Methylobacterium-Methylorubrum*** (Proteobacteria) | 8.88 | 100 | ***Nocardioides*** (Actinobacteriota) | 2.24 | 97.92 |
| ***Gaiella*** (Actinobacteriota) | 2.84 | 100 | *Tepidiphilus* (Proteobacteria) | 8.62 | 95.74 | ***Bacillus*** (Firmicutes) | 1.73 | 95.83 |
| ***Pseudarthrobacter*** (Actinobacteriota) | 2.67 | 100 | ***Pseudomonas*** (Proteobacteria) | 4.14 | 100 | *Sphingomonas* (Proteobacteria) | 1.71 | 93.75 |
| ***Sphingomonas*** (Proteobacteria) | 2.43 | 100 | *Bifidobacterium* (Actinobacteriota) | 2.55 | 19.15 | *Pseudomonas* (Proteobacteria) | 1.62 | 43.75 |
| ***Arthrobacter*** (Actinobacteriota) | 2.41 | 100 | *Mycobacterium* (Actinobacteriota) | 2.48 | 97.87 | *Deinococcus* (Deinococcota) | 1.5 | 47.92 |
| ***Streptomyces*** (Actinobacteriota) | 2.4 | 100 | ***Nocardioides*** (Actinobacteriota) | 1.75 | 100 | *Pseudarthrobacter* (Actinobacteriota) | 1.45 | 93.75 |
| ***Blastococcus*** (Actinobacteriota) | 1.77 | 100 | *Erwinia* (Proteobacteria) | 1.72 | 63.83 | *Bifidobacterium* (Actinobacteriota) | 1.27 | 10.42 |
| *Mycobacterium* (Actinobacteriota) | 1.69 | 100 | ***Variovorax*** (Proteobacteria) | 1.52 | 95.74 | *Enhydrobacter* (Proteobacteria) | 1.26 | 37.5 |
| **Total** | **31.42** |  | **Total** | **52.44** |  | **Total** | **47.64** |  |

### **Table S8: Factors driving variation in microbiota alpha diversity (2022 data).**

We analyzed the effects of downy mildew incidence and severity, application of fungicide treatment the year before sampling, and sample location (whether on the plot edge or not ) and their interactions on the microbial diversity in the 2022 data. R²m and R²c correspond reciprocally to the marginal and conditional determination coefficient of the model. N represents the number of samples included in the model. The significance of the variables is as follows: ns (not significant). (nonsignificant trend: 0.1>p>0.05), *, (p<0.05), ** (p<0.01), *** (p<0.001).

| Model | | | | Downy mildew incidence and severity | | Vine located to the edge plot | | Vine treated with fungicides the previous year | | Edge : Fungicides | |
| --- | --- | --- | --- | --- | --- | --- | --- | --- | --- | --- | --- |
|  | N | R²c | R²m | p | Variation direction | p | Variation direction | p | Variation direction | p | Variation direction |
| Diversity index: Richness (Hill number, q = 0) | | | | | | | | | | | |
| Fungi in Topsoil | 159 | 0.222 | 0.055 | ns | - | ns | - | ** | No > Yes | ns | - |
| Fungi in Phyllosphere | 158 | 0.836 | 0.015 | ns | - | ** | No > Yes | ns | - | ns | - |
| Fungi in Endosphere | 160 | 0.810 | 0.047 | ns | - | *** | Yes > No | ns | - | ns |  |
| Bacteria in Topsoil | 160 | 0.577 | 0.011 | ns | - | ns | - | . | No > Yes | ns | - |
| Bacteria in Phyllosphere | 160 | 0.344 | 0.036 | ns | - | ns | - | . | No > Yes | . | - |
| Bacteria in Endosphere | 160 | 0.788 | 0.067 | ns | - | ** | Yes > No | * | No > Yes | ns | - |
| Diversity index: Shannon (Hill number, q = 1) | | | | | | | | | | | |
| Fungi in Topsoil | 159 | 0.321 | 0.225 | * | High > Low | * | Yes > No | *** | No > Yes | ** |  |
| Fungi in Phyllosphere | 158 | 0.441 | 0.077 | ** | Low > High | ns | - | ** | No > Yes | . |  |
| Fungi in Endosphere | 160 | 0.147 | 0.090 | * | Low > High | ** | Yes > No | ns | - | ns | - |
| Bacteria in Topsoil | 160 | 0.471 | 0.341 | *** | High > Low | *** | Yes > No | ns | - | * |  |
| Bacteria in Phyllosphere | 160 | 0.059 | 0.030 | ns | - | ns | - | ns | - | . |  |
| Bacteria in Endosphere | 160 | 0.487 | 0.038 | ns | - | * | Yes > No | ns | - | ns | - |
| Diversity index: Simpson (Hill number, q = 2) | | | | | | | | | | | |
| Fungi in Topsoil | 159 | 0.353 | 0.312 | ** | High > Low | ** | Yes > No | *** | No > Yes | *** |  |
| Fungi in Phyllosphere | 158 | 0.233 | 0.054 | ns | - | ns | - | . | No > Yes | * |  |
| Fungi in Endosphere | 160 | 0.109 | 0.053 | ns | - | * | Yes > No | ns | - | ns | - |
| Bacteria in Topsoil | 160 | 0.512 | 0.392 | *** | High > Low | *** | Yes > No | ns | - | * |  |
| Bacteria in Phyllosphere | 160 | 0.024 | 0.018 | ns | - | ns | - | ns | - | ns | - |
| Bacteria in Endosphere | 160 | 0.383 | 0.048 | ns | - | * | Yes > No | ns | - | ns | - |

#####

### **Table S9: Factors driving variation in microbiota alpha diversity (2023 data).**

We analyzed the effects of downy mildew incidence and severity on the microbial diversity in 2023. R²m and R²c correspond reciprocally to the marginal and conditional determination coefficient of the model. N represents the number of samples included in the model. The significance of the variables is as follows: ns (not significant). (nonsignificant trend: 0.1>p>0.05), *, (p<0.05), ** (p<0.01), *** (p<0.001).

| Model | | | | Downy mildew incidence and severity | |
| --- | --- | --- | --- | --- | --- |
|  | N | R²c | R²m | p | Variation direction |
| Diversity index: Richness (Hill number, q = 0) | | | | | |
| Fungi in Topsoil | 48 | 0.398 | 0.017 | ns | - |
| Fungi in Phyllosphere | 47 | 0.744 | 0.00 | ns | - |
| Fungi in Endosphere | 48 | 0.154 | 0.024 | ns | - |
| Bacteria in Topsoil | 48 | 0.865 | 0.001 | ns | - |
| Bacteria in Phyllosphere | 47 | 0.169 | 0.053 | . | Low > High |
| Bacteria in Endosphere | 48 | 0.102 | 0.017 | ns | - |
| Diversity index: Shannon (Hill number, q = 1) | | | | | |
| Fungi in Topsoil | 48 | 0.328 | 0.010 | ns | - |
| Fungi in Phyllosphere | 47 | 0.029 | 0.014 | ns | - |
| Fungi in Endosphere | 48 | 0.291 | 0.123 | ** | High > Low |
| Bacteria in Topsoil | 48 | 0.954 | 0.002 | ns | - |
| Bacteria in Phyllosphere | 47 | 0.214 | 0.103 | * | Low > High |
| Bacteria in Endosphere | 48 | 0.183 | 0.028 | ns | - |
| Diversity index: Simpson (Hill number, q = 2) | | | | | |
| Fungi in Topsoil | 48 | 0.281 | 0.018 | ns | - |
| Fungi in Phyllosphere | 47 | 0.418 | 0.024 | ns | - |
| Fungi in Endosphere | 48 | 0.210 | 0.112 | ** | High > Low |
| Bacteria in Topsoil | 48 | 0.632 | 0.004 | ns | - |
| Bacteria in Phyllosphere | 47 | 0.196 | 0.090 | . | Low > High |
| Bacteria in Endosphere | 48 | 0.229 | 0.023 | ns | - |

### **Table S10: Fungal taxa indicators of** **low downy mildew primary inoculum in topsoil.**

Fungal ASVs which increase significantly in abundance as the concentration of *P. viticola* DNA in the topsoil decreases, according to the Threshold Indicator Taxa Analysis (TITAN) method [26], and they have the highest z scores. These results are based on the 2022 topsoil data. Relative abundance (%) indicates the proportion of sequences assigned to the taxa relative to the total number of sequences in the dataset. Prevalence (%) indicates the percentage of samples where the taxa are present with at least one sequence.

| **Microbial habitat** | **Fungal ASV** | **TITAN**  **z score** | **Relative abundance (%)** | **Prevalence (%)** |
| --- | --- | --- | --- | --- |
| Topsoil | *Neocosmospora rubicola* ASV37 | 5.96 | 1.21 | 93.71 |
|  | *Penicillium decumbens* ASV66 | 5.8 | 0.56 | 75.47 |
|  | *Bradymyces graniticola* ASV647 | 5.45 | 0.02 | 11.95 |
|  | *Emericellopsis minima* ASV351 | 5.3 | 0.13 | 25.79 |
|  | *Volutella ciliata* ASV73 | 4.8 | 0.37 | 68.55 |
|  | *Paraphoma pye* ASV752 | 4.8 | 0.02 | 47.80 |
|  | *Pseudeurotium bakeri* ASV159 | 4.68 | 0.13 | 69.18 |
|  | *Ascochyta medicaginicola* ASV350 | 4.63 | 0.05 | 20.75 |
|  | *Trichoderma hamatum* ASV299 | 4.56 | 0.09 | 43.40 |
|  | *Paraphoma radicina* ASV589 | 4.46 | 0.01 | 64.78 |
| Phyllosphere | *Plagiostoma pulchellum* ASV784 | 5.96 | 0.00 | 27.85 |
|  | *Plectosphaerella cucumerina* ASV645 | 5.87 | 0.00 | 32.28 |
|  | *Curvularia hawaiiensis* ASV326 | 5.73 | 0.00 | 25.32 |
|  | *Cytospora prunicola* ASV575 | 5.34 | 0.01 | 26.58 |
|  | *Hormonema macrosporu*m ASV596 | 5.32 | 0.00 | 68.35 |
|  | *Tremella phaeophysciae* ASV523 | 5.27 | 0.03 | 49.37 |
|  | *Bullera alba* ASV242 | 4.04 | 0.17 | 83.54 |
|  | *Plectosphaerella cucumerina* ASV31 | 4.02 | 0.04 | 71.52 |
|  | *Penicillium dierckxii* ASV861 | 4.02 | 0.00 | 6.96 |
|  | *Torula acaciae* ASV805 | 3.83 | 0.01 | 53.16 |
| Endosphere | *Fusarium brachygibbosum* ASV46 | 4.59 | 0.00 | 16.88 |
|  | *Malassezia restricta* ASV932 | 3.91 | 0.03 | 18.75 |
|  | *Vishniacozyma dimennae* ASV79 | 3.19 | 0.00 | 13.75 |

### **Table S11: Bacterial taxa indicators of low downy mildew primary inoculum in topsoil.**

Bacterial ASVs which increase significantly in abundance as the concentration of *P. viticola* DNA in the topsoil decreases, according to the Threshold Indicator Taxa Analysis (TITAN) method [27], and they have the highest z scores. These results are based on the 2022 topsoil data. Relative abundance (%) indicates the proportion of sequences assigned to the taxa relative to the total number of sequences in the dataset. Prevalence (%) indicates the percentage of samples where the taxa are present with at least one sequence.

| **Microbial habitat** | **Bacterial ASV** | **TITAN**  **z score** | **Relative abundance (%)** | **Prevalence (%)** |
| --- | --- | --- | --- | --- |
| Topsoil | *Exiguobacterium sibiricum* ASV169 | 7.08 | 0.01 | 26.88 |
|  | *Streptomyces ambofaciens* ASV915 | 6.73 | 0.02 | 69.38 |
|  | *Bacillus megaterium* ASV21 | 6.72 | 1.23 | 92.50 |
|  | *Streptomyces albidoflavus* ASV875 | 6.05 | 0.02 | 66.25 |
|  | *Arthrobacter globiformis* ASV281 | 5.69 | 0.16 | 80.63 |
|  | *Arthrobacter globiformis* ASV64 | 5.57 | 0.62 | 81.88 |
|  | *Pseudarthrobacter equi* ASV1234 | 5.29 | 0.02 | 50.63 |
|  | *Arthrobacter globiformis* ASV791 | 5.19 | 0.07 | 10.00 |
|  | *Arthrobacter globiformis* ASV1353 | 5.11 | 0.03 | 10.00 |
|  | *Arthrobacter oryzae* ASV45 | 4.97 | 0.89 | 91.88 |
| Phyllosphere | *Clostridium magnum* ASV2013 | 8.73 | 0.00 | 8.13 |
|  | *Terribacillus goriensis* ASV1216 | 7.48 | 0.01 | 7.50 |
|  | *Arthrobacter globiformis* ASV281 | 7.45 | 0.03 | 38.75 |
|  | *Methylobacterium isbiliense* ASV1222 | 6.61 | 0.01 | 26.25 |
|  | *Arthrobacter globiformis* ASV791 | 6.59 | 0.01 | 10.00 |
|  | *Arthrobacter globiformis* ASV1353 | 6.38 | 0.00 | 8.75 |
|  | *Caulobacter vibrioides* ASV1261 | 5.93 | 0.01 | 13.75 |
|  | *Arthrobacter pascens* ASV307 | 5.3 | 0.04 | 43.75 |
|  | *Streptomyces albidoflavus* ASV875 | 5.15 | 0.01 | 26.25 |
|  | *Streptococcus oralis* ASV1602 | 5 | 0.07 | 31.25 |

## References

1. Tedersoo L, Tooming-Klunderud A, Anslan S. PacBio metabarcoding of Fungi and other eukaryotes: errors, biases and perspectives. New Phytol. 2018;217:1370–85.

2. Tedersoo L, Bahram M, Zinger L, Nilsson H, Kennedy P, Yang T, et al. Best practices in metabarcoding of fungi: from experimental design to results. preprint. Preprints; 2021.

3. Behrens FH, Fischer M. Evaluation of Different Phyllosphere Sample Types for Parallel Metabarcoding of Fungi and Oomycetes in *Vitis vinifera*. Phytobiomes J. 2022;:PBIOMES-11-21-0072-SC.

4. Savian F, Marroni F, Ermacora P, Firrao G, Martini M. A Metabarcoding Approach to Investigate Fungal and Oomycete Communities Associated with Kiwifruit Vine Decline Syndrome in Italy. Phytobiomes J. 2022;6:290–304.

5. Taberlet P, Bonin A, Zinger L, Coissac E. Environmental DNA: For Biodiversity Research and Monitoring. Oxford University Press; 2018.

6. Schütte UME, Abdo Z, Bent SJ, Shyu C, Williams CJ, Pierson JD, et al. Advances in the use of terminal restriction fragment length polymorphism (T-RFLP) analysis of 16S rRNA genes to characterize microbial communities. Appl Microbiol Biotechnol. 2008;80:365–80.

7. Reysenbach A-L, Pace NR. Reliable amplification of hyperthermophilic archaeal 16S rRNA genes by the polymerase chain reaction. Archaea Lab Man. 1995;:101–7.

8. Gardes M, Bruns TD. ITS primers with enhanced specificity for basidiomycetes - application to the identification of mycorrhizae and rusts. Mol Ecol. 1993;2:113–8.

9. White TJ. PCR protocols: a guide to methods and applications | CiNii Research. 1990.

10. Camacho C, Coulouris G, Avagyan V, Ma N, Papadopoulos J, Bealer K, et al. BLAST+: architecture and applications. BMC Bioinformatics. 2009;10:421.

11. Quast C, Pruesse E, Yilmaz P, Gerken J, Schweer T, Yarza P, et al. The SILVA ribosomal RNA gene database project: improved data processing and web-based tools. Nucleic Acids Res. 2013;41:D590–6.

12. Wang Q, Garrity GM, Tiedje JM, Cole JR. Naïve Bayesian Classifier for Rapid Assignment of rRNA Sequences into the New Bacterial Taxonomy. Appl Environ Microbiol. 2007;73:5261–7.

13. Nilsson RH, Larsson K-H, Taylor AFS, Bengtsson-Palme J, Jeppesen TS, Schigel D, et al. The UNITE database for molecular identification of fungi: handling dark taxa and parallel taxonomic classifications. Nucleic Acids Res. 2019;47:D259–64.

14. Escudié F, Auer L, Bernard M, Mariadassou M, Cauquil L, Vidal K, et al. FROGS: Find, Rapidly, OTUs with Galaxy Solution. Bioinformatics. 2018;34:1287–94.

15. Tedersoo L, Bahram M, Põlme S, Kõljalg U, Yorou NS, Wijesundera R, et al. Global diversity and geography of soil fungi. Science. 2014;346:1256688.

16. F. Escapa I, Huang Y, Chen T, Lin M, Kokaras A, Dewhirst FE, et al. Construction of habitat-specific training sets to achieve species-level assignment in 16S rRNA gene datasets. Microbiome. 2020;8:65.

17. Sabat AJ, van Zanten E, Akkerboom V, Wisselink G, van Slochteren K, de Boer RF, et al. Targeted next-generation sequencing of the 16S-23S rRNA region for culture-independent bacterial identification - increased discrimination of closely related species. Sci Rep. 2017;7:3434.

18. Anslan S, Nilsson RH, Wurzbacher C, Baldrian P, Leho Tedersoo, Bahram M. Great differences in performance and outcome of high-throughput sequencing data analysis platforms for fungal metabarcoding. MycoKeys. 2018;:29–40.

19. Sommermann L, Geistlinger J, Wibberg D, Deubel A, Zwanzig J, Babin D, et al. Fungal community profiles in agricultural soils of a long-term field trial under different tillage, fertilization and crop rotation conditions analyzed by high-throughput ITS-amplicon sequencing. PLOS ONE. 2018;13:e0195345.

20. Brandt MI, Trouche B, Quintric L, Günther B, Wincker P, Poulain J, et al. Bioinformatic pipelines combining denoising and clustering tools allow for more comprehensive prokaryotic and eukaryotic metabarcoding. Mol Ecol Resour. 2021;21:1904–21.

21. Lin H, Peddada SD. Multigroup analysis of compositions of microbiomes with covariate adjustments and repeated measures. Nat Methods. 2024;21:83–91.

22. Mallick H, Rahnavard A, McIver LJ, Ma S, Zhang Y, Nguyen LH, et al. Multivariable association discovery in population-scale meta-omics studies. PLOS Comput Biol. 2021;17:e1009442.

23. Zhou H, He K, Chen J, Zhang X. LinDA: linear models for differential abundance analysis of microbiome compositional data. Genome Biol. 2022;23:95.

24. Yang L, Chen J. A comprehensive evaluation of microbial differential abundance analysis methods: current status and potential solutions. Microbiome. 2022;10:130.

25. Cambon MC, Trillat M, Lesur-Kupin I, Burlett R, Chancerel E, Guichoux E, et al. Microbial biomarkers of tree water status for next-generation biomonitoring of forest ecosystems. Mol Ecol. 2023;32:5944–58.

26. Baker ME, King RS, Kahle [aut D, cph, cre. TITAN2: Threshold Indicator Taxa Analysis. 2023.

27. Baker ME, King RS, Kahle D. TITAN2: Threshold Indicator Taxa Analysis. 2023.
